# Magnetically Induced Current Densities in π-Conjugated Porphyrin Nanoballs

**DOI:** 10.1021/acs.jpca.2c04856

**Published:** 2022-10-21

**Authors:** Atif Mahmood, Maria Dimitrova, Lukas N. Wirz, Dage Sundholm

**Affiliations:** Department of Chemistry, University of Helsinki, A. I. Virtasen Aukio 1, P.O. Box 55, FIN-00014 Helsinki, Finland

## Abstract

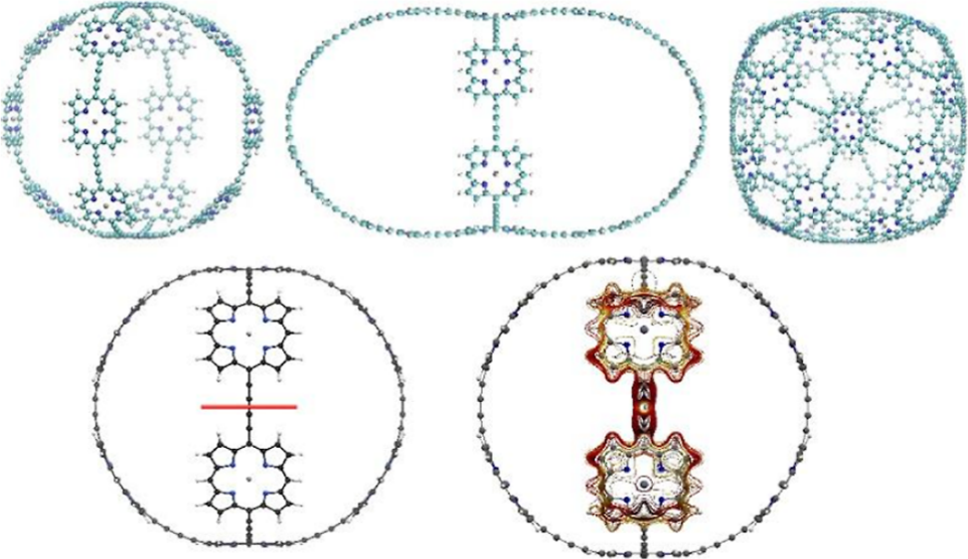

Magnetically induced current densities (MICDs) of Zn-porphyrinoid nanostructures have been studied at the density functional theory level using the B3LYP functional and the def2-SVP basis set. Six of the studied Zn-porphyrinoid nanostructures consist of two crossing porphyrinoid belts, and one is a porphyrinoid nanoball belonging to the octahedral (*O*) point group. The Zn-porphyrin units are connected to each other via butadiyne linkers as in a recently synthesized porphyrinoid structure resembling two crossed belts. The MICDs are calculated using the gauge-including magnetically induced current method. Current-density pathways and their strengths were determined by numerically integrating the MICD passing through selected planes that cross chemical bonds or molecular rings. The current-density calculations show that the studied neutral molecules are globally nonaromatic but locally aromatic sustaining ring currents only in the individual porphyrin rings or around two neighboring porphyrins. The ring-current strengths of the individual porphyrin rings are 20% weaker than in Zn-porphyrin, whereas oxidation leads to globally aromatic cations sustaining ring currents that are somewhat stronger than for Zn-porphyrin.

## 1 Introduction

Rational synthesis of three-dimensional (3D) π-conjugated molecular cages has been inspired by the discovery of the famous buckminsterfullerene (C_60_),^1^ which is probably the most significant of the experimentally known all-carbon nanostructures. A large number of fullerenes has been theoretically proposed, experimentally made, and detected.^2−9^ Other experimentally known or theoretically proposed all-carbon nanostructures of high symmetry comprise carbon nanotubes,^10^ toroidal carbon nanotubes,^11−14^ gaudiene,^15−17^ which are all derived from graphene, graphyne and graphdiyne^18^ and other general all-carbon cages.^19^ Now, we see a similar development in organic chemistry^20,21^ including the synthesis of porphyrin nanostructures, whose conjugation pathways, aromaticity, and magnetically induced current density (MICD) play a central role in their properties.^22−31^

Anderson et al. recently reported successful syntheses of porphyrinoid nanorings containing many porphyrin units connected with butadiyne linkers.^22,32^ They managed to prepare nanoball cages composed of two crossing porphyrin nanorings forming an ellipsoidal 3D structure. The synthesized porphyrin nanoball consists of 14 Zn-porphyrins linked with butadiyne units.^32^ The nanoball synthesis belongs to a larger research project, where they design, synthesize, and characterize porphyrinoid nanostructures.^22,24,26,27,32−44^ Osuka et al. have synthesized other kinds of conjugated porphyrin arrays, which have recently been reviewed.^45−49^

**Figure 1:**
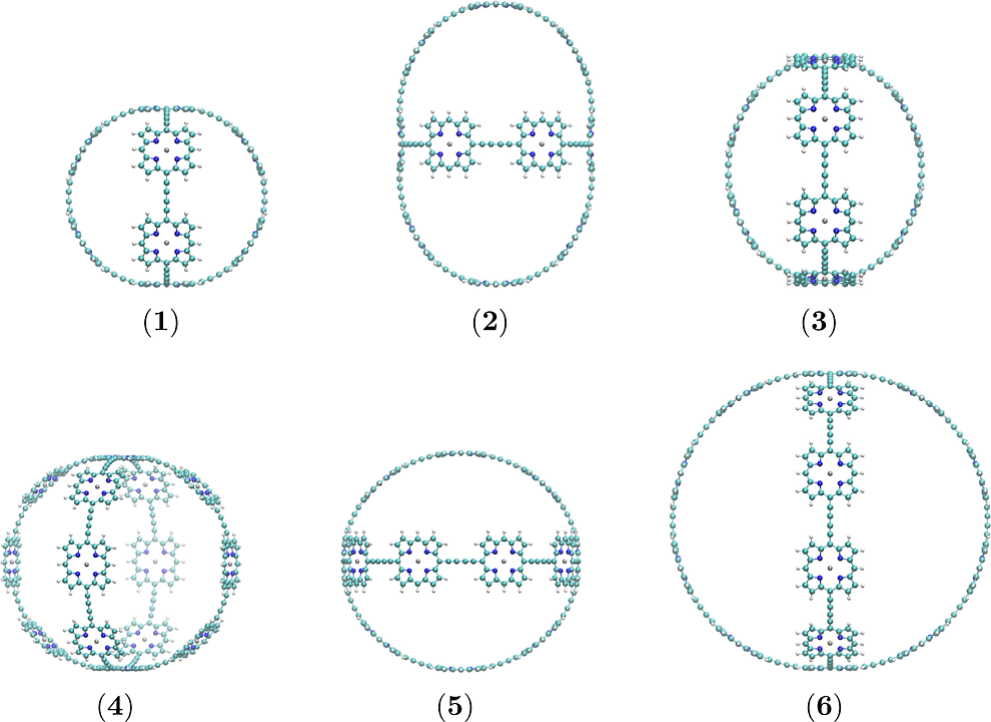
Structure of the investigated molecules **1**–**6**. Molecule **3** has been synthesized by Anderson et al.^32^

The synthesized porphyrin nanoball is, strictly speaking, not a ball^32^ since it actually consists of two butadiyne-linked Zn-porphyrin nanorings that cross and share two porphyrin units. One of the nanorings is made up of six butadiyne-linked porphyrin units. Perpendicular to it passes another butadiyne-linked porphyrin nanoring comprising 10 units. Since two of the porphyrin units belong to both nanorings, the total number of porphyrin units is 14. The nanoball notation b-P14·T6·(T4)_2_ introduced by Anderson et al. (see Section 2.2) tells that it can also be considered to consist of a nanoring with six units and two perpendicular half-rings composed of four porphyrin units each. This is how it was constructed experimentally.^32^ The molecular structure of b-P14·T6·(T4)_2_ (**3**) is shown in Figure 1.

In this work, we report the molecular structures and the magnetically induced current densities (MICDs) for the synthesized b-P14·T6·(T4)_2_ nanoball and for five analogous porphyrin structures with crossing nanorings of various sizes. We also propose a new nanoball which is not a crossed-belt structure, whose properties we have characterized computationally. The computational methods and a description of how the molecular structures were constructed are presented in Section 2. The MICD calculations are discussed in Section 3. Conclusions are drawn in Section 4.

## 2 Methods

### 2.1 Computational Levels

The initial molecular structures were constructed by defining the underlying polyhedra, where one porphyrin unit is represented by a degree-4 vertex and a butadiyne bridge is represented by an edge.^2,3,6,9,17^ These polyhedra were preoptimized using a simple force-field. The vertices and edges were then replaced by porphyrin units and butadiyne bridges, respectively. The missing hydrogen atoms were added. The resulting cages were optimized at a force-field level to give the initial molecular structures. Their geometry was subsequently optimized at the HF-3c level using the minix basis set in Turbomole with imposed symmetry.^50−52^ The final molecular structures were obtained at the BP86/def2-SVP/D3(BJ) level.^53−58^

Nuclear magnetic resonance (NMR) shielding tensors were calculated at the B3LYP/def2-SVP level of theory.^59^ The NMR shielding calculations produced the density matrix and the magnetically perturbed density matrices, which, together with the basis set and structural data, were used as input data for the calculations of the MICD with the gauge-including magnetically induced current method (GIMIC).^60−63^ The GIMIC program is freely available and interfaced to common quantum chemistry software packages including Turbomole, which we employed in this study.^64^

### 2.2 Molecular Structures

There are four *meso* hydrogen atoms in a porphyrin ring, which can be replaced by butadiyne linkers and form long chains of porphyrins. Six of the molecules included in this study resemble the synthesized nanoball labelled as b-P14·T6·(T4)_2_ (molecule **3**) by Anderson et al.^32^ In this notation, the letter b stands for ball; P14 stands for the total number of porphyrin units which are arranged such that the smaller nanoring consists of six porphyrin units (T6). In two of the porphyrin units comprising T6, the remaining two *meso* hydrogen atoms are replaced with butadiyne linkers belonging to two chains of porphyrins, in this case, each consisting of four units (hence T4). Essentially, this is equivalent to two nanorings that share two porphyrin units.

The molecules belonging to this series that we constructed include b-P10·T6·(T2)_2_ (**1**), that is, 10 Zn-porphyrin units arranged in a six-unit nanoring connected by two porphyrin chains of two units each. This is equivalent to two crossing nanorings of six units. The rest of the constructed molecules are b-P12·T6·(T3)_2_ (**2**), b-P14·T8·(T3)_2_ (**4**), b-P16·T8·(T4)_2_ (**5**), and b-P18·T10·(T4)_2_ (**6**) in the same notation. The molecular structures of the molecules are shown in Figure 1.

**Figure 2:**
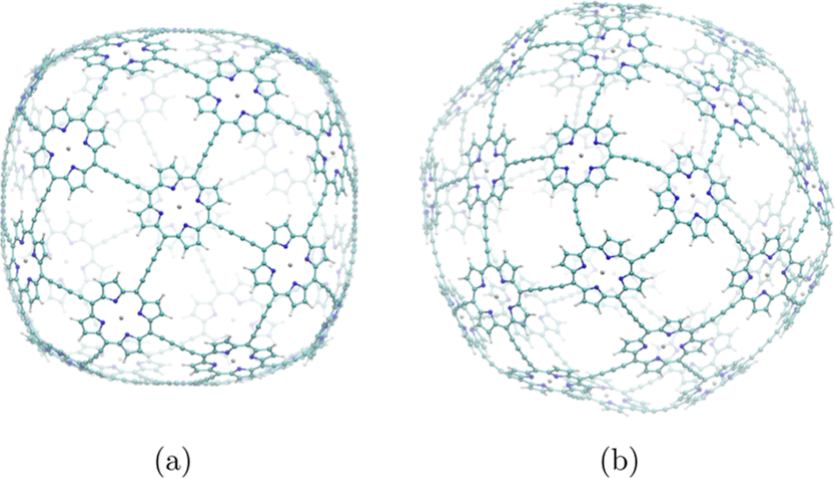
Molecular structure of the b-P_30_ cage (**7**) belonging to the octahedral (*O*) point group seen from two directions.

We also propose a butadiyne-linked porphyrin nanoball (**7**) that consists of 30 Zn-porphyrin units forming a cage structure (b-P30). Its molecular structure was constructed using a similar approach as reported by Fujita et al.^65^ The molecular structure of **7** shown in Figure 2 belongs to the *O* point group with two Zn porphyrin units centered on the main *C*_4_ axis. It is not a crossed-belt structure, but it can be seen as a distorted cube for which each side consists of five porphyrin units. One of them is in the center of the side, connected to the other four units via butadiyne linkers at the *meso* positions. The vertices of the cube are formed by the curvature produced by connecting three porphyrin units with butadiyne linkers at their *meso* carbon atoms.

### 2.3 Current Densities and Aromaticity

The aromatic character according to the magnetic criterion has been deduced from integrated ring-current strengths based on the ring-current model.^60,66−71^ The MICD represents the magnetic response of the molecular electronic structure.^63,71^ For a given orientation of the external magnetic field, the MICD tensor can be contracted, yielding the current density vector field for the specified magnetic field. The sign of the integrated current-density flux reflects its direction. Flow in the classical direction is positive, while the opposite direction is designated with a negative sign. The direction of the current-density flux, known as tropicity, is a global property. It can be unambiguously identified only by following the vector flux around the vortex. Vortices with a positive current-density flux are called diatropic, while those with a negative flux are paratropic. Paratropic contributions to the current density are a pure quantum mechanical effect.^62,63^

The strength of the current density is assumed to correlate with the degree of aromaticity. Molecules or molecular moieties sustaining a strong diatropic current density are aromatic, whereas a strong paratropic current density is associated with antiaromaticity. The strength can be calculated by placing an integration plane in specific places in the molecule. The current density is calculated numerically on grid points on the plane. The current density on the plane is then integrated, which gives the total flux that passes through the plane.^60^ Integration planes are typically positioned through a chemical bond to avoid the atomic domains with strong and sharp current densities near the nuclei. However, the strength is independent of the position of the integration plane because the charge conservation condition is fulfilled. Current-density profiles are obtained by dividing the integration plane into thin slices. The current density is integrated in each slice and plotted as a function of distance along the plane.

The current density can also be investigated visually by means of streamlines using, for example, the Paraview program,^72^ where a sample sphere of a given radius containing a number of grid points is employed for tracing the vector field of the current density using the Runge-Kutta method.^73,74^

## 3 Results of the Current-Density Calculations

### 3.1 Molecule **1**

**Figure 3:**
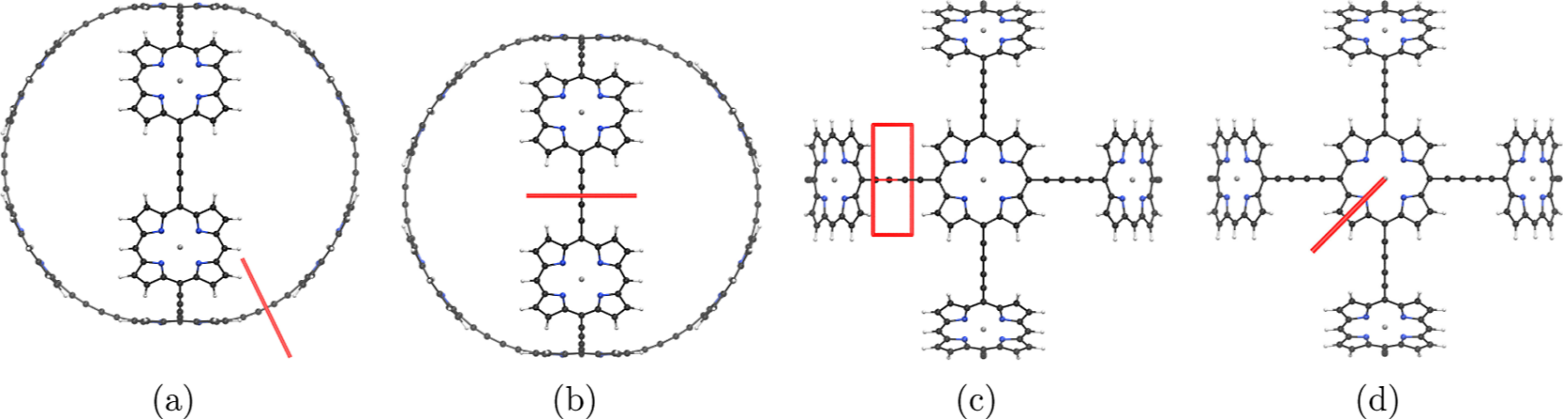
Position of the integration planes 1A–1D in molecule **1**. The magnetic field vector is parallel to the integration planes shown in red. The integrated MICD strengths are reported in Table 1.

The strength of the MICD of the neutral molecule **1** and its dication were investigated by numerical integration of the current-density flux passing through the planes shown in Figure 3a–c. The strength of the MICD in one of the porphyrin units was determined by placing the integration plane as shown in Figure 3d. The magnetic field is parallel to the integration planes except for plane 1C, which leans slightly with respect to the field. The net current strength through planes 1B and 1C vanishes because the current-density flux through them must vanish for symmetry reasons. The porphyrin units are expectedly locally aromatic, sustaining a ring current of 21.4 nA·T^–1^, which is 6 nA·T^–1^ weaker than for free-base porphyrin.^28,75^ The obtained MICD strengths are summarized in Table 1.

**Table 1:** Strength of the Diatropic and Paratropic Contributions (in nA·T^–1^) to the Net MICD Strength Passing Different Planes in Molecule **1** and Its Dication^a^

plane	diatropic	paratropic	net
Neutral			
1A	6.45	–8.85	–2.41
1B	7.63	–7.63	0.00
1C	6.85	–6.85	0.00
1D	36.03	–14.65	21.39
Dication			
1A	34.69	–0.11	34.58
1B	7.69	–7.69	0.00
1C	6.96	–6.96	0.00
1D	32.33	–15.78	16.55

a The planes are illustrated in Figure 3. The net current strength vanishes for planes 1B and 1C due to symmetry.

**Figure 4:**
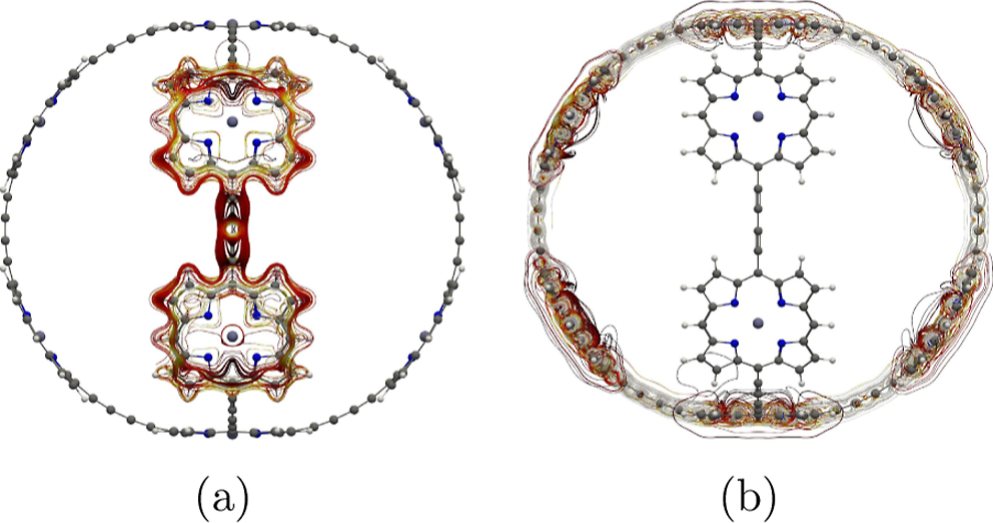
MICD pathways in (a) molecule **1** and (b) its dication. The magnetic field vector points toward the viewer. More MICD pictures are reported as the Supporting Information.

The neutral molecule is nonaromatic, sustaining a very weak paratropic ring-current of −2.4 nA·T^–1^, whereas the dication of **1** is strongly aromatic, sustaining a net diatropic ring-current of 34.6 nA·T^–1^. A similar kind of alternating aromatic character was also obtained in the study by Peeks et al.^22^ The contribution of the current density of the Zn atom equal to 40.8 nA·T^–1^ is not included in the reported ring-current strength of the porphyrin moiety. The MICD pathways of **1** in Figure 4a show that there are only local bond currents and a ring current around the porphyrin units.

The porphyrin units of the dication of **1** are slightly less aromatic than in the neutral molecule with an MICD strength of 16.6 nA·T^–1^. The Zn-atom contribution in the dication is the same as for neutral **1**. Figure 4b illustrates the MICD of the dication of **1** using streamlines.

### 3.2 Molecule **2**

**Figure 5:**
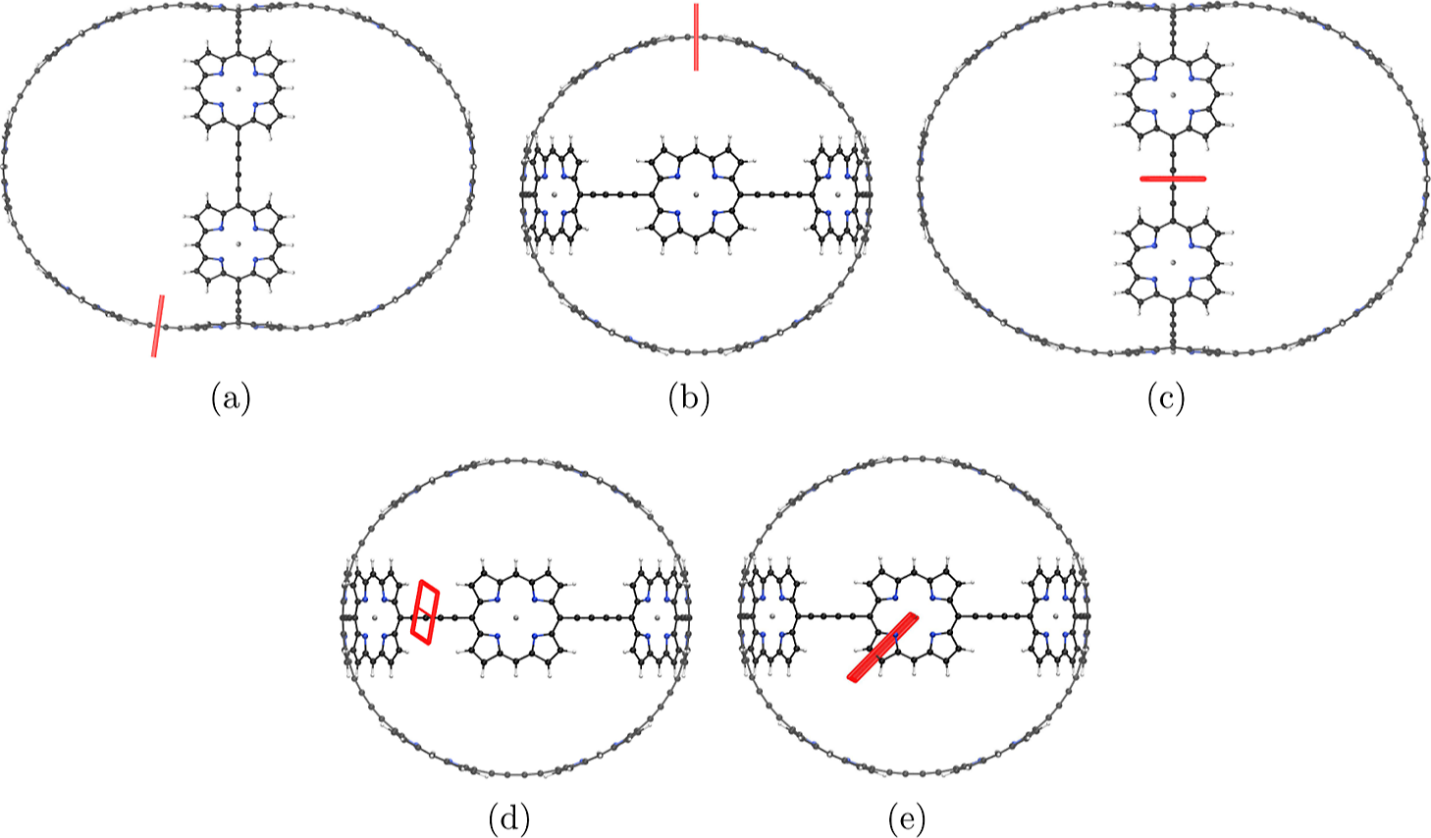
Position of the integration planes 2A–2E in molecule **2**. The integrated MICD strengths are reported in Table 2. The magnetic field vector is parallel to the integration planes shown in red.

**Figure 6:**
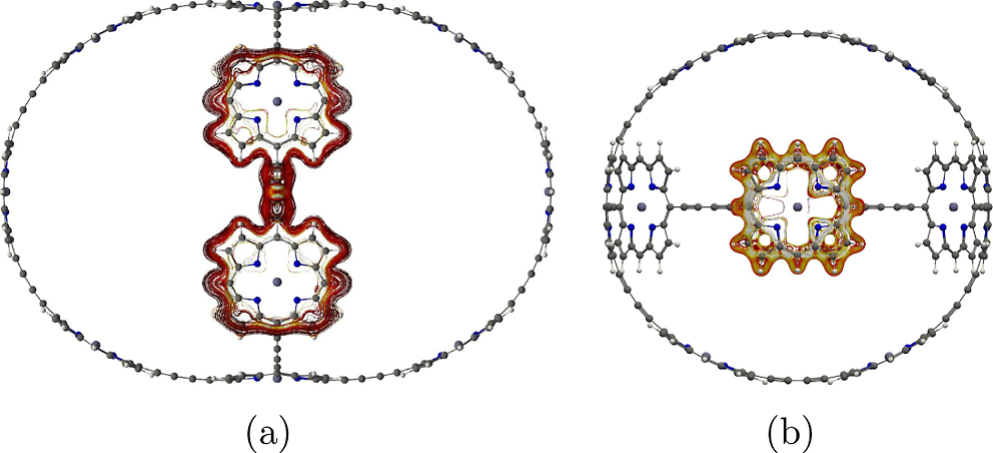
MICD pathways in different parts of molecule **2**. The magnetic field vector points toward the viewer. More MICD pictures are reported as the Supporting Information.

The MICD pathways of **2** were investigated by using the integration planes shown in Figure 5. It is nonaromatic, sustaining a vanishingly small global ring-current of −0.1 and −1.5 nA·T^–1^ in the two rings. The strength of the MICD passing plane 2C vanishes for symmetry reasons. The strengths of the MICD passing through the other integration planes are very small because they mainly consist of local MICD vortices as seen in Figure 6. The MICD of the porphyrin ring is 24.5 nA·T^–1^ when the atomic ring current around Zn of 34.6 nA·T^–1^ is omitted. The ring current of the porphyrin unit is 3 nA·T^–1^ weaker than for Zn porphyrin.^31^ The obtained MICD strengths are summarized in Table 2. The MICD pathways of **2** are shown as streamlines in Figure 6. The dication of **2** has not been studied because its energy gap between the highest occupied molecular orbital (HOMO) and the lowest unoccupied molecular orbital (LUMO) is very small.

**Table 2:** Strength of the Diatropic and Paratropic Contributions (in nA·T^–1^) to the Net MICD Strength Passing Different Planes in Molecule **2**^a^

plane	diatropic	paratropic	net
2A	7.62	–7.56	–0.06
2B	6.89	–8.36	–1.48
2C	7.57	–7.57	0.00
2D	4.39	–2.33	2.06
2E	37.65	–13.19	24.46

a The net current strength vanishes for plane 2C due to symmetry.

### 3.3 Molecule **3**

**Figure 7:**
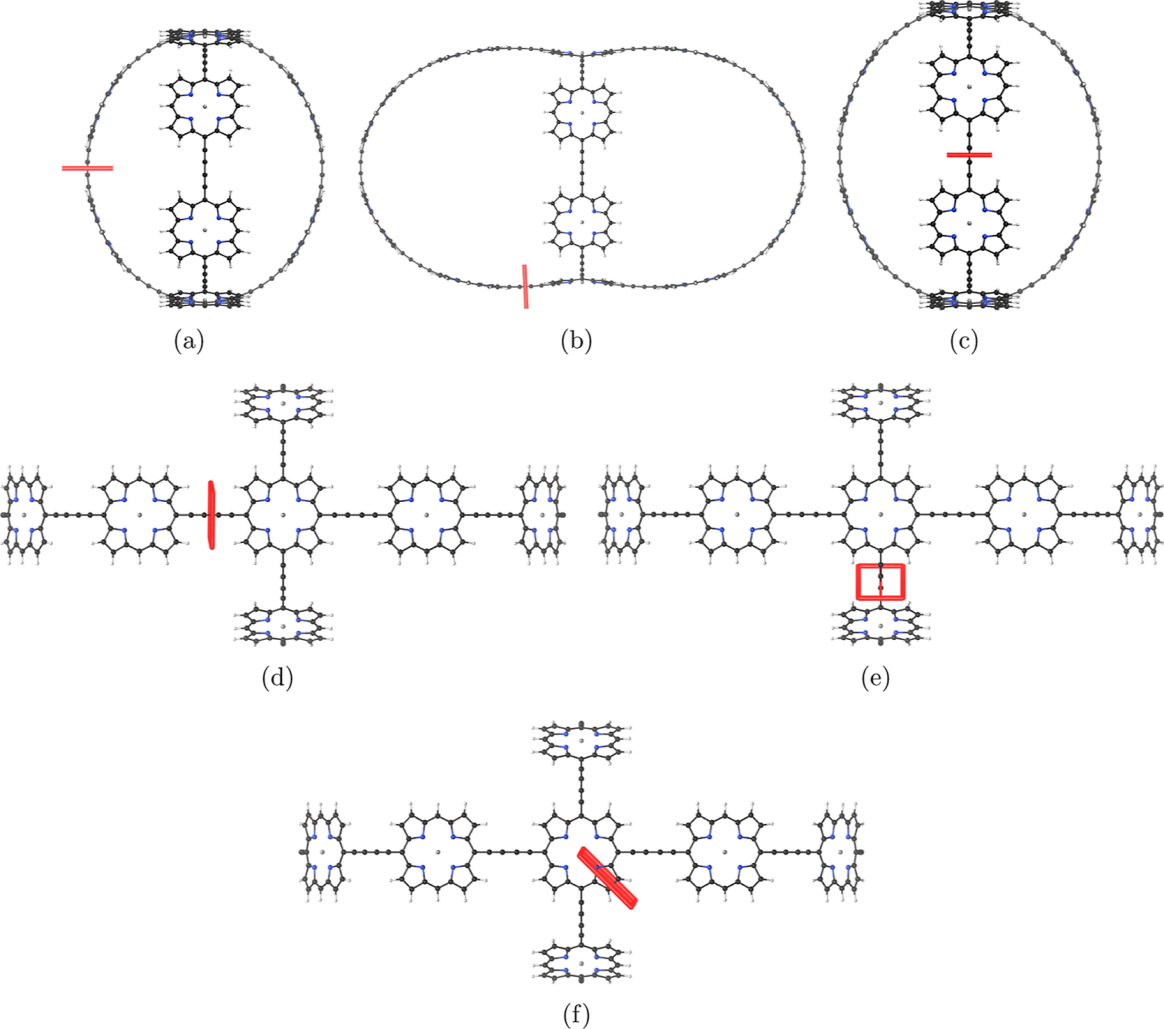
Position of the integration planes 3A-3F in molecule **3**. The integrated MICD strengths are reported in Table 3. The magnetic field vector is parallel to the integration planes shown in red.

**Figure 8:**
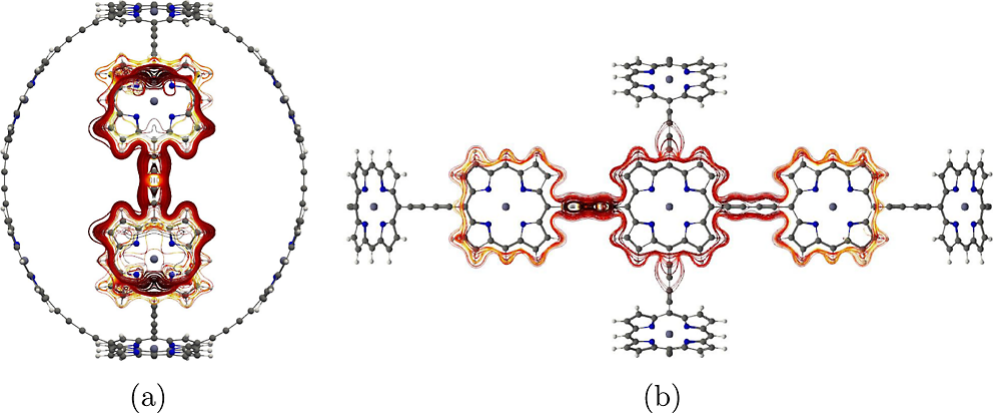
MICD flux in molecule **3**. The magnetic field vector points toward the viewer. More MICD pictures are reported as the Supporting Information.

The strength of the MICD pathways of molecule **3** in Table 3 were obtained by using the integration planes shown in Figure 7. It is nonaromatic, sustaining weak global ring currents of −1.8 and −0.3 nA·T^–1^ in the six-membered and ten-membered nanorings. The net ring current passing through plane 3C vanishes for symmetry reasons. The ring-current strength of the porphyrins is 20.9 nA·T^–1^ showing that the porphyrin units are slightly less aromatic than free-base porphyrin.^75^ The atomic ring current around the Zn atom is 38.1 nA·T^–1^. The MICD pathways of **3** are shown in Figure 8.

**Table 3:** Strength of the Diatropic and Paratropic Contributions (in nA·T^–1^) to the Net MICD Strength Passing Different Planes in Molecule **3** and Its Dication^a^

plane	diatropic	paratropic	net
Neutral			
3A	6.73	–8.53	–1.79
3B	7.46	–7.74	–0.28
3C	7.61	–7.61	0.00
3D	7.49	–7.49	0.00
3E	6.23	–6.23	0.00
3F	34.96	–14.04	20.92
Dication			
3A	22.35	–0.28	22.06
3B	37.11	–0.07	37.04
3C	7.68	–7.68	0.00
3D	7.57	–7.57	0.00
3E	6.28	–6.28	0.00
3F	33.09	–14.73	18.35

a The net current strength vanishes for plane 3C due to symmetry, whereas they vanish in planes 3D and 3E because the current-density vortex is localized as shown in Figure 8.

**Figure 9:**
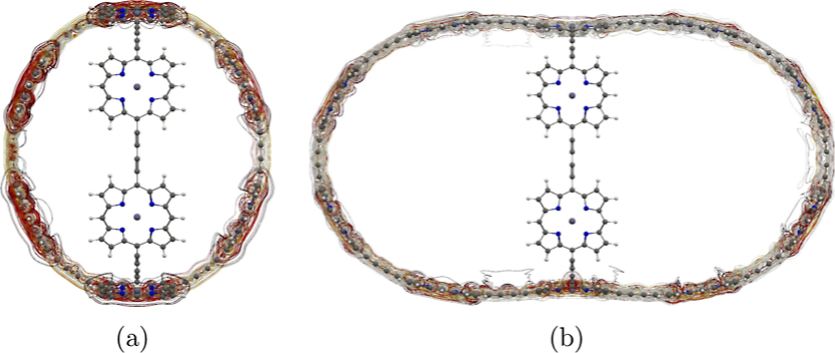
Global aromatic MICD pathways in the dication of molecule **3**. The magnetic field vector points toward the viewer.

The dication of **3** is globally aromatic, sustaining strong diatropic ring currents of 22.1 and 37.0 nA·T^–1^ in the shorter and longer nanorings, respectively. The porphyrin units sustain a local ring current of 18.4 nA·T^–1^, which is slightly less than that for neutral **3**. Oxidation does not affect the atomic ring current of Zn. The strong global ring currents are seen in Figure 9.

### 3.4 Molecule **4**

**Figure 10:**
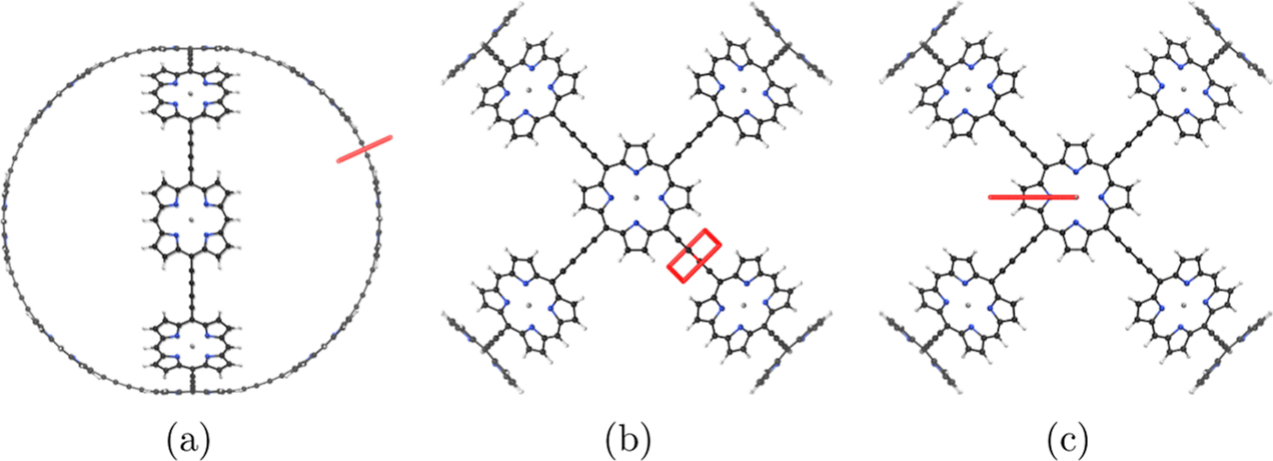
Position of the integration planes 4A–4C in molecule **4**. The integrated MICD strengths are reported in Table 4. The magnetic field vector is parallel to the integration planes shown in red.

**Figure 11:**
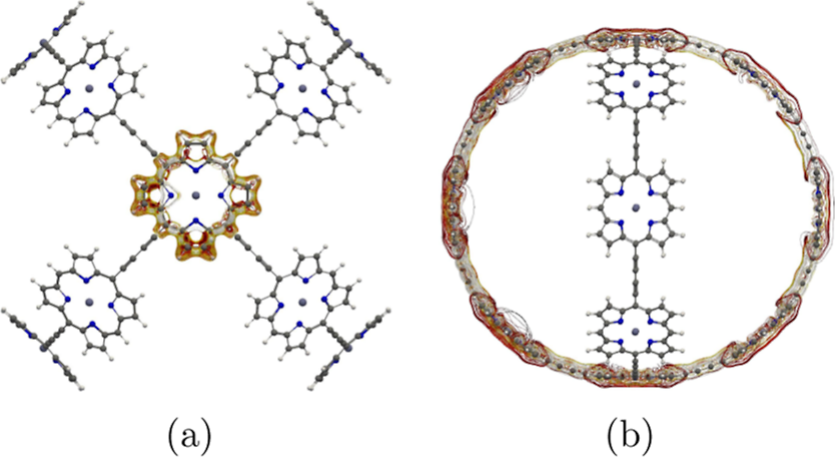
MICD flux in (a) molecule **4** and (b) its dication. The magnetic field vector points toward the viewer. More MICD pictures are reported as the Supporting Information.

The ring-current strengths of molecule **4** in Table 4 were obtained by integrating the MICD passing through the indicated planes in Figure 10. Molecule **4** is non-aromatic since it sustains a very weak ring current of −0.5 nA·T^–1^ around the nanoring. The ring current of the porphyrin moiety is 21.4 nA·T^–1^, excluding the Zn atomic current of 42.0 nA·T^–1^. The MICD of **4** is shown in Figure 11a. The dication of **4** is aromatic, sustaining a global ring current shown in Figure 11b, whose strength is 32.1 nA·T^–1^.

**Table 4:** Strength of the Diatropic and Paratropic Contributions (in nA·T^–1^) to the Net MICD Strength Passing Different Planes in Molecule **4** and Its Dication^a^

plane	diatropic	paratropic	net
Neutral			
4A	7.39	–7.87	–0.48
4B	7.15	–7.15	0.00
4C	35.7	–14.35	21.35
Dication			
4A	32.25	–0.12	32.13
4B	7.22	–7.22	0.00
4C	33.43	–15.04	18.38

a The net current strength vanishes for plane 4B because it is a local current-density vortex.

### 3.5 Molecule **5**

**Figure 12:**
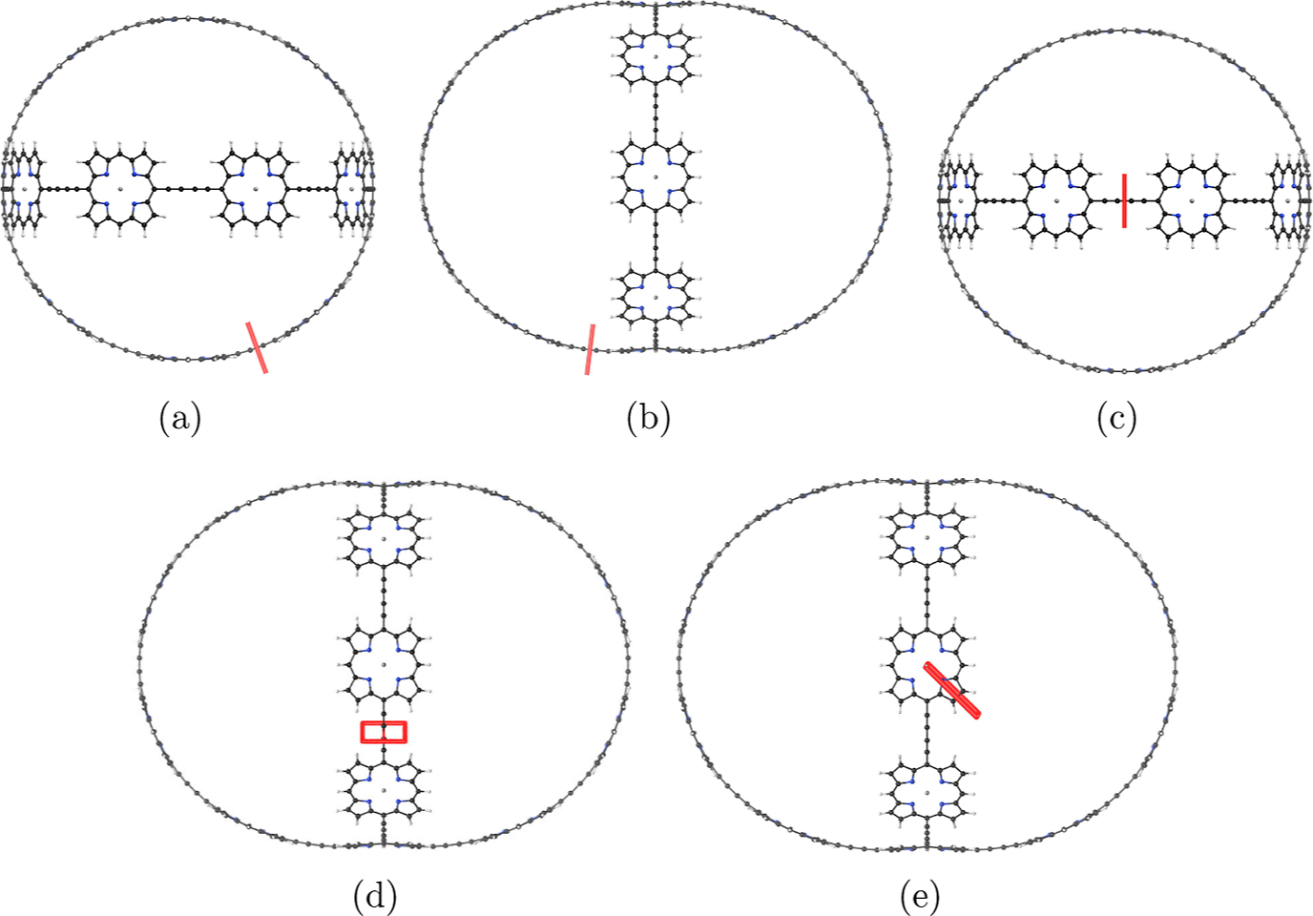
Position of the integration planes 5 A–5E in molecule **5**. The integrated current strengths are reported in Table 5. The magnetic field vector is parallel to the integration planes shown in red.

**Figure 13:**
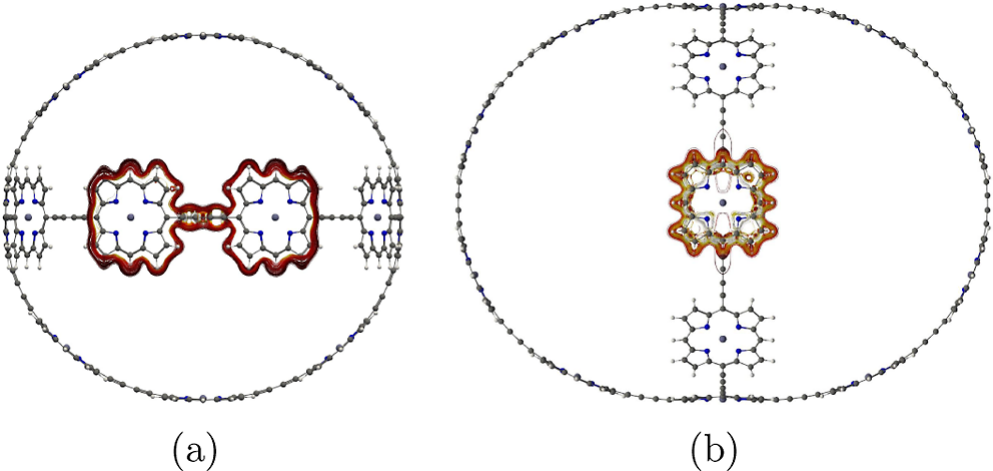
MICD flux in molecule **5**. The magnetic field vector points toward the viewer. More MICD pictures are reported as the Supporting Information.

The strengths of the MICD pathways of **5** were obtained by integrating the MICD passing through the planes shown in Figure 12. The strength of the global ring current in the shorter and the longer nanorings is −0.1 and −0.4 nA·T^–1^, respectively, suggesting that **5** is globally nonaromatic. The net ring-current strengths through plane 5C vanishes for symmetry reasons. The local ring current of the Zn porphyrins is 24.7 nA·T^–1^, which is slightly weaker than in free-base porphyrin. The atomic ring current of Zn is 36.4 nA·T^–1^. The diatropic and paratropic contributions to the ring-current strengths are given in Table 5. The studied pathways of the MICD are shown in Figure 13. Since the HOMO–LUMO gap of its dication is very small, we did not study its magnetic response.

**Table 5:** Strength of the Diatropic and Paratropic Contributions to the Net Strength of the Current Density (in nA·T^–1^) Passing Different Planes of Molecule **5**^a^

plane	diatropic	paratropic	net
5A	7.42	–7.80	–0.38
5B	7.54	–7.65	–0.11
5C	7.56	–7.56	0.00
5D	7.07	–7.07	0.00
5E	37.71	–13.05	24.66

a The net current strength vanishes for plane 5C due to symmetry as seen in Figure 13 and for 5D because it is a local current-density vortex.

### 3.6 Molecule **6**

**Figure 14:**
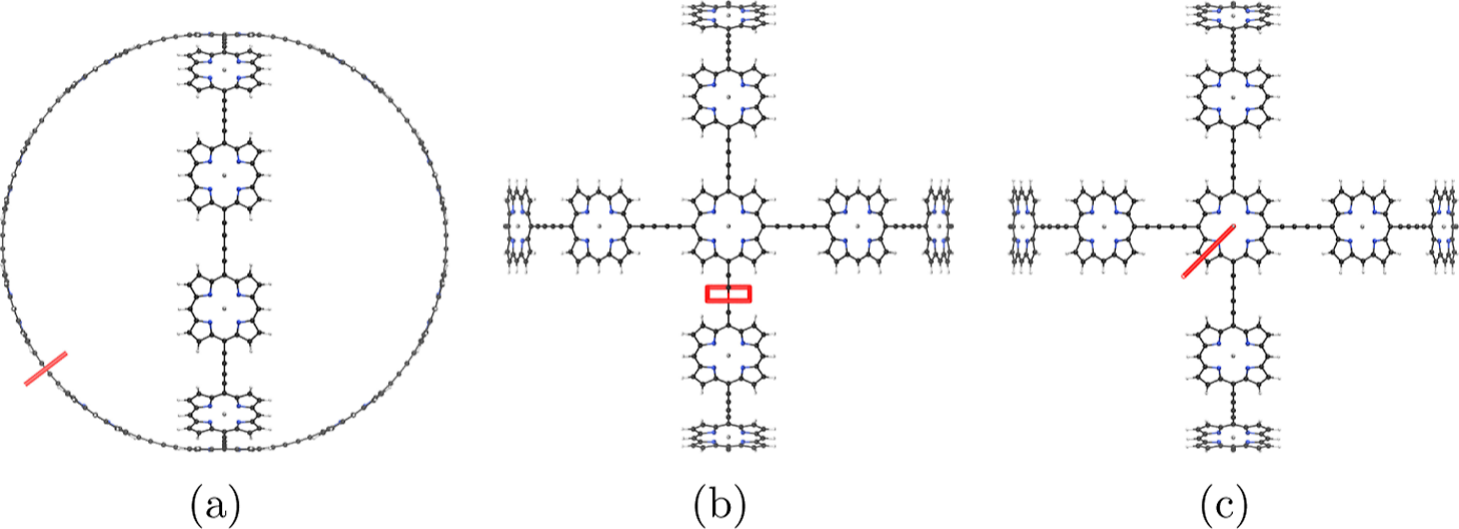
Position of the integration planes 6A–6C in molecule **6**. The integrated current strengths are reported in Table 6. The magnetic field vector is parallel to the integration planes shown in red.

**Figure 15:**
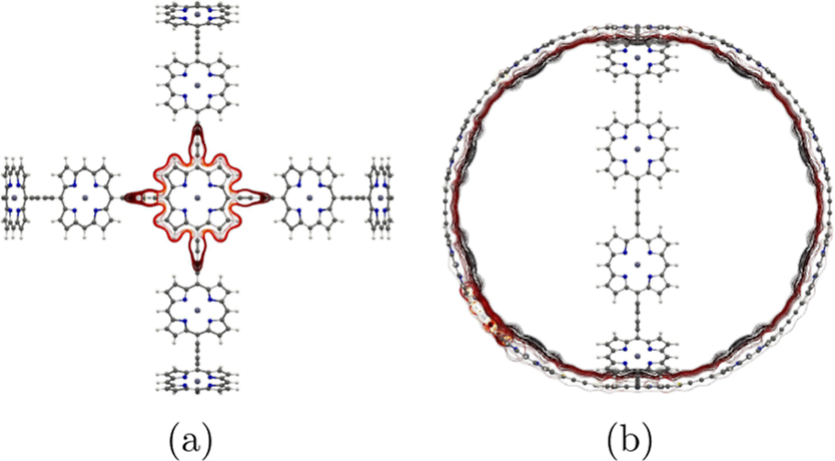
Current-density flux in (a) molecule **6** and (b) its dication. The magnetic field vector points toward the viewer. More MICD pictures are reported as the Supporting Information.

The integration planes for calculating the strength of the MICD flux along selected chemical bonds are shown in Figure 14. The corresponding MICD pathways are shown in Figure 15a. The obtained ring-current strengths in Table 6 shows that the molecule is globally nonaromatic with aromatic porphyrin units, whose ring-current strength is 21.4 nA·T^–1^. The Zn atoms sustain an atomic ring current of 40.8 nA·T^–1^.

**Table 6:** Strength of the Diatropic and Paratropic Contributions to the Net Strength of the Current Density (in nA·T^–1^) Passing Different Planes of Molecule **6**^a^

plane	diatropic	paratropic	net
Neutral			
6A	7.56	–7.68	–0.12
6B	7.27	–7.27	0.00
6C	35.58	–14.21	21.37
Dication			
6A	32.08	–0.13	30.95
6B	7.35	–7.35	0.00
6C	33.97	–14.71	19.26

a The net current strength vanishes for plane 6B because it is a local current-density vortex.

Similar calculations on the dication of **6** show that it is globally aromatic sustaining a ring current of 31.0 nA·T^–1^ (see Table 6). Its global ring current is shown in Figure 15b. The local ring currents of the porphyrins are 19.3 nA·T^–1^, which is 2.1 nA·T^–1^ weaker than for neutral **6** and 8 nA·T^–1^ weaker than for Zn porphyrin.

### 3.7 Molecule **7**

**Figure 16:**
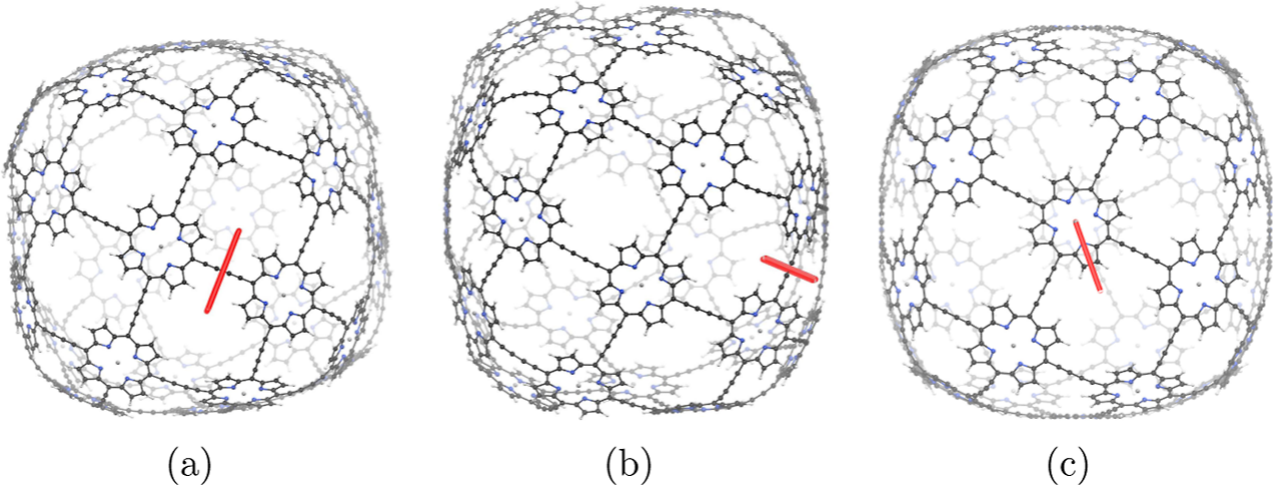
Position of the integration planes 7A–7C in molecule **7**. The integrated current strengths are reported in Table 7. The magnetic field vector is parallel to the integration planes shown in red.

**Figure 17:**
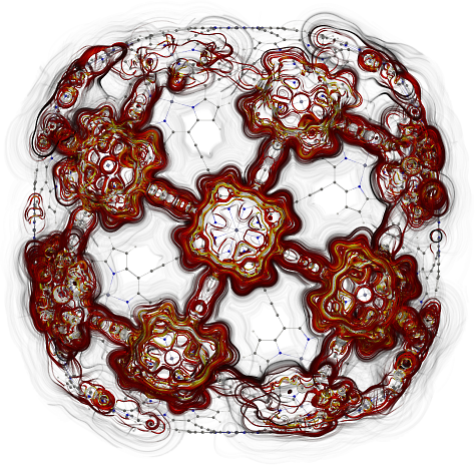
MICD flux in molecule **7**. The magnetic field vector points toward the viewer. More MICD pictures are reported as the Supporting Information.

The MICD was calculated with the magnetic field oriented in the viewing direction in Figure 16. The integrated ring-current strengths in Table 7 are the ones passing through the planes indicated with the red bars in Figure 16. The current density in Figure 17 shows that **7** sustains only local current-density vortices in the porphyrins and in the butadiyne linkers. The ring current of the porphyrins is 20.7 nA·T^–1^, which is 7 nA·T^–1^ weaker than in Zn porphyrin. The diatropic atomic current around Zn is 40.8 nA·T^–1^. The net ring current in the butadiyne linkers vanishes because the current density circulates around it and does not pass from a porphyrin to the next, implying that **7** is globally nonaromatic. Cations of **7** were not investigated because their HOMO–LUMO gap is very small.

**Table 7:** Strength of the Diatropic and Paratropic Contributions to the Net Strength of the Current Density (in nA·T^–1^) Passing Different Planes of Molecule **7**

plane	diatropic	paratropic	net
7A	7.62	–7.59	0.03
7B	7.00	–6.88	0.13
7C	34.88	–14.19	20.69

## 4 Conclusions

Molecular structures of six crossed-belt Zn-porphyrin nanostructures and one Zn-porphyrin nanoball have been constructed using polyhedral graphs with vertices of the fourth degree. The molecular structures were fully optimized at the density functional theory (DFT) level using the BP86 functional. The Cartesian coordinates of the optimized molecular structures are given as the Supporting Information. The studied crossed-belt structures are similar to the recently synthesized molecule by Anderson et al.^32^ The porphyrin nanostructures consist of Zn-porphyrin units connected via butadiyne linkers. Molecule **7** (b-P30) has a molecular structure consisting of 30 connected Zn porphyrins that form a ball-shaped Zn-porphyrin nanostructure.

The MICD of the Zn-porphyrin nanostructures was studied with our GIMIC method at the DFT level using the B3LYP functional. The current densities were visually studied with the Paraview program.

The strength of the current density passing selected chemical bonds was calculated to assess whether the molecules are globally aromatic or not. The Zn-porphyrin units are found to be locally aromatic, sustaining strong ring currents around the porphyrins, whereas none of the porphyrin nanostructures is globally aromatic. Similar calculations on the dications showed that the dications of **1**, **3**, **4**, and **6** are globally aromatic with strong ring currents, whereas the HOMO–LUMO gap of the dications of **2**, **5**, and **7** is very small, rendering MICD calculations on the dications problematic.

The orbital energies show that the neutral molecules have closed shells with dense energy levels below the HOMO. Thus, oxidation yields cations with at least one hole in the valence band. When the dications are exposed to an external magnetic field, they sustain a global ring current due to the hole in the valence band. The dense energy spectrum also implies that it does not matter whether the dication has a singlet or a triplet ground state because both states have a hole in the valence band.

Since many of the studied molecules have a very small energy splitting between the highest occupied orbitals, the HOMO–LUMO gap of charged species is small rendering DFT and MICD calculations on the dications of **2**, **5**, and **7** difficult. The orbital energy levels of the studied molecules suggest that molecules with a higher charge than +2 are expected to have a very small HOMO–LUMO gap. Lots of electrons have to be removed from the valence band to obtain cations with a large HOMO–LUMO gap.

We show that the magnetic response of very large porphyrinoid nanostructures can be successfully studied at the DFT level with our GIMIC approach. Further studies of porphyrin nanostructures will be performed to explore this active field of chemistry.

## Supporting Information Available

Cartesian coordinates of the molecular structures and complementary current-density pictures (PDF)
