# Supplementary material for: Magnetically Induced Current Densities in π-Conjugated Porphyrin Nanoballs
Source: J Phys Chem A. 2022 Oct 21;126(43):7864–73. doi: 10.1021/acs.jpca.2c04856 (PMC9639160; doi:10.1021/acs.jpca.2c04856)
Supplement: Supplementary file 1 — jp2c04856_si_001.pdf [file jp2c04856_si_001.pdf]

Magnetically Induced Current Densities in π-Conjugated Porphyrin Nanoballs

Atif Mahmood,* Maria Dimitrova,* Lukas N. Wirz,* and Dage Sundholm*

Department of Chemistry, University of Helsinki, P.O. Box 55, A. I. Virtasen aukio 1, FIN-00014 University of Helsinki, Finland

E-mail: atif.mahmood@helsinki.fi; maria.dimitrova@helsinki.fi; lnwirz@chem.helsinki.fi; dage.sundholm@helsinki.fi

Complementary current-density pictures

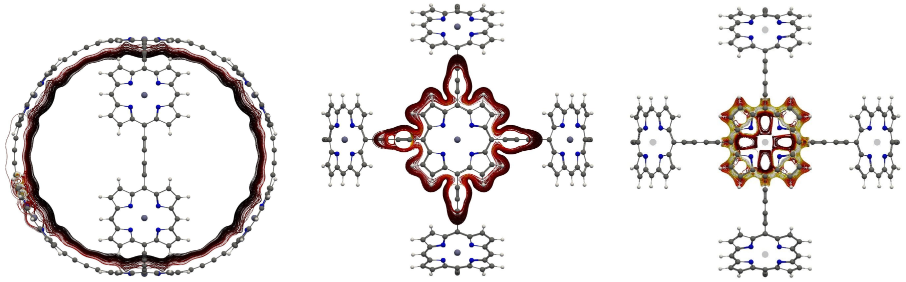

(a) (b) (c)

Figure S1: The MICD flux in molecule **1**.

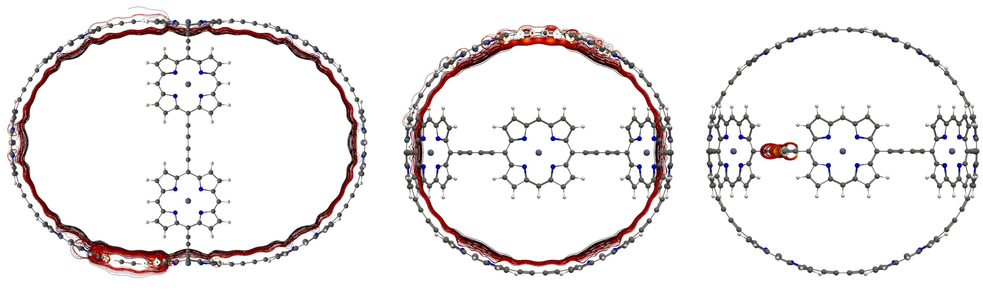

(a) (b) (c)

Figure S2: The MICD flux in different parts of molecule **2**.

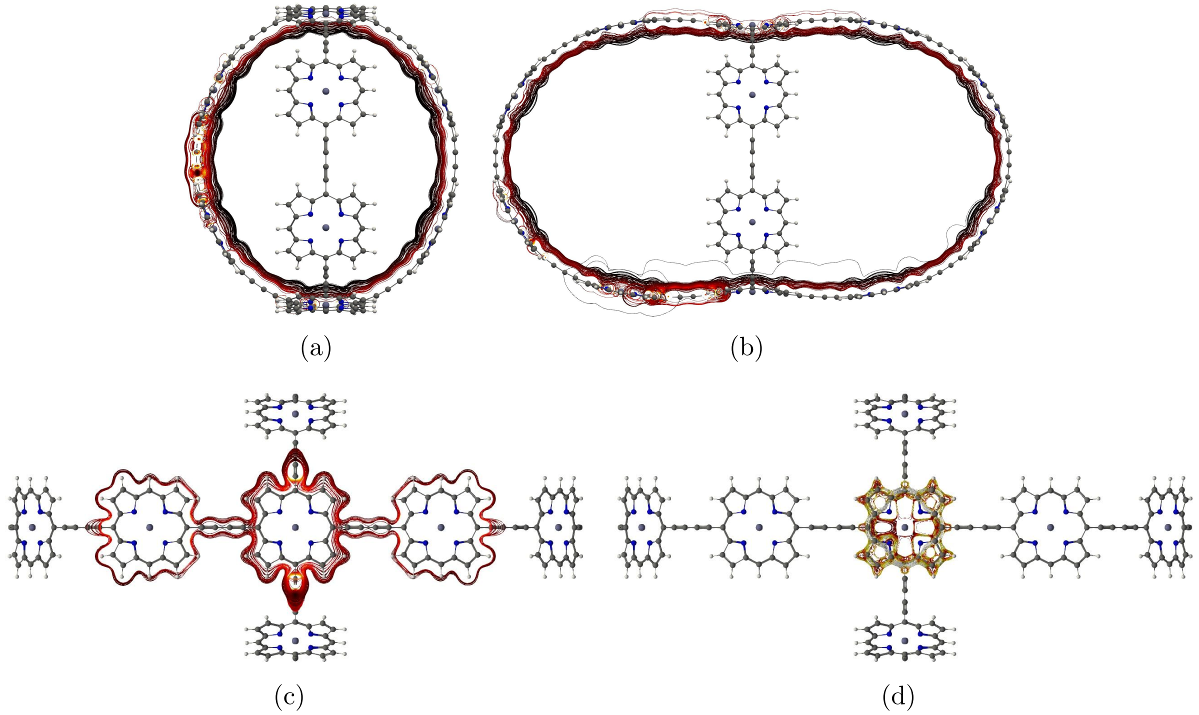

(a) (b) (c) (d)

Figure S3: The MICD fluxes in molecule **3**.

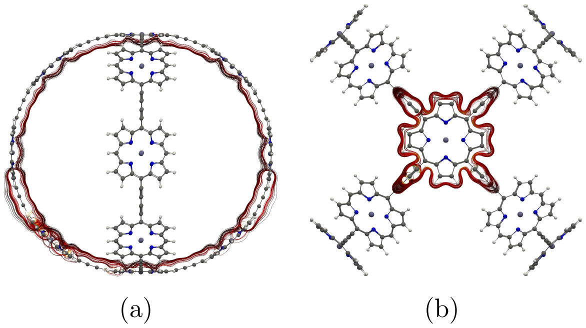

(a) (b)

Figure S4: The MICD flux in molecule **4** and its dication.

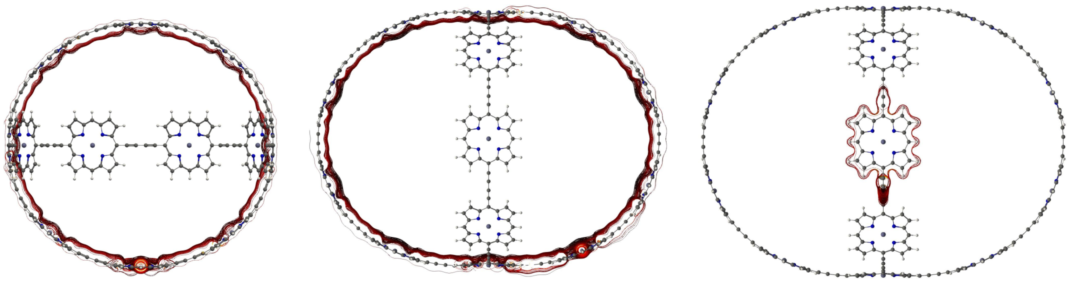

(a) (b) (c)

Figure S5: The current-density flux in molecule **5**.

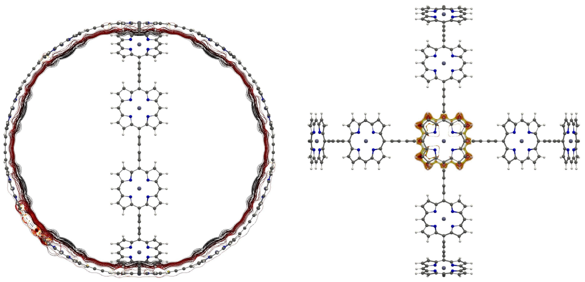

(a) (b)

Figure S6: The current-density flux in molecule **6** and its dication.

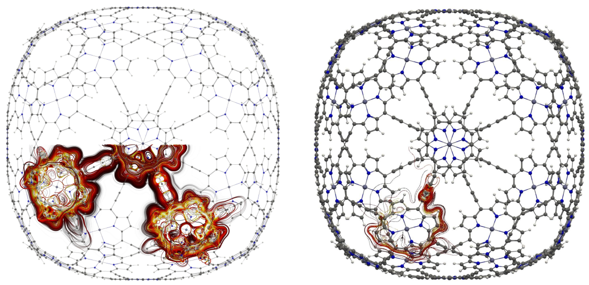

(a) (b)

Figure S7: The current-density flux in molecule **7**.

Molecular structures

Molecule 1

```
394
Energy =
```

Zn	-0.0000000	-0.0000000	12.1781534
C	3.5117847	2.5381433	12.0522946
C	2.5381433	3.5117847	12.0522946
C	1.2670370	2.8255102	12.1109466
C	2.8255102	1.2670370	12.1109466
N	1.4616903	1.4616903	12.1666240
C	3.4790428	0.0000000	12.0464488
C	2.8255102	-1.2670370	12.1109466
C	3.5117847	-2.5381433	12.0522946
C	2.5381433	-3.5117847	12.0522946
C	1.2670370	-2.8255102	12.1109466
N	1.4616903	-1.4616903	12.1666240
C	0.0000000	-3.4790428	12.0464488
C	-1.2670370	-2.8255102	12.1109466
C	-2.5381433	-3.5117847	12.0522946
C	-3.5117847	-2.5381433	12.0522946
C	-2.8255102	-1.2670370	12.1109466
N	-1.4616903	-1.4616903	12.1666240
C	-3.4790428	-0.0000000	12.0464488
C	-2.8255102	1.2670370	12.1109466
C	-3.5117847	2.5381433	12.0522946
C	-2.5381433	3.5117847	12.0522946
C	-1.2670370	2.8255102	12.1109466
N	-1.4616903	1.4616903	12.1666240
C	-0.0000000	3.4790428	12.0464488
H	4.5989437	2.6626450	12.0104600
H	2.6626450	4.5989437	12.0104600
H	4.5989437	-2.6626450	12.0104600
H	2.6626450	-4.5989437	12.0104600
H	-2.6626450	-4.5989437	12.0104600
H	-4.5989437	-2.6626450	12.0104600
H	-4.5989437	2.6626450	12.0104600
H	-2.6626450	4.5989437	12.0104600
Zn	11.4751516	0.0000000	6.3909049
C	12.8281081	-3.5051320	4.2594678
C	13.1827174	-2.5460067	3.3366568
C	12.8203258	-1.2650847	3.9102388
C	12.2232338	-2.8072251	5.3764502
N	12.2478847	-1.4433692	5.1444375
C	11.6260589	-3.4262523	6.4849702
C	10.8934788	-2.8074854	7.5087028
C	10.1563581	-3.5053044	8.5434510
C	9.4838624	-2.5462300	9.2678774
C	9.8398373	-1.2651459	8.6900739
N	10.6960754	-1.4434164	7.6330514
C	9.3632529	0.0000000	9.1565820
C	9.8398373	1.2651459	8.6900739
C	9.4838624	2.5462300	9.2678774
C	10.1563581	3.5053044	8.5434510

C	10.8934788	2.8074854	7.5087028
N	10.6960754	1.4434164	7.6330514
C	11.6260589	3.4262523	6.4849702
C	12.2232338	2.8072251	5.3764502
C	12.8281081	3.5051320	4.2594678
C	13.1827174	2.5460067	3.3366568
C	12.8203258	1.2650847	3.9102388
N	12.2478847	1.4433692	5.1444375
C	13.0291085	-0.0000000	3.2765618
H	12.9491416	-4.5924257	4.1852579
H	13.6559113	-2.6824849	2.3582000
H	10.1427640	-4.5925954	8.6847476
H	8.8134109	-2.6827312	10.1233146
H	8.8134109	2.6827312	10.1233146
H	10.1427640	4.5925954	8.6847476
H	12.9491416	4.5924257	4.1852579
H	13.6559113	2.6824849	2.3582000
Zn	-11.4751516	0.0000000	6.3909049
C	-12.8281081	3.5051320	4.2594678
C	-13.1827174	2.5460067	3.3366568
C	-12.8203258	1.2650847	3.9102388
C	-12.2232338	2.8072251	5.3764502
N	-12.2478847	1.4433692	5.1444375
C	-11.6260589	3.4262523	6.4849702
C	-10.8934788	2.8074854	7.5087028
C	-10.1563581	3.5053044	8.5434510
C	-9.4838624	2.5462300	9.2678774
C	-9.8398373	1.2651459	8.6900739
N	-10.6960754	1.4434164	7.6330514
C	-9.3632529	-0.0000000	9.1565820
C	-9.8398373	-1.2651459	8.6900739
C	-9.4838624	-2.5462300	9.2678774
C	-10.1563581	-3.5053044	8.5434510
C	-10.8934788	-2.8074854	7.5087028
N	-10.6960754	-1.4434164	7.6330514
C	-11.6260589	-3.4262523	6.4849702
C	-12.2232338	-2.8072251	5.3764502
C	-12.8281081	-3.5051320	4.2594678
C	-13.1827174	-2.5460067	3.3366568
C	-12.8203258	-1.2650847	3.9102388
N	-12.2478847	-1.4433692	5.1444375
C	-13.0291085	0.0000000	3.2765618
H	-12.9491416	4.5924257	4.1852579
H	-13.6559113	2.6824849	2.3582000
H	-10.1427640	4.5925954	8.6847476
H	-8.8134109	2.6827312	10.1233146
H	-8.8134109	-2.6827312	10.1233146
H	-10.1427640	-4.5925954	8.6847476
H	-12.9491416	-4.5924257	4.1852579

H	-13.6559113	-2.6824849	2.3582000
Zn	11.4751516	0.0000000	-6.3909049
C	13.1827174	-2.5460067	-3.3366568
C	12.8281081	-3.5051320	-4.2594678
C	12.2232338	-2.8072251	-5.3764502
C	12.8203258	-1.2650847	-3.9102388
N	12.2478847	-1.4433692	-5.1444375
C	13.0291085	-0.0000000	-3.2765618
C	12.8203258	1.2650847	-3.9102388
C	13.1827174	2.5460067	-3.3366568
C	12.8281081	3.5051320	-4.2594678
C	12.2232338	2.8072251	-5.3764502
N	12.2478847	1.4433692	-5.1444375
C	11.6260589	3.4262523	-6.4849702
C	10.8934788	2.8074854	-7.5087028
C	10.1563581	3.5053044	-8.5434510
C	9.4838624	2.5462300	-9.2678774
C	9.8398373	1.2651459	-8.6900739
N	10.6960754	1.4434164	-7.6330514
C	9.3632529	0.0000000	-9.1565820
C	9.8398373	-1.2651459	-8.6900739
C	9.4838624	-2.5462300	-9.2678774
C	10.1563581	-3.5053044	-8.5434510
C	10.8934788	-2.8074854	-7.5087028
N	10.6960754	-1.4434164	-7.6330514
C	11.6260589	-3.4262523	-6.4849702
H	13.6559113	-2.6824849	-2.3582000
H	12.9491416	-4.5924257	-4.1852579
H	13.6559113	2.6824849	-2.3582000
H	12.9491416	4.5924257	-4.1852579
H	10.1427640	4.5925954	-8.6847476
H	8.8134109	2.6827312	-10.1233146
H	8.8134109	-2.6827312	-10.1233146
H	10.1427640	-4.5925954	-8.6847476
Zn	-11.4751516	0.0000000	-6.3909049
C	-13.1827174	2.5460067	-3.3366568
C	-12.8281081	3.5051320	-4.2594678
C	-12.2232338	2.8072251	-5.3764502
C	-12.8203258	1.2650847	-3.9102388
N	-12.2478847	1.4433692	-5.1444375
C	-13.0291085	0.0000000	-3.2765618
C	-12.8203258	-1.2650847	-3.9102388
C	-13.1827174	-2.5460067	-3.3366568
C	-12.8281081	-3.5051320	-4.2594678
C	-12.2232338	-2.8072251	-5.3764502
N	-12.2478847	-1.4433692	-5.1444375
C	-11.6260589	-3.4262523	-6.4849702
C	-10.8934788	-2.8074854	-7.5087028
C	-10.1563581	-3.5053044	-8.5434510

C	-9.4838624	-2.5462300	-9.2678774
C	-9.8398373	-1.2651459	-8.6900739
N	-10.6960754	-1.4434164	-7.6330514
C	-9.3632529	-0.0000000	-9.1565820
C	-9.8398373	1.2651459	-8.6900739
C	-9.4838624	2.5462300	-9.2678774
C	-10.1563581	3.5053044	-8.5434510
C	-10.8934788	2.8074854	-7.5087028
N	-10.6960754	1.4434164	-7.6330514
C	-11.6260589	3.4262523	-6.4849702
H	-13.6559113	2.6824849	-2.3582000
H	-12.9491416	4.5924257	-4.1852579
H	-13.6559113	-2.6824849	-2.3582000
H	-12.9491416	-4.5924257	-4.1852579
H	-10.1427640	-4.5925954	-8.6847476
H	-8.8134109	-2.6827312	-10.1233146
H	-8.8134109	2.6827312	-10.1233146
H	-10.1427640	4.5925954	-8.6847476
Zn	-0.0000000	0.0000000	-12.1781534
C	3.5117847	-2.5381433	-12.0522946
C	2.5381433	-3.5117847	-12.0522946
C	1.2670370	-2.8255102	-12.1109466
C	2.8255102	-1.2670370	-12.1109466
N	1.4616903	-1.4616903	-12.1666240
C	3.4790428	0.0000000	-12.0464488
C	2.8255102	1.2670370	-12.1109466
C	3.5117847	2.5381433	-12.0522946
C	2.5381433	3.5117847	-12.0522946
C	1.2670370	2.8255102	-12.1109466
N	1.4616903	1.4616903	-12.1666240
C	-0.0000000	3.4790428	-12.0464488
C	-1.2670370	2.8255102	-12.1109466
C	-2.5381433	3.5117847	-12.0522946
C	-3.5117847	2.5381433	-12.0522946
C	-2.8255102	1.2670370	-12.1109466
N	-1.4616903	1.4616903	-12.1666240
C	-3.4790428	-0.0000000	-12.0464488
C	-2.8255102	-1.2670370	-12.1109466
C	-3.5117847	-2.5381433	-12.0522946
C	-2.5381433	-3.5117847	-12.0522946
C	-1.2670370	-2.8255102	-12.1109466
N	-1.4616903	-1.4616903	-12.1666240
C	0.0000000	-3.4790428	-12.0464488
H	4.5989437	-2.6626450	-12.0104600
H	2.6626450	-4.5989437	-12.0104600
H	4.5989437	2.6626450	-12.0104600
H	2.6626450	4.5989437	-12.0104600
H	-2.6626450	4.5989437	-12.0104600
H	-4.5989437	2.6626450	-12.0104600

H	-4.5989437	-2.6626450	-12.0104600
H	-2.6626450	-4.5989437	-12.0104600
Zn	0.0000000	11.4751516	6.3909049
C	3.5051320	12.8281081	4.2594678
C	2.5460067	13.1827174	3.3366568
C	1.2650847	12.8203258	3.9102388
C	2.8072251	12.2232338	5.3764502
N	1.4433692	12.2478847	5.1444375
C	3.4262523	11.6260589	6.4849702
C	2.8074854	10.8934788	7.5087028
C	3.5053044	10.1563581	8.5434510
C	2.5462300	9.4838624	9.2678774
C	1.2651459	9.8398373	8.6900739
N	1.4434164	10.6960754	7.6330514
C	-0.0000000	9.3632529	9.1565820
C	-1.2651459	9.8398373	8.6900739
C	-2.5462300	9.4838624	9.2678774
C	-3.5053044	10.1563581	8.5434510
C	-2.8074854	10.8934788	7.5087028
N	-1.4434164	10.6960754	7.6330514
C	-3.4262523	11.6260589	6.4849702
C	-2.8072251	12.2232338	5.3764502
C	-3.5051320	12.8281081	4.2594678
C	-2.5460067	13.1827174	3.3366568
C	-1.2650847	12.8203258	3.9102388
N	-1.4433692	12.2478847	5.1444375
C	0.0000000	13.0291085	3.2765618
H	4.5924257	12.9491416	4.1852579
H	2.6824849	13.6559113	2.3582000
H	4.5925954	10.1427640	8.6847476
H	2.6827312	8.8134109	10.1233146
H	-2.6827312	8.8134109	10.1233146
H	-4.5925954	10.1427640	8.6847476
H	-4.5924257	12.9491416	4.1852579
H	-2.6824849	13.6559113	2.3582000
Zn	0.0000000	-11.4751516	6.3909049
C	2.5460067	-13.1827174	3.3366568
C	3.5051320	-12.8281081	4.2594678
C	2.8072251	-12.2232338	5.3764502
C	1.2650847	-12.8203258	3.9102388
N	1.4433692	-12.2478847	5.1444375
C	-0.0000000	-13.0291085	3.2765618
C	-1.2650847	-12.8203258	3.9102388
C	-2.5460067	-13.1827174	3.3366568
C	-3.5051320	-12.8281081	4.2594678
C	-2.8072251	-12.2232338	5.3764502
N	-1.4433692	-12.2478847	5.1444375
C	-3.4262523	-11.6260589	6.4849702
C	-2.8074854	-10.8934788	7.5087028

C	-3.5053044	-10.1563581	8.5434510
C	-2.5462300	-9.4838624	9.2678774
C	-1.2651459	-9.8398373	8.6900739
N	-1.4434164	-10.6960754	7.6330514
C	0.0000000	-9.3632529	9.1565820
C	1.2651459	-9.8398373	8.6900739
C	2.5462300	-9.4838624	9.2678774
C	3.5053044	-10.1563581	8.5434510
C	2.8074854	-10.8934788	7.5087028
N	1.4434164	-10.6960754	7.6330514
C	3.4262523	-11.6260589	6.4849702
H	2.6824849	-13.6559113	2.3582000
H	4.5924257	-12.9491416	4.1852579
H	-2.6824849	-13.6559113	2.3582000
H	-4.5924257	-12.9491416	4.1852579
H	-4.5925954	-10.1427640	8.6847476
H	-2.6827312	-8.8134109	10.1233146
H	2.6827312	-8.8134109	10.1233146
H	4.5925954	-10.1427640	8.6847476
Zn	0.0000000	11.4751516	-6.3909049
C	2.5460067	13.1827174	-3.3366568
C	3.5051320	12.8281081	-4.2594678
C	2.8072251	12.2232338	-5.3764502
C	1.2650847	12.8203258	-3.9102388
N	1.4433692	12.2478847	-5.1444375
C	0.0000000	13.0291085	-3.2765618
C	-1.2650847	12.8203258	-3.9102388
C	-2.5460067	13.1827174	-3.3366568
C	-3.5051320	12.8281081	-4.2594678
C	-2.8072251	12.2232338	-5.3764502
N	-1.4433692	12.2478847	-5.1444375
C	-3.4262523	11.6260589	-6.4849702
C	-2.8074854	10.8934788	-7.5087028
C	-3.5053044	10.1563581	-8.5434510
C	-2.5462300	9.4838624	-9.2678774
C	-1.2651459	9.8398373	-8.6900739
N	-1.4434164	10.6960754	-7.6330514
C	-0.0000000	9.3632529	-9.1565820
C	1.2651459	9.8398373	-8.6900739
C	2.5462300	9.4838624	-9.2678774
C	3.5053044	10.1563581	-8.5434510
C	2.8074854	10.8934788	-7.5087028
N	1.4434164	10.6960754	-7.6330514
C	3.4262523	11.6260589	-6.4849702
H	2.6824849	13.6559113	-2.3582000
H	4.5924257	12.9491416	-4.1852579
H	-2.6824849	13.6559113	-2.3582000
H	-4.5924257	12.9491416	-4.1852579
H	-4.5925954	10.1427640	-8.6847476

H	-2.6827312	8.8134109	-10.1233146
H	2.6827312	8.8134109	-10.1233146
H	4.5925954	10.1427640	-8.6847476
Zn	0.0000000	-11.4751516	-6.3909049
C	3.5051320	-12.8281081	-4.2594678
C	2.5460067	-13.1827174	-3.3366568
C	1.2650847	-12.8203258	-3.9102388
C	2.8072251	-12.2232338	-5.3764502
N	1.4433692	-12.2478847	-5.1444375
C	3.4262523	-11.6260589	-6.4849702
C	2.8074854	-10.8934788	-7.5087028
C	3.5053044	-10.1563581	-8.5434510
C	2.5462300	-9.4838624	-9.2678774
C	1.2651459	-9.8398373	-8.6900739
N	1.4434164	-10.6960754	-7.6330514
C	0.0000000	-9.3632529	-9.1565820
C	-1.2651459	-9.8398373	-8.6900739
C	-2.5462300	-9.4838624	-9.2678774
C	-3.5053044	-10.1563581	-8.5434510
C	-2.8074854	-10.8934788	-7.5087028
N	-1.4434164	-10.6960754	-7.6330514
C	-3.4262523	-11.6260589	-6.4849702
C	-2.8072251	-12.2232338	-5.3764502
C	-3.5051320	-12.8281081	-4.2594678
C	-2.5460067	-13.1827174	-3.3366568
C	-1.2650847	-12.8203258	-3.9102388
N	-1.4433692	-12.2478847	-5.1444375
C	-0.0000000	-13.0291085	-3.2765618
H	4.5924257	-12.9491416	-4.1852579
H	2.6824849	-13.6559113	-2.3582000
H	4.5925954	-10.1427640	-8.6847476
H	2.6827312	-8.8134109	-10.1233146
H	-2.6827312	-8.8134109	-10.1233146
H	-4.5925954	-10.1427640	-8.6847476
H	-4.5924257	-12.9491416	-4.1852579
H	-2.6824849	-13.6559113	-2.3582000
C	4.8635709	-0.0000000	11.7653690
C	6.0354994	0.0000000	11.3468132
C	7.2500697	-0.0000000	10.7538784
C	8.2966402	0.0000000	10.0797155
C	-0.0000000	-4.8635709	11.7653690
C	0.0000000	-6.0354994	11.3468132
C	0.0000000	-7.2500697	10.7538784
C	0.0000000	-8.2966402	10.0797155
C	-4.8635709	0.0000000	11.7653690
C	-6.0354994	-0.0000000	11.3468132
C	-7.2500697	-0.0000000	10.7538784
C	-8.2966402	0.0000000	10.0797155
C	0.0000000	4.8635709	11.7653690

C	0.0000000	6.0354994	11.3468132
C	0.0000000	7.2500697	10.7538784
C	0.0000000	8.2966402	10.0797155
C	13.3888791	0.0000000	1.9126601
C	13.5307179	0.0000000	0.6756645
C	13.5307179	0.0000000	-0.6756645
C	13.3888791	0.0000000	-1.9126601
C	-13.3888791	-0.0000000	1.9126601
C	-13.5307179	0.0000000	0.6756645
C	-13.5307179	0.0000000	-0.6756645
C	-13.3888791	-0.0000000	-1.9126601
C	8.2966402	0.0000000	-10.0797155
C	7.2500697	-0.0000000	-10.7538784
C	6.0354994	0.0000000	-11.3468132
C	4.8635709	-0.0000000	-11.7653690
C	-8.2966402	0.0000000	-10.0797155
C	-7.2500697	-0.0000000	-10.7538784
C	-6.0354994	-0.0000000	-11.3468132
C	-4.8635709	0.0000000	-11.7653690
C	0.0000000	4.8635709	-11.7653690
C	0.0000000	6.0354994	-11.3468132
C	0.0000000	7.2500697	-10.7538784
C	0.0000000	8.2966402	-10.0797155
C	-0.0000000	-4.8635709	-11.7653690
C	0.0000000	-6.0354994	-11.3468132
C	0.0000000	-7.2500697	-10.7538784
C	0.0000000	-8.2966402	-10.0797155
C	-0.0000000	13.3888791	1.9126601
C	0.0000000	13.5307179	0.6756645
C	0.0000000	13.5307179	-0.6756645
C	-0.0000000	13.3888791	-1.9126601
C	0.0000000	-13.3888791	1.9126601
C	0.0000000	-13.5307179	0.6756645
C	0.0000000	-13.5307179	-0.6756645
C	0.0000000	-13.3888791	-1.9126601
H	11.6832716	-4.5242554	6.5204992
H	11.6832716	4.5242554	6.5204992
H	-11.6832716	4.5242554	6.5204992
H	-11.6832716	-4.5242554	6.5204992
H	11.6832716	4.5242554	-6.5204992
H	11.6832716	-4.5242554	-6.5204992
H	-11.6832716	-4.5242554	-6.5204992
H	-11.6832716	4.5242554	-6.5204992
H	4.5242554	11.6832716	6.5204992
H	-4.5242554	11.6832716	6.5204992
H	-4.5242554	-11.6832716	6.5204992
H	4.5242554	-11.6832716	6.5204992
H	-4.5242554	11.6832716	-6.5204992
H	4.5242554	11.6832716	-6.5204992

H	4.5242554	-11.6832716	-6.5204992
H	-4.5242554	-11.6832716	-6.5204992

Molecule 2

472
Energy =

Zn	13.5147414	0.0000000	0.0000000
C	13.3141713	-3.4980759	-2.5371976
C	13.4904412	-2.5367154	-3.5081999
C	13.5823315	-1.2668885	-2.8221516
C	13.3281937	-2.8091789	-1.2665809
N	13.5031546	-1.4562105	-1.4592027
C	13.1638322	-3.4477351	0.0000000
C	13.3281937	-2.8091789	1.2665809
C	13.3141713	-3.4980759	2.5371976
C	13.4904412	-2.5367154	3.5081999
C	13.5823315	-1.2668885	2.8221516
N	13.5031546	-1.4562105	1.4592027
C	13.6450870	0.0000000	3.4785500
C	13.5823315	1.2668885	2.8221516
C	13.4904412	2.5367154	3.5081999
C	13.3141713	3.4980759	2.5371976
C	13.3281937	2.8091789	1.2665809
N	13.5031546	1.4562105	1.4592027
C	13.1638322	3.4477351	0.0000000
C	13.3281937	2.8091789	-1.2665809
C	13.3141713	3.4980759	-2.5371976
C	13.4904412	2.5367154	-3.5081999
C	13.5823315	1.2668885	-2.8221516
N	13.5031546	1.4562105	-1.4592027
C	13.6450870	0.0000000	-3.4785500
H	13.1925222	-4.5791305	-2.6637403
H	13.5363023	-2.6679473	-4.5943735
H	13.1925222	-4.5791305	2.6637403
H	13.5363023	-2.6679473	4.5943735
H	13.5363023	2.6679473	4.5943735
H	13.1925222	4.5791305	2.6637403
H	13.1925222	4.5791305	-2.6637403
H	13.5363023	2.6679473	-4.5943735
Zn	-13.5147414	0.0000000	0.0000000
C	-13.3141713	-3.4980759	2.5371976
C	-13.4904412	-2.5367154	3.5081999
C	-13.5823315	-1.2668885	2.8221516
C	-13.3281937	-2.8091789	1.2665809
N	-13.5031546	-1.4562105	1.4592027
C	-13.1638322	-3.4477351	0.0000000
C	-13.3281937	-2.8091789	-1.2665809
C	-13.3141713	-3.4980759	-2.5371976

C	-13.4904412	-2.5367154	-3.5081999
C	-13.5823315	-1.2668885	-2.8221516
N	-13.5031546	-1.4562105	-1.4592027
C	-13.6450870	0.0000000	-3.4785500
C	-13.5823315	1.2668885	-2.8221516
C	-13.4904412	2.5367154	-3.5081999
C	-13.3141713	3.4980759	-2.5371976
C	-13.3281937	2.8091789	-1.2665809
N	-13.5031546	1.4562105	-1.4592027
C	-13.1638322	3.4477351	0.0000000
C	-13.3281937	2.8091789	1.2665809
C	-13.3141713	3.4980759	2.5371976
C	-13.4904412	2.5367154	3.5081999
C	-13.5823315	1.2668885	2.8221516
N	-13.5031546	1.4562105	1.4592027
C	-13.6450870	0.0000000	3.4785500
H	-13.1925222	-4.5791305	2.6637403
H	-13.5363023	-2.6679473	4.5943735
H	-13.1925222	-4.5791305	-2.6637403
H	-13.5363023	-2.6679473	-4.5943735
H	-13.5363023	2.6679473	-4.5943735
H	-13.1925222	4.5791305	-2.6637403
H	-13.1925222	4.5791305	2.6637403
H	-13.5363023	2.6679473	4.5943735
Zn	6.5388730	10.6915004	0.0000000
C	9.6122637	9.0142615	-2.5466631
C	8.8123441	9.5930012	-3.5066558
C	7.7050356	10.2153230	-2.8085539
C	9.0027629	9.3113180	-1.2651943
N	7.8551213	10.0418310	-1.4436756
C	9.5252659	8.8971790	0.0000000
C	9.0027629	9.3113180	1.2651943
C	9.6122637	9.0142615	2.5466631
C	8.8123441	9.5930012	3.5066558
C	7.7050356	10.2153230	2.8085539
N	7.8551213	10.0418310	1.4436756
C	6.6086372	10.8329117	3.4276679
C	5.4497944	11.3243003	2.8081519
C	4.2825795	11.8246168	3.5064238
C	3.3366364	12.1079728	2.5464225
C	3.9424442	11.8040879	1.2650454
N	5.2202821	11.3365375	1.4436478
C	3.2965326	11.9702535	0.0000000
C	3.9424442	11.8040879	-1.2650454
C	3.3366364	12.1079728	-2.5464225
C	4.2825795	11.8246168	-3.5064238
C	5.4497944	11.3243003	-2.8081519
N	5.2202821	11.3365375	-1.4436478
C	6.6086372	10.8329117	-3.4276679

H	10.5370765	8.4432534	-2.6827832
H	8.9493246	9.5871471	-4.5945600
H	10.5370765	8.4432534	2.6827832
H	8.9493246	9.5871471	4.5945600
H	4.1950450	11.9302924	4.5943157
H	2.3212960	12.4957121	2.6825129
H	2.3212960	12.4957121	-2.6825129
H	4.1950450	11.9302924	-4.5943157
Zn	6.5388730	-10.6915004	0.0000000
C	8.8123441	-9.5930012	-3.5066558
C	9.6122637	-9.0142615	-2.5466631
C	9.0027629	-9.3113180	-1.2651943
C	7.7050356	-10.2153230	-2.8085539
N	7.8551213	-10.0418310	-1.4436756
C	6.6086372	-10.8329117	-3.4276679
C	5.4497944	-11.3243003	-2.8081519
C	4.2825795	-11.8246168	-3.5064238
C	3.3366364	-12.1079728	-2.5464225
C	3.9424442	-11.8040879	-1.2650454
N	5.2202821	-11.3365375	-1.4436478
C	3.2965326	-11.9702535	0.0000000
C	3.9424442	-11.8040879	1.2650454
C	3.3366364	-12.1079728	2.5464225
C	4.2825795	-11.8246168	3.5064238
C	5.4497944	-11.3243003	2.8081519
N	5.2202821	-11.3365375	1.4436478
C	6.6086372	-10.8329117	3.4276679
C	7.7050356	-10.2153230	2.8085539
C	8.8123441	-9.5930012	3.5066558
C	9.6122637	-9.0142615	2.5466631
C	9.0027629	-9.3113180	1.2651943
N	7.8551213	-10.0418310	1.4436756
C	9.5252659	-8.8971790	0.0000000
H	8.9493246	-9.5871471	-4.5945600
H	10.5370765	-8.4432534	-2.6827832
H	4.1950450	-11.9302924	-4.5943157
H	2.3212960	-12.4957121	-2.6825129
H	2.3212960	-12.4957121	2.6825129
H	4.1950450	-11.9302924	4.5943157
H	8.9493246	-9.5871471	4.5945600
H	10.5370765	-8.4432534	2.6827832
Zn	-6.5388730	10.6915004	0.0000000
C	-4.2825795	11.8246168	3.5064238
C	-3.3366364	12.1079728	2.5464225
C	-3.9424442	11.8040879	1.2650454
C	-5.4497944	11.3243003	2.8081519
N	-5.2202821	11.3365375	1.4436478
C	-6.6086372	10.8329117	3.4276679
C	-7.7050356	10.2153230	2.8085539

C	-8.8123441	9.5930012	3.5066558
C	-9.6122637	9.0142615	2.5466631
C	-9.0027629	9.3113180	1.2651943
N	-7.8551213	10.0418310	1.4436756
C	-9.5252659	8.8971790	0.0000000
C	-9.0027629	9.3113180	-1.2651943
C	-9.6122637	9.0142615	-2.5466631
C	-8.8123441	9.5930012	-3.5066558
C	-7.7050356	10.2153230	-2.8085539
N	-7.8551213	10.0418310	-1.4436756
C	-6.6086372	10.8329117	-3.4276679
C	-5.4497944	11.3243003	-2.8081519
C	-4.2825795	11.8246168	-3.5064238
C	-3.3366364	12.1079728	-2.5464225
C	-3.9424442	11.8040879	-1.2650454
N	-5.2202821	11.3365375	-1.4436478
C	-3.2965326	11.9702535	0.0000000
H	-4.1950450	11.9302924	4.5943157
H	-2.3212960	12.4957121	2.6825129
H	-8.9493246	9.5871471	4.5945600
H	-10.5370765	8.4432534	2.6827832
H	-10.5370765	8.4432534	-2.6827832
H	-8.9493246	9.5871471	-4.5945600
H	-4.1950450	11.9302924	-4.5943157
H	-2.3212960	12.4957121	-2.6825129
Zn	-6.5388730	-10.6915004	0.0000000
C	-3.3366364	-12.1079728	2.5464225
C	-4.2825795	-11.8246168	3.5064238
C	-5.4497944	-11.3243003	2.8081519
C	-3.9424442	-11.8040879	1.2650454
N	-5.2202821	-11.3365375	1.4436478
C	-3.2965326	-11.9702535	0.0000000
C	-3.9424442	-11.8040879	-1.2650454
C	-3.3366364	-12.1079728	-2.5464225
C	-4.2825795	-11.8246168	-3.5064238
C	-5.4497944	-11.3243003	-2.8081519
N	-5.2202821	-11.3365375	-1.4436478
C	-6.6086372	-10.8329117	-3.4276679
C	-7.7050356	-10.2153230	-2.8085539
C	-8.8123441	-9.5930012	-3.5066558
C	-9.6122637	-9.0142615	-2.5466631
C	-9.0027629	-9.3113180	-1.2651943
N	-7.8551213	-10.0418310	-1.4436756
C	-9.5252659	-8.8971790	0.0000000
C	-9.0027629	-9.3113180	1.2651943
C	-9.6122637	-9.0142615	2.5466631
C	-8.8123441	-9.5930012	3.5066558
C	-7.7050356	-10.2153230	2.8085539
N	-7.8551213	-10.0418310	1.4436756

C	-6.6086372	-10.8329117	3.4276679
H	-2.3212960	-12.4957121	2.6825129
H	-4.1950450	-11.9302924	4.5943157
H	-2.3212960	-12.4957121	-2.6825129
H	-4.1950450	-11.9302924	-4.5943157
H	-8.9493246	-9.5871471	-4.5945600
H	-10.5370765	-8.4432534	-2.6827832
H	-10.5370765	-8.4432534	2.6827832
H	-8.9493246	-9.5871471	4.5945600
Zn	11.1266855	0.0000000	13.1495025
C	12.4429144	-3.5070374	10.9946767
C	12.8096322	-2.5466010	10.0785824
C	12.4544995	-1.2652686	10.6566557
C	11.8434835	-2.8092985	12.1146555
N	11.8760406	-1.4443366	11.8879093
C	11.2577327	-3.4288294	13.2280172
C	10.5555348	-2.8090521	14.2723096
C	9.8499820	-3.5068249	15.3284387
C	9.2139778	-2.5462350	16.0830970
C	9.5575880	-1.2651823	15.4983193
N	10.3725159	-1.4442463	14.4088254
C	9.1069011	0.0000000	15.9893334
C	9.5575880	1.2651823	15.4983193
C	9.2139778	2.5462350	16.0830970
C	9.8499820	3.5068249	15.3284387
C	10.5555348	2.8090521	14.2723096
N	10.3725159	1.4442463	14.4088254
C	11.2577327	3.4288294	13.2280172
C	11.8434835	2.8092985	12.1146555
C	12.4429144	3.5070374	10.9946767
C	12.8096322	2.5466010	10.0785824
C	12.4544995	1.2652686	10.6566557
N	11.8760406	1.4443366	11.8879093
C	12.6807065	0.0000000	10.0300630
H	12.5555181	-4.5949153	10.9159859
H	13.2860222	-2.6821960	9.1016339
H	9.8330551	-4.5947565	15.4643410
H	8.5752298	-2.6816613	16.9625726
H	8.5752298	2.6816613	16.9625726
H	9.8330551	4.5947565	15.4643410
H	12.5555181	4.5949153	10.9159859
H	13.2860222	2.6821960	9.1016339
Zn	11.1266855	0.0000000	-13.1495025
C	9.8499820	-3.5068249	-15.3284387
C	9.2139778	-2.5462350	-16.0830970
C	9.5575880	-1.2651823	-15.4983193
C	10.5555348	-2.8090521	-14.2723096
N	10.3725159	-1.4442463	-14.4088254
C	11.2577327	-3.4288294	-13.2280172

C	11.8434835	-2.8092985	-12.1146555
C	12.4429144	-3.5070374	-10.9946767
C	12.8096322	-2.5466010	-10.0785824
C	12.4544995	-1.2652686	-10.6566557
N	11.8760406	-1.4443366	-11.8879093
C	12.6807065	0.0000000	-10.0300630
C	12.4544995	1.2652686	-10.6566557
C	12.8096322	2.5466010	-10.0785824
C	12.4429144	3.5070374	-10.9946767
C	11.8434835	2.8092985	-12.1146555
N	11.8760406	1.4443366	-11.8879093
C	11.2577327	3.4288294	-13.2280172
C	10.5555348	2.8090521	-14.2723096
C	9.8499820	3.5068249	-15.3284387
C	9.2139778	2.5462350	-16.0830970
C	9.5575880	1.2651823	-15.4983193
N	10.3725159	1.4442463	-14.4088254
C	9.1069011	0.0000000	-15.9893334
H	9.8330551	-4.5947565	-15.4643410
H	8.5752298	-2.6816613	-16.9625726
H	12.5555181	-4.5949153	-10.9159859
H	13.2860222	-2.6821960	-9.1016339
H	13.2860222	2.6821960	-9.1016339
H	12.5555181	4.5949153	-10.9159859
H	9.8330551	4.5947565	-15.4643410
H	8.5752298	2.6816613	-16.9625726
Zn	-11.1266855	0.0000000	-13.1495025
C	-12.4429144	-3.5070374	-10.9946767
C	-12.8096322	-2.5466010	-10.0785824
C	-12.4544995	-1.2652686	-10.6566557
C	-11.8434835	-2.8092985	-12.1146555
N	-11.8760406	-1.4443366	-11.8879093
C	-11.2577327	-3.4288294	-13.2280172
C	-10.5555348	-2.8090521	-14.2723096
C	-9.8499820	-3.5068249	-15.3284387
C	-9.2139778	-2.5462350	-16.0830970
C	-9.5575880	-1.2651823	-15.4983193
N	-10.3725159	-1.4442463	-14.4088254
C	-9.1069011	0.0000000	-15.9893334
C	-9.5575880	1.2651823	-15.4983193
C	-9.2139778	2.5462350	-16.0830970
C	-9.8499820	3.5068249	-15.3284387
C	-10.5555348	2.8090521	-14.2723096
N	-10.3725159	1.4442463	-14.4088254
C	-11.2577327	3.4288294	-13.2280172
C	-11.8434835	2.8092985	-12.1146555
C	-12.4429144	3.5070374	-10.9946767
C	-12.8096322	2.5466010	-10.0785824
C	-12.4544995	1.2652686	-10.6566557

N	-11.8760406	1.4443366	-11.8879093
C	-12.6807065	0.0000000	-10.0300630
H	-12.5555181	-4.5949153	-10.9159859
H	-13.2860222	-2.6821960	-9.1016339
H	-9.8330551	-4.5947565	-15.4643410
H	-8.5752298	-2.6816613	-16.9625726
H	-8.5752298	2.6816613	-16.9625726
H	-9.8330551	4.5947565	-15.4643410
H	-12.5555181	4.5949153	-10.9159859
H	-13.2860222	2.6821960	-9.1016339
Zn	-11.1266855	0.0000000	13.1495025
C	-9.8499820	-3.5068249	15.3284387
C	-9.2139778	-2.5462350	16.0830970
C	-9.5575880	-1.2651823	15.4983193
C	-10.5555348	-2.8090521	14.2723096
N	-10.3725159	-1.4442463	14.4088254
C	-11.2577327	-3.4288294	13.2280172
C	-11.8434835	-2.8092985	12.1146555
C	-12.4429144	-3.5070374	10.9946767
C	-12.8096322	-2.5466010	10.0785824
C	-12.4544995	-1.2652686	10.6566557
N	-11.8760406	-1.4443366	11.8879093
C	-12.6807065	0.0000000	10.0300630
C	-12.4544995	1.2652686	10.6566557
C	-12.8096322	2.5466010	10.0785824
C	-12.4429144	3.5070374	10.9946767
C	-11.8434835	2.8092985	12.1146555
N	-11.8760406	1.4443366	11.8879093
C	-11.2577327	3.4288294	13.2280172
C	-10.5555348	2.8090521	14.2723096
C	-9.8499820	3.5068249	15.3284387
C	-9.2139778	2.5462350	16.0830970
C	-9.5575880	1.2651823	15.4983193
N	-10.3725159	1.4442463	14.4088254
C	-9.1069011	0.0000000	15.9893334
H	-9.8330551	-4.5947565	15.4643410
H	-8.5752298	-2.6816613	16.9625726
H	-12.5555181	-4.5949153	10.9159859
H	-13.2860222	-2.6821960	9.1016339
H	-13.2860222	2.6821960	9.1016339
H	-12.5555181	4.5949153	10.9159859
H	-9.8330551	4.5947565	15.4643410
H	-8.5752298	2.6816613	16.9625726
Zn	0.0000000	0.0000000	19.6749312
C	2.5244958	-3.5053536	19.6965401
C	3.4952022	-2.5459900	19.5106014
C	2.8163802	-1.2652277	19.5054518
C	1.2563837	-2.8077729	19.7726430
N	1.4661311	-1.4437321	19.6706506

C	0.0000000	-3.4267963	19.8522129
C	-1.2563837	-2.8077729	19.7726430
C	-2.5244958	-3.5053536	19.6965401
C	-3.4952022	-2.5459900	19.5106014
C	-2.8163802	-1.2652277	19.5054518
N	-1.4661311	-1.4437321	19.6706506
C	-3.4647686	0.0000000	19.3490600
C	-2.8163802	1.2652277	19.5054518
C	-3.4952022	2.5459900	19.5106014
C	-2.5244958	3.5053536	19.6965401
C	-1.2563837	2.8077729	19.7726430
N	-1.4661311	1.4437321	19.6706506
C	0.0000000	3.4267963	19.8522129
C	1.2563837	2.8077729	19.7726430
C	2.5244958	3.5053536	19.6965401
C	3.4952022	2.5459900	19.5106014
C	2.8163802	1.2652277	19.5054518
N	1.4661311	1.4437321	19.6706506
C	3.4647686	0.0000000	19.3490600
H	2.6516774	-4.5925984	19.7604805
H	4.5760943	-2.6821450	19.3963114
H	-2.6516774	-4.5925984	19.7604805
H	-4.5760943	-2.6821450	19.3963114
H	-4.5760943	2.6821450	19.3963114
H	-2.6516774	4.5925984	19.7604805
H	2.6516774	4.5925984	19.7604805
H	4.5760943	2.6821450	19.3963114
Zn	0.0000000	0.0000000	-19.6749312
C	2.5244958	-3.5053536	-19.6965401
C	3.4952022	-2.5459900	-19.5106014
C	2.8163802	-1.2652277	-19.5054518
C	1.2563837	-2.8077729	-19.7726430
N	1.4661311	-1.4437321	-19.6706506
C	0.0000000	-3.4267963	-19.8522129
C	-1.2563837	-2.8077729	-19.7726430
C	-2.5244958	-3.5053536	-19.6965401
C	-3.4952022	-2.5459900	-19.5106014
C	-2.8163802	-1.2652277	-19.5054518
N	-1.4661311	-1.4437321	-19.6706506
C	-3.4647686	0.0000000	-19.3490600
C	-2.8163802	1.2652277	-19.5054518
C	-3.4952022	2.5459900	-19.5106014
C	-2.5244958	3.5053536	-19.6965401
C	-1.2563837	2.8077729	-19.7726430
N	-1.4661311	1.4437321	-19.6706506
C	0.0000000	3.4267963	-19.8522129
C	1.2563837	2.8077729	-19.7726430
C	2.5244958	3.5053536	-19.6965401
C	3.4952022	2.5459900	-19.5106014

C	2.8163802	1.2652277	-19.5054518
N	1.4661311	1.4437321	-19.6706506
C	3.4647686	0.0000000	-19.3490600
H	2.6516774	-4.5925984	-19.7604805
H	4.5760943	-2.6821450	-19.3963114
H	-2.6516774	-4.5925984	-19.7604805
H	-4.5760943	-2.6821450	-19.3963114
H	-4.5760943	2.6821450	-19.3963114
H	-2.6516774	4.5925984	-19.7604805
H	2.6516774	4.5925984	-19.7604805
H	4.5760943	2.6821450	-19.3963114
C	12.7171825	-4.7870452	0.0000000
C	12.1419039	-5.8910404	0.0000000
C	11.3880098	-7.0125675	0.0000000
C	10.5773416	-7.9575700	0.0000000
C	13.6439955	0.0000000	4.8903479
C	13.5563918	0.0000000	6.1311917
C	13.3704191	0.0000000	7.4696467
C	13.1089162	0.0000000	8.6863028
C	12.7171825	4.7870452	0.0000000
C	12.1419039	5.8910404	0.0000000
C	11.3880098	7.0125675	0.0000000
C	10.5773416	7.9575700	0.0000000
C	13.6439955	0.0000000	-4.8903479
C	13.5563918	0.0000000	-6.1311917
C	13.3704191	0.0000000	-7.4696467
C	13.1089162	0.0000000	-8.6863028
C	-12.7171825	-4.7870452	0.0000000
C	-12.1419039	-5.8910404	0.0000000
C	-11.3880098	-7.0125675	0.0000000
C	-10.5773416	-7.9575700	0.0000000
C	-13.6439955	0.0000000	-4.8903479
C	-13.5563918	0.0000000	-6.1311917
C	-13.3704191	0.0000000	-7.4696467
C	-13.1089162	0.0000000	-8.6863028
C	-12.7171825	4.7870452	0.0000000
C	-12.1419039	5.8910404	0.0000000
C	-11.3880098	7.0125675	0.0000000
C	-10.5773416	7.9575700	0.0000000
C	-13.6439955	0.0000000	4.8903479
C	-13.5563918	0.0000000	6.1311917
C	-13.3704191	0.0000000	7.4696467
C	-13.1089162	0.0000000	8.6863028
C	1.9155467	12.2578089	0.0000000
C	0.6757151	12.3697128	0.0000000
C	-0.6757151	12.3697128	0.0000000
C	-1.9155467	12.2578089	0.0000000
C	1.9155467	-12.2578089	0.0000000
C	0.6757151	-12.3697128	0.0000000

C	-0.6757151	-12.3697128	0.0000000
C	-1.9155467	-12.2578089	0.0000000
C	8.0983608	0.0000000	16.9751585
C	7.1039856	0.0000000	17.7241248
C	5.9449750	0.0000000	18.4185571
C	4.8143946	0.0000000	18.9401110
C	8.0983608	0.0000000	-16.9751585
C	7.1039856	0.0000000	-17.7241248
C	5.9449750	0.0000000	-18.4185571
C	4.8143946	0.0000000	-18.9401110
C	-8.0983608	0.0000000	-16.9751585
C	-7.1039856	0.0000000	-17.7241248
C	-5.9449750	0.0000000	-18.4185571
C	-4.8143946	0.0000000	-18.9401110
C	-8.0983608	0.0000000	16.9751585
C	-7.1039856	0.0000000	17.7241248
C	-5.9449750	0.0000000	18.4185571
C	-4.8143946	0.0000000	18.9401110
H	6.6347209	10.8863764	4.5261218
H	6.6347209	10.8863764	-4.5261218
H	6.6347209	-10.8863764	-4.5261218
H	6.6347209	-10.8863764	4.5261218
H	-6.6347209	10.8863764	4.5261218
H	-6.6347209	10.8863764	-4.5261218
H	-6.6347209	-10.8863764	-4.5261218
H	-6.6347209	-10.8863764	4.5261218
H	11.3083995	-4.5273062	13.2583139
H	11.3083995	4.5273062	13.2583139
H	11.3083995	-4.5273062	-13.2583139
H	11.3083995	4.5273062	-13.2583139
H	-11.3083995	-4.5273062	-13.2583139
H	-11.3083995	4.5273062	-13.2583139
H	-11.3083995	-4.5273062	13.2583139
H	-11.3083995	4.5273062	13.2583139
H	0.0000000	-4.5247636	19.9200585
H	0.0000000	4.5247636	19.9200585
H	0.0000000	-4.5247636	-19.9200585
H	0.0000000	4.5247636	-19.9200585

Molecule 3

550
Energy =

Zn	0.0000000	0.0000000	13.7409557
C	2.5354998	-3.5018209	13.7658056
C	3.4901049	-2.5366947	13.5260780
C	2.8000768	-1.2664886	13.5177641
C	1.2665761	-2.8145395	13.8636714
N	1.4526435	-1.4560619	13.7298631
C	0.0000000	-3.4658292	13.9809325

C	-1.2665761	-2.8145395	13.8636714
C	-2.5354998	-3.5018209	13.7658056
C	-3.4901049	-2.5366947	13.5260780
C	-2.8000768	-1.2664886	13.5177641
N	-1.4526435	-1.4560619	13.7298631
C	-3.4319573	0.0000000	13.3234382
C	-2.8000768	1.2664886	13.5177641
C	-3.4901049	2.5366947	13.5260780
C	-2.5354998	3.5018209	13.7658056
C	-1.2665761	2.8145395	13.8636714
N	-1.4526435	1.4560619	13.7298631
C	0.0000000	3.4658292	13.9809325
C	1.2665761	2.8145395	13.8636714
C	2.5354998	3.5018209	13.7658056
C	3.4901049	2.5366947	13.5260780
C	2.8000768	1.2664886	13.5177641
N	1.4526435	1.4560619	13.7298631
C	3.4319573	0.0000000	13.3234382
H	2.6704478	-4.5854943	13.8475975
H	4.5677390	-2.6670018	13.3800023
H	-2.6704478	-4.5854943	13.8475975
H	-4.5677390	-2.6670018	13.3800023
H	-4.5677390	2.6670018	13.3800023
H	-2.6704478	4.5854943	13.8475975
H	2.6704478	4.5854943	13.8475975
H	4.5677390	2.6670018	13.3800023
Zn	10.5615091	0.0000000	6.5547937
H	10.7548391	4.5263309	6.6468561
H	10.7548391	-4.5263309	6.6468561
C	11.9406957	2.5464542	3.3359610
C	11.6660632	3.5065863	4.2842928
C	11.1791740	2.8082744	5.4571346
C	11.6444212	1.2650071	3.9455011
N	11.1901446	1.4436838	5.2281306
C	11.8055684	0.0000000	3.2984114
C	11.6444212	-1.2650071	3.9455011
C	11.9406957	-2.5464542	3.3359610
C	11.6660632	-3.5065863	4.2842928
C	11.1791740	-2.8082744	5.4571346
N	11.1901446	-1.4436838	5.2281306
C	10.7016923	-3.4278336	6.6217935
C	10.0991340	-2.8086827	7.7264949
C	9.4912590	-3.5066996	8.8418564
C	8.9236919	-2.5465100	9.6494859
C	9.2128575	-1.2650854	9.0361738
N	9.9279386	-1.4437002	7.8788672
C	8.8057508	0.0000000	9.5643097
C	9.2128575	1.2650854	9.0361738
C	8.9236919	2.5465100	9.6494859

C	9.4912590	3.5066996	8.8418564
C	10.0991340	2.8086827	7.7264949
N	9.9279386	1.4437002	7.8788672
C	10.7016923	3.4278336	6.6217935
H	12.3176866	2.6824488	2.3165819
H	11.7696958	4.5945481	4.1952079
H	12.3176866	-2.6824488	2.3165819
H	11.7696958	-4.5945481	4.1952079
H	9.4867931	-4.5945925	8.9789411
H	8.3651453	-2.6825608	10.5818378
H	8.3651453	2.6825608	10.5818378
H	9.4867931	4.5945925	8.9789411
Zn	-10.5615091	0.0000000	6.5547937
H	-10.7548391	4.5263309	6.6468561
H	-10.7548391	-4.5263309	6.6468561
C	-9.4912590	-3.5066996	8.8418564
C	-8.9236919	-2.5465100	9.6494859
C	-9.2128575	-1.2650854	9.0361738
C	-10.0991340	-2.8086827	7.7264949
N	-9.9279386	-1.4437002	7.8788672
C	-10.7016923	-3.4278336	6.6217935
C	-11.1791740	-2.8082744	5.4571346
C	-11.6660632	-3.5065863	4.2842928
C	-11.9406957	-2.5464542	3.3359610
C	-11.6444212	-1.2650071	3.9455011
N	-11.1901446	-1.4436838	5.2281306
C	-11.8055684	0.0000000	3.2984114
C	-11.6444212	1.2650071	3.9455011
C	-11.9406957	2.5464542	3.3359610
C	-11.6660632	3.5065863	4.2842928
C	-11.1791740	2.8082744	5.4571346
N	-11.1901446	1.4436838	5.2281306
C	-10.7016923	3.4278336	6.6217935
C	-10.0991340	2.8086827	7.7264949
C	-9.4912590	3.5066996	8.8418564
C	-8.9236919	2.5465100	9.6494859
C	-9.2128575	1.2650854	9.0361738
N	-9.9279386	1.4437002	7.8788672
C	-8.8057508	0.0000000	9.5643097
H	-9.4867931	-4.5945925	8.9789411
H	-8.3651453	-2.6825608	10.5818378
H	-11.7696958	-4.5945481	4.1952079
H	-12.3176866	-2.6824488	2.3165819
H	-12.3176866	2.6824488	2.3165819
H	-11.7696958	4.5945481	4.1952079
H	-9.4867931	4.5945925	8.9789411
H	-8.3651453	2.6825608	10.5818378
Zn	0.0000000	0.0000000	-13.7409557
C	2.5354998	3.5018209	-13.7658056

C	3.4901049	2.5366947	-13.5260780
C	2.8000768	1.2664886	-13.5177641
C	1.2665761	2.8145395	-13.8636714
N	1.4526435	1.4560619	-13.7298631
C	0.0000000	3.4658292	-13.9809325
C	-1.2665761	2.8145395	-13.8636714
C	-2.5354998	3.5018209	-13.7658056
C	-3.4901049	2.5366947	-13.5260780
C	-2.8000768	1.2664886	-13.5177641
N	-1.4526435	1.4560619	-13.7298631
C	-3.4319573	0.0000000	-13.3234382
C	-2.8000768	-1.2664886	-13.5177641
C	-3.4901049	-2.5366947	-13.5260780
C	-2.5354998	-3.5018209	-13.7658056
C	-1.2665761	-2.8145395	-13.8636714
N	-1.4526435	-1.4560619	-13.7298631
C	0.0000000	-3.4658292	-13.9809325
C	1.2665761	-2.8145395	-13.8636714
C	2.5354998	-3.5018209	-13.7658056
C	3.4901049	-2.5366947	-13.5260780
C	2.8000768	-1.2664886	-13.5177641
N	1.4526435	-1.4560619	-13.7298631
C	3.4319573	0.0000000	-13.3234382
H	2.6704478	4.5854943	-13.8475975
H	4.5677390	2.6670018	-13.3800023
H	-2.6704478	4.5854943	-13.8475975
H	-4.5677390	2.6670018	-13.3800023
H	-4.5677390	-2.6670018	-13.3800023
H	-2.6704478	-4.5854943	-13.8475975
H	2.6704478	-4.5854943	-13.8475975
H	4.5677390	-2.6670018	-13.3800023
Zn	-10.5615091	0.0000000	-6.5547937
H	-10.7548391	4.5263309	-6.6468561
H	-10.7548391	-4.5263309	-6.6468561
C	-8.9236919	-2.5465100	-9.6494859
C	-9.4912590	-3.5066996	-8.8418564
C	-10.0991340	-2.8086827	-7.7264949
C	-9.2128575	-1.2650854	-9.0361738
N	-9.9279386	-1.4437002	-7.8788672
C	-8.8057508	0.0000000	-9.5643097
C	-9.2128575	1.2650854	-9.0361738
C	-8.9236919	2.5465100	-9.6494859
C	-9.4912590	3.5066996	-8.8418564
C	-10.0991340	2.8086827	-7.7264949
N	-9.9279386	1.4437002	-7.8788672
C	-10.7016923	3.4278336	-6.6217935
C	-11.1791740	2.8082744	-5.4571346
C	-11.6660632	3.5065863	-4.2842928
C	-11.9406957	2.5464542	-3.3359610

C	-11.6444212	1.2650071	-3.9455011
N	-11.1901446	1.4436838	-5.2281306
C	-11.8055684	0.0000000	-3.2984114
C	-11.6444212	-1.2650071	-3.9455011
C	-11.9406957	-2.5464542	-3.3359610
C	-11.6660632	-3.5065863	-4.2842928
C	-11.1791740	-2.8082744	-5.4571346
N	-11.1901446	-1.4436838	-5.2281306
C	-10.7016923	-3.4278336	-6.6217935
H	-8.3651453	-2.6825608	-10.5818378
H	-9.4867931	-4.5945925	-8.9789411
H	-8.3651453	2.6825608	-10.5818378
H	-9.4867931	4.5945925	-8.9789411
H	-11.7696958	4.5945481	-4.1952079
H	-12.3176866	2.6824488	-2.3165819
H	-12.3176866	-2.6824488	-2.3165819
H	-11.7696958	-4.5945481	-4.1952079
Zn	10.5615091	0.0000000	-6.5547937
H	10.7548391	4.5263309	-6.6468561
H	10.7548391	-4.5263309	-6.6468561
C	11.6660632	3.5065863	-4.2842928
C	11.9406957	2.5464542	-3.3359610
C	11.6444212	1.2650071	-3.9455011
C	11.1791740	2.8082744	-5.4571346
N	11.1901446	1.4436838	-5.2281306
C	10.7016923	3.4278336	-6.6217935
C	10.0991340	2.8086827	-7.7264949
C	9.4912590	3.5066996	-8.8418564
C	8.9236919	2.5465100	-9.6494859
C	9.2128575	1.2650854	-9.0361738
N	9.9279386	1.4437002	-7.8788672
C	8.8057508	0.0000000	-9.5643097
C	9.2128575	-1.2650854	-9.0361738
C	8.9236919	-2.5465100	-9.6494859
C	9.4912590	-3.5066996	-8.8418564
C	10.0991340	-2.8086827	-7.7264949
N	9.9279386	-1.4437002	-7.8788672
C	10.7016923	-3.4278336	-6.6217935
C	11.1791740	-2.8082744	-5.4571346
C	11.6660632	-3.5065863	-4.2842928
C	11.9406957	-2.5464542	-3.3359610
C	11.6444212	-1.2650071	-3.9455011
N	11.1901446	-1.4436838	-5.2281306
C	11.8055684	0.0000000	-3.2984114
H	11.7696958	4.5945481	-4.1952079
H	12.3176866	2.6824488	-2.3165819
H	9.4867931	4.5945925	-8.9789411
H	8.3651453	2.6825608	-10.5818378
H	8.3651453	-2.6825608	-10.5818378

H	9.4867931	-4.5945925	-8.9789411
H	11.7696958	-4.5945481	-4.1952079
H	12.3176866	-2.6824488	-2.3165819
Zn	0.0000000	-13.5662028	13.7194043
H	4.5311934	-13.5961453	13.8669725
H	-4.5311934	-13.5961453	13.8669725
C	2.5467903	-16.9830952	12.9355437
C	3.5090535	-16.0458768	13.2381306
C	2.8110254	-14.8105280	13.5321122
C	1.2651441	-16.3182682	13.0655706
N	1.4449969	-15.0072686	13.4304285
C	0.0000000	-16.9450153	12.8409228
C	-1.2651441	-16.3182682	13.0655706
C	-2.5467903	-16.9830952	12.9355437
C	-3.5090535	-16.0458768	13.2381306
C	-2.8110254	-14.8105280	13.5321122
N	-1.4449969	-15.0072686	13.4304285
C	-3.4319665	-13.5877531	13.8255964
C	-2.8113864	-12.3468097	14.0265358
C	-3.5092810	-11.0947219	14.2395881
C	-2.5472106	-10.1138519	14.3267016
C	-1.2653857	-10.7763199	14.1834793
N	-1.4452694	-12.1251728	14.0055303
C	0.0000000	-10.1124618	14.2278118
C	1.2653857	-10.7763199	14.1834793
C	2.5472106	-10.1138519	14.3267016
C	3.5092810	-11.0947219	14.2395881
C	2.8113864	-12.3468097	14.0265358
N	1.4452694	-12.1251728	14.0055303
C	3.4319665	-13.5877531	13.8255964
H	2.6813642	-18.0332088	12.6546535
H	4.5979585	-16.1740417	13.2522932
H	-2.6813642	-18.0332088	12.6546535
H	-4.5979585	-16.1740417	13.2522932
H	-4.5980554	-10.9824732	14.3049463
H	-2.6819319	-9.0378844	14.4806198
H	2.6819319	-9.0378844	14.4806198
H	4.5980554	-10.9824732	14.3049463
Zn	0.0000000	13.5662028	13.7194043
H	4.5311934	13.5961453	13.8669725
H	-4.5311934	13.5961453	13.8669725
C	2.5472106	10.1138519	14.3267016
C	3.5092810	11.0947219	14.2395881
C	2.8113864	12.3468097	14.0265358
C	1.2653857	10.7763199	14.1834793
N	1.4452694	12.1251728	14.0055303
C	0.0000000	10.1124618	14.2278118
C	-1.2653857	10.7763199	14.1834793
C	-2.5472106	10.1138519	14.3267016

C	-3.5092810	11.0947219	14.2395881
C	-2.8113864	12.3468097	14.0265358
N	-1.4452694	12.1251728	14.0055303
C	-3.4319665	13.5877531	13.8255964
C	-2.8110254	14.8105280	13.5321122
C	-3.5090535	16.0458768	13.2381306
C	-2.5467903	16.9830952	12.9355437
C	-1.2651441	16.3182682	13.0655706
N	-1.4449969	15.0072686	13.4304285
C	0.0000000	16.9450153	12.8409228
C	1.2651441	16.3182682	13.0655706
C	2.5467903	16.9830952	12.9355437
C	3.5090535	16.0458768	13.2381306
C	2.8110254	14.8105280	13.5321122
N	1.4449969	15.0072686	13.4304285
C	3.4319665	13.5877531	13.8255964
H	2.6819319	9.0378844	14.4806198
H	4.5980554	10.9824732	14.3049463
H	-2.6819319	9.0378844	14.4806198
H	-4.5980554	10.9824732	14.3049463
H	-4.5979585	16.1740417	13.2522932
H	-2.6813642	18.0332088	12.6546535
H	2.6813642	18.0332088	12.6546535
H	4.5979585	16.1740417	13.2522932
Zn	0.0000000	-13.5662028	-13.7194043
H	4.5311934	-13.5961453	-13.8669725
H	-4.5311934	-13.5961453	-13.8669725
C	2.5472106	-10.1138519	-14.3267016
C	3.5092810	-11.0947219	-14.2395881
C	2.8113864	-12.3468097	-14.0265358
C	1.2653857	-10.7763199	-14.1834793
N	1.4452694	-12.1251728	-14.0055303
C	0.0000000	-10.1124618	-14.2278118
C	-1.2653857	-10.7763199	-14.1834793
C	-2.5472106	-10.1138519	-14.3267016
C	-3.5092810	-11.0947219	-14.2395881
C	-2.8113864	-12.3468097	-14.0265358
N	-1.4452694	-12.1251728	-14.0055303
C	-3.4319665	-13.5877531	-13.8255964
C	-2.8110254	-14.8105280	-13.5321122
C	-3.5090535	-16.0458768	-13.2381306
C	-2.5467903	-16.9830952	-12.9355437
C	-1.2651441	-16.3182682	-13.0655706
N	-1.4449969	-15.0072686	-13.4304285
C	0.0000000	-16.9450153	-12.8409228
C	1.2651441	-16.3182682	-13.0655706
C	2.5467903	-16.9830952	-12.9355437
C	3.5090535	-16.0458768	-13.2381306
C	2.8110254	-14.8105280	-13.5321122

N	1.4449969	-15.0072686	-13.4304285
C	3.4319665	-13.5877531	-13.8255964
H	2.6819319	-9.0378844	-14.4806198
H	4.5980554	-10.9824732	-14.3049463
H	-2.6819319	-9.0378844	-14.4806198
H	-4.5980554	-10.9824732	-14.3049463
H	-4.5979585	-16.1740417	-13.2522932
H	-2.6813642	-18.0332088	-12.6546535
H	2.6813642	-18.0332088	-12.6546535
H	4.5979585	-16.1740417	-13.2522932
Zn	0.0000000	13.5662028	-13.7194043
H	4.5311934	13.5961453	-13.8669725
H	-4.5311934	13.5961453	-13.8669725
C	2.5467903	16.9830952	-12.9355437
C	3.5090535	16.0458768	-13.2381306
C	2.8110254	14.8105280	-13.5321122
C	1.2651441	16.3182682	-13.0655706
N	1.4449969	15.0072686	-13.4304285
C	0.0000000	16.9450153	-12.8409228
C	-1.2651441	16.3182682	-13.0655706
C	-2.5467903	16.9830952	-12.9355437
C	-3.5090535	16.0458768	-13.2381306
C	-2.8110254	14.8105280	-13.5321122
N	-1.4449969	15.0072686	-13.4304285
C	-3.4319665	13.5877531	-13.8255964
C	-2.8113864	12.3468097	-14.0265358
C	-3.5092810	11.0947219	-14.2395881
C	-2.5472106	10.1138519	-14.3267016
C	-1.2653857	10.7763199	-14.1834793
N	-1.4452694	12.1251728	-14.0055303
C	0.0000000	10.1124618	-14.2278118
C	1.2653857	10.7763199	-14.1834793
C	2.5472106	10.1138519	-14.3267016
C	3.5092810	11.0947219	-14.2395881
C	2.8113864	12.3468097	-14.0265358
N	1.4452694	12.1251728	-14.0055303
C	3.4319665	13.5877531	-13.8255964
H	2.6813642	18.0332088	-12.6546535
H	4.5979585	16.1740417	-13.2522932
H	-2.6813642	18.0332088	-12.6546535
H	-4.5979585	16.1740417	-13.2522932
H	-4.5980554	10.9824732	-14.3049463
H	-2.6819319	9.0378844	-14.4806198
H	2.6819319	9.0378844	-14.4806198
H	4.5980554	10.9824732	-14.3049463
Zn	0.0000000	-24.5095657	6.4229608
H	4.5260246	-24.7088240	6.5404045
H	-4.5260246	-24.7088240	6.5404045
C	2.5460698	-26.1542247	3.3331860

C	3.5059569	-25.8116031	4.2595717
C	2.8084029	-25.2287661	5.3883148
C	1.2651989	-25.8051651	3.9152128
N	1.4439838	-25.2530191	5.1585408
C	0.0000000	-26.0071420	3.2798746
C	-1.2651989	-25.8051651	3.9152128
C	-2.5460698	-26.1542247	3.3331860
C	-3.5059569	-25.8116031	4.2595717
C	-2.8084029	-25.2287661	5.3883148
N	-1.4439838	-25.2530191	5.1585408
C	-3.4278346	-24.6535855	6.5077756
C	-2.8084579	-23.9497355	7.5510983
C	-3.5060804	-23.2452813	8.6081915
C	-2.5462262	-22.6020597	9.3576808
C	-1.2652358	-22.9412848	8.7699483
N	-1.4440392	-23.7595899	7.6831916
C	0.0000000	-22.4868630	9.2576598
C	1.2652358	-22.9412848	8.7699483
C	2.5462262	-22.6020597	9.3576808
C	3.5060804	-23.2452813	8.6081915
C	2.8084579	-23.9497355	7.5510983
N	1.4440392	-23.7595899	7.6831916
C	3.4278346	-24.6535855	6.5077756
H	2.6817741	-26.6108439	2.3468006
H	4.5934882	-25.9278987	4.1813289
H	-2.6817741	-26.6108439	2.3468006
H	-4.5934882	-25.9278987	4.1813289
H	-4.5936110	-23.2334068	8.7478214
H	-2.6820684	-21.9618299	10.2360089
H	2.6820684	-21.9618299	10.2360089
H	4.5936110	-23.2334068	8.7478214
Zn	0.0000000	24.5095657	6.4229608
H	4.5260246	24.7088240	6.5404045
H	-4.5260246	24.7088240	6.5404045
C	2.5462262	22.6020597	9.3576808
C	3.5060804	23.2452813	8.6081915
C	2.8084579	23.9497355	7.5510983
C	1.2652358	22.9412848	8.7699483
N	1.4440392	23.7595899	7.6831916
C	0.0000000	22.4868630	9.2576598
C	-1.2652358	22.9412848	8.7699483
C	-2.5462262	22.6020597	9.3576808
C	-3.5060804	23.2452813	8.6081915
C	-2.8084579	23.9497355	7.5510983
N	-1.4440392	23.7595899	7.6831916
C	-3.4278346	24.6535855	6.5077756
C	-2.8084029	25.2287661	5.3883148
C	-3.5059569	25.8116031	4.2595717
C	-2.5460698	26.1542247	3.3331860

C	-1.2651989	25.8051651	3.9152128
N	-1.4439838	25.2530191	5.1585408
C	0.0000000	26.0071420	3.2798746
C	1.2651989	25.8051651	3.9152128
C	2.5460698	26.1542247	3.3331860
C	3.5059569	25.8116031	4.2595717
C	2.8084029	25.2287661	5.3883148
N	1.4439838	25.2530191	5.1585408
C	3.4278346	24.6535855	6.5077756
H	2.6820684	21.9618299	10.2360089
H	4.5936110	23.2334068	8.7478214
H	-2.6820684	21.9618299	10.2360089
H	-4.5936110	23.2334068	8.7478214
H	-4.5934882	25.9278987	4.1813289
H	-2.6817741	26.6108439	2.3468006
H	2.6817741	26.6108439	2.3468006
H	4.5934882	25.9278987	4.1813289
Zn	0.0000000	-24.5095657	-6.4229608
H	4.5260246	-24.7088240	-6.5404045
H	-4.5260246	-24.7088240	-6.5404045
C	2.5462262	-22.6020597	-9.3576808
C	3.5060804	-23.2452813	-8.6081915
C	2.8084579	-23.9497355	-7.5510983
C	1.2652358	-22.9412848	-8.7699483
N	1.4440392	-23.7595899	-7.6831916
C	0.0000000	-22.4868630	-9.2576598
C	-1.2652358	-22.9412848	-8.7699483
C	-2.5462262	-22.6020597	-9.3576808
C	-3.5060804	-23.2452813	-8.6081915
C	-2.8084579	-23.9497355	-7.5510983
N	-1.4440392	-23.7595899	-7.6831916
C	-3.4278346	-24.6535855	-6.5077756
C	-2.8084029	-25.2287661	-5.3883148
C	-3.5059569	-25.8116031	-4.2595717
C	-2.5460698	-26.1542247	-3.3331860
C	-1.2651989	-25.8051651	-3.9152128
N	-1.4439838	-25.2530191	-5.1585408
C	0.0000000	-26.0071420	-3.2798746
C	1.2651989	-25.8051651	-3.9152128
C	2.5460698	-26.1542247	-3.3331860
C	3.5059569	-25.8116031	-4.2595717
C	2.8084029	-25.2287661	-5.3883148
N	1.4439838	-25.2530191	-5.1585408
C	3.4278346	-24.6535855	-6.5077756
H	2.6820684	-21.9618299	-10.2360089
H	4.5936110	-23.2334068	-8.7478214
H	-2.6820684	-21.9618299	-10.2360089
H	-4.5936110	-23.2334068	-8.7478214
H	-4.5934882	-25.9278987	-4.1813289

H	-2.6817741	-26.6108439	-2.3468006
H	2.6817741	-26.6108439	-2.3468006
H	4.5934882	-25.9278987	-4.1813289
Zn	0.0000000	24.5095657	-6.4229608
H	4.5260246	24.7088240	-6.5404045
H	-4.5260246	24.7088240	-6.5404045
C	2.5460698	26.1542247	-3.3331860
C	3.5059569	25.8116031	-4.2595717
C	2.8084029	25.2287661	-5.3883148
C	1.2651989	25.8051651	-3.9152128
N	1.4439838	25.2530191	-5.1585408
C	0.0000000	26.0071420	-3.2798746
C	-1.2651989	25.8051651	-3.9152128
C	-2.5460698	26.1542247	-3.3331860
C	-3.5059569	25.8116031	-4.2595717
C	-2.8084029	25.2287661	-5.3883148
N	-1.4439838	25.2530191	-5.1585408
C	-3.4278346	24.6535855	-6.5077756
C	-2.8084579	23.9497355	-7.5510983
C	-3.5060804	23.2452813	-8.6081915
C	-2.5462262	22.6020597	-9.3576808
C	-1.2652358	22.9412848	-8.7699483
N	-1.4440392	23.7595899	-7.6831916
C	0.0000000	22.4868630	-9.2576598
C	1.2652358	22.9412848	-8.7699483
C	2.5462262	22.6020597	-9.3576808
C	3.5060804	23.2452813	-8.6081915
C	2.8084579	23.9497355	-7.5510983
N	1.4440392	23.7595899	-7.6831916
C	3.4278346	24.6535855	-6.5077756
H	2.6817741	26.6108439	-2.3468006
H	4.5934882	25.9278987	-4.1813289
H	-2.6817741	26.6108439	-2.3468006
H	-4.5934882	25.9278987	-4.1813289
H	-4.5936110	23.2334068	-8.7478214
H	-2.6820684	21.9618299	-10.2360089
H	2.6820684	21.9618299	-10.2360089
H	4.5936110	23.2334068	-8.7478214
C	0.0000000	-4.8698930	14.1220381
C	0.0000000	-6.1098311	14.2207048
C	0.0000000	-7.4595663	14.2838279
C	0.0000000	-8.7036569	14.2949012
C	-4.7577692	0.0000000	12.8393072
C	-5.8466161	0.0000000	12.2356106
C	-6.9514215	0.0000000	11.4576284
C	-7.8817021	0.0000000	10.6301155
C	0.0000000	4.8698930	14.1220381
C	0.0000000	6.1098311	14.2207048
C	0.0000000	7.4595663	14.2838279

C	0.0000000	8.7036569	14.2949012
C	4.7577692	0.0000000	12.8393072
C	5.8466161	0.0000000	12.2356106
C	6.9514215	0.0000000	11.4576284
C	7.8817021	0.0000000	10.6301155
C	12.0848546	0.0000000	1.9156999
C	12.1937921	0.0000000	0.6756921
C	12.1937921	0.0000000	-0.6756921
C	12.0848546	0.0000000	-1.9156999
C	-12.0848546	0.0000000	1.9156999
C	-12.1937921	0.0000000	0.6756921
C	-12.1937921	0.0000000	-0.6756921
C	-12.0848546	0.0000000	-1.9156999
C	0.0000000	4.8698930	-14.1220381
C	0.0000000	6.1098311	-14.2207048
C	0.0000000	7.4595663	-14.2838279
C	0.0000000	8.7036569	-14.2949012
C	-4.7577692	0.0000000	-12.8393072
C	-5.8466161	0.0000000	-12.2356106
C	-6.9514215	0.0000000	-11.4576284
C	-7.8817021	0.0000000	-10.6301155
C	0.0000000	-4.8698930	-14.1220381
C	0.0000000	-6.1098311	-14.2207048
C	0.0000000	-7.4595663	-14.2838279
C	0.0000000	-8.7036569	-14.2949012
C	4.7577692	0.0000000	-12.8393072
C	5.8466161	0.0000000	-12.2356106
C	6.9514215	0.0000000	-11.4576284
C	7.8817021	0.0000000	-10.6301155
C	0.0000000	-18.2516650	12.3094722
C	0.0000000	-19.3531525	11.7300736
C	0.0000000	-20.4917023	11.0024080
C	0.0000000	-21.4778853	10.2429686
C	0.0000000	18.2516650	12.3094722
C	0.0000000	19.3531525	11.7300736
C	0.0000000	20.4917023	11.0024080
C	0.0000000	21.4778853	10.2429686
C	0.0000000	-18.2516650	-12.3094722
C	0.0000000	-19.3531525	-11.7300736
C	0.0000000	-20.4917023	-11.0024080
C	0.0000000	-21.4778853	-10.2429686
C	0.0000000	18.2516650	-12.3094722
C	0.0000000	19.3531525	-11.7300736
C	0.0000000	20.4917023	-11.0024080
C	0.0000000	21.4778853	-10.2429686
C	0.0000000	-26.3543467	1.9130136
C	0.0000000	-26.4911860	0.6755646
C	0.0000000	-26.4911860	-0.6755646
C	0.0000000	-26.3543467	-1.9130136

```
C     0.0000000   26.3543467    1.9130136
C     0.0000000   26.4911860    0.6755646
C     0.0000000   26.4911860   -0.6755646
C     0.0000000   26.3543467   -1.9130136
```

Molecule 4)

```
550
Energy =
Zn    0.0000000   -0.0000000   16.4217156
C    -0.6884634   -4.2784288   16.3308118
C     0.6884634   -4.2784288   16.3308118
C     1.1020721   -2.8937283   16.3731942
C    -1.1020721   -2.8937283   16.3731942
N     0.0000000   -2.0664911   16.4135191
C    -2.4612961   -2.4612961   16.3264672
C    -2.8937283   -1.1020721   16.3731942
C    -4.2784288   -0.6884634   16.3308118
C    -4.2784288    0.6884634   16.3308118
C    -2.8937283    1.1020721   16.3731942
N    -2.0664911    0.0000000   16.4135191
C    -2.4612961    2.4612961   16.3264672
C    -1.1020721    2.8937283   16.3731942
C    -0.6884634    4.2784288   16.3308118
C     0.6884634    4.2784288   16.3308118
C     1.1020721    2.8937283   16.3731942
N     0.0000000    2.0664911   16.4135191
C     2.4612961    2.4612961   16.3264672
C     2.8937283    1.1020721   16.3731942
C     4.2784288    0.6884634   16.3308118
C     4.2784288   -0.6884634   16.3308118
C     2.8937283   -1.1020721   16.3731942
N     2.0664911    0.0000000   16.4135191
C     2.4612961   -2.4612961   16.3264672
H    -1.3690977   -5.1356912   16.3005527
H     1.3690977   -5.1356912   16.3005527
H    -5.1356912   -1.3690977   16.3005527
H    -5.1356912    1.3690977   16.3005527
H    -1.3690977    5.1356912   16.3005527
H     1.3690977    5.1356912   16.3005527
H     5.1356912    1.3690977   16.3005527
H     5.1356912   -1.3690977   16.3005527
Zn   12.5940908  -12.5940908    0.0000000
C    10.7053193  -14.3067144    3.5007313
C    10.1292642  -15.0894580    2.5251668
C    10.6637600  -14.6367599    1.2564844
C    11.6071948  -13.3963002    2.8229648
N    11.5714295  -13.6138105    1.4683947
C    12.4153559  -12.4153559    3.4781166
C    13.3963002  -11.6071948    2.8229648
```

C	14.3067144	-10.7053193	3.5007313
C	15.0894580	-10.1292642	2.5251668
C	14.6367599	-10.6637600	1.2564844
N	13.6138105	-11.5714295	1.4683947
C	15.1196020	-10.2700158	0.0000000
C	14.6367599	-10.6637600	-1.2564844
C	15.0894580	-10.1292642	-2.5251668
C	14.3067144	-10.7053193	-3.5007313
C	13.3963002	-11.6071948	-2.8229648
N	13.6138105	-11.5714295	-1.4683947
C	12.4153559	-12.4153559	-3.4781166
C	11.6071948	-13.3963002	-2.8229648
C	10.7053193	-14.3067144	-3.5007313
C	10.1292642	-15.0894580	-2.5251668
C	10.6637600	-14.6367599	-1.2564844
N	11.5714295	-13.6138105	-1.4683947
C	10.2700158	-15.1196020	0.0000000
H	10.5469382	-14.3398593	4.5840464
H	9.3962181	-15.8953760	2.6495927
H	14.3398593	-10.5469382	4.5840464
H	15.8953760	-9.3962181	2.6495927
H	15.8953760	-9.3962181	-2.6495927
H	14.3398593	-10.5469382	-4.5840464
H	10.5469382	-14.3398593	-4.5840464
H	9.3962181	-15.8953760	-2.6495927
Zn	-12.5940908	12.5940908	0.0000000
C	-10.7053193	14.3067144	3.5007313
C	-10.1292642	15.0894580	2.5251668
C	-10.6637600	14.6367599	1.2564844
C	-11.6071948	13.3963002	2.8229648
N	-11.5714295	13.6138105	1.4683947
C	-12.4153559	12.4153559	3.4781166
C	-13.3963002	11.6071948	2.8229648
C	-14.3067144	10.7053193	3.5007313
C	-15.0894580	10.1292642	2.5251668
C	-14.6367599	10.6637600	1.2564844
N	-13.6138105	11.5714295	1.4683947
C	-15.1196020	10.2700158	0.0000000
C	-14.6367599	10.6637600	-1.2564844
C	-15.0894580	10.1292642	-2.5251668
C	-14.3067144	10.7053193	-3.5007313
C	-13.3963002	11.6071948	-2.8229648
N	-13.6138105	11.5714295	-1.4683947
C	-12.4153559	12.4153559	-3.4781166
C	-11.6071948	13.3963002	-2.8229648
C	-10.7053193	14.3067144	-3.5007313
C	-10.1292642	15.0894580	-2.5251668
C	-10.6637600	14.6367599	-1.2564844
N	-11.5714295	13.6138105	-1.4683947

C	-10.2700158	15.1196020	0.0000000
H	-10.5469382	14.3398593	4.5840464
H	-9.3962181	15.8953760	2.6495927
H	-14.3398593	10.5469382	4.5840464
H	-15.8953760	9.3962181	2.6495927
H	-15.8953760	9.3962181	-2.6495927
H	-14.3398593	10.5469382	-4.5840464
H	-10.5469382	14.3398593	-4.5840464
H	-9.3962181	15.8953760	-2.6495927
Zn	0.0000000	0.0000000	-16.4217156
C	4.2784288	0.6884634	-16.3308118
C	4.2784288	-0.6884634	-16.3308118
C	2.8937283	-1.1020721	-16.3731942
C	2.8937283	1.1020721	-16.3731942
N	2.0664911	0.0000000	-16.4135191
C	2.4612961	2.4612961	-16.3264672
C	1.1020721	2.8937283	-16.3731942
C	0.6884634	4.2784288	-16.3308118
C	-0.6884634	4.2784288	-16.3308118
C	-1.1020721	2.8937283	-16.3731942
N	0.0000000	2.0664911	-16.4135191
C	-2.4612961	2.4612961	-16.3264672
C	-2.8937283	1.1020721	-16.3731942
C	-4.2784288	0.6884634	-16.3308118
C	-4.2784288	-0.6884634	-16.3308118
C	-2.8937283	-1.1020721	-16.3731942
N	-2.0664911	0.0000000	-16.4135191
C	-2.4612961	-2.4612961	-16.3264672
C	-1.1020721	-2.8937283	-16.3731942
C	-0.6884634	-4.2784288	-16.3308118
C	0.6884634	-4.2784288	-16.3308118
C	1.1020721	-2.8937283	-16.3731942
N	0.0000000	-2.0664911	-16.4135191
C	2.4612961	-2.4612961	-16.3264672
H	5.1356912	1.3690977	-16.3005527
H	5.1356912	-1.3690977	-16.3005527
H	1.3690977	5.1356912	-16.3005527
H	-1.3690977	5.1356912	-16.3005527
H	-5.1356912	1.3690977	-16.3005527
H	-5.1356912	-1.3690977	-16.3005527
H	-1.3690977	-5.1356912	-16.3005527
H	1.3690977	-5.1356912	-16.3005527
Zn	8.8209240	-8.8209240	12.0379516
C	7.6724780	-12.6326697	10.3554438
C	8.7936881	-12.3950035	9.5920555
C	9.3423039	-11.1313992	10.0433777
C	7.5294922	-11.5023823	11.2510124
N	8.5672622	-10.6096752	11.0487527
C	6.4630918	-11.3128240	12.1423057

C	6.2142380	-10.1876090	12.9411387
C	5.0294960	-9.9900310	13.7522277
C	5.1296990	-8.7314560	14.3016380
C	6.3879114	-8.1771550	13.8413723
N	7.0304366	-9.0729215	13.0241868
C	6.8825617	-6.8825617	14.1931750
C	8.1771550	-6.3879114	13.8413723
C	8.7314560	-5.1296990	14.3016380
C	9.9900310	-5.0294960	13.7522277
C	10.1876090	-6.2142380	12.9411387
N	9.0729215	-7.0304366	13.0241868
C	11.3128240	-6.4630918	12.1423057
C	11.5023823	-7.5294922	11.2510124
C	12.6326697	-7.6724780	10.3554438
C	12.3950035	-8.7936881	9.5920555
C	11.1313992	-9.3423039	10.0433777
N	10.6096752	-8.5672622	11.0487527
C	10.5221400	-10.5221400	9.5129551
H	6.9923323	-13.4915230	10.3096379
H	9.2231656	-13.0159824	8.7985436
H	4.2190454	-10.7185879	13.8734746
H	4.4253748	-8.2187196	14.9655471
H	8.2187196	-4.4253748	14.9655471
H	10.7185879	-4.2190454	13.8734746
H	13.4915230	-6.9923323	10.3096379
H	13.0159824	-9.2231656	8.7985436
Zn	-8.8209240	8.8209240	12.0379516
C	-5.1296990	8.7314560	14.3016380
C	-5.0294960	9.9900310	13.7522277
C	-6.2142380	10.1876090	12.9411387
C	-6.3879114	8.1771550	13.8413723
N	-7.0304366	9.0729215	13.0241868
C	-6.8825617	6.8825617	14.1931750
C	-8.1771550	6.3879114	13.8413723
C	-8.7314560	5.1296990	14.3016380
C	-9.9900310	5.0294960	13.7522277
C	-10.1876090	6.2142380	12.9411387
N	-9.0729215	7.0304366	13.0241868
C	-11.3128240	6.4630918	12.1423057
C	-11.5023823	7.5294922	11.2510124
C	-12.6326697	7.6724780	10.3554438
C	-12.3950035	8.7936881	9.5920555
C	-11.1313992	9.3423039	10.0433777
N	-10.6096752	8.5672622	11.0487527
C	-10.5221400	10.5221400	9.5129551
C	-9.3423039	11.1313992	10.0433777
C	-8.7936881	12.3950035	9.5920555
C	-7.6724780	12.6326697	10.3554438
C	-7.5294922	11.5023823	11.2510124

N	-8.5672622	10.6096752	11.0487527
C	-6.4630918	11.3128240	12.1423057
H	-4.4253748	8.2187196	14.9655471
H	-4.2190454	10.7185879	13.8734746
H	-8.2187196	4.4253748	14.9655471
H	-10.7185879	4.2190454	13.8734746
H	-13.4915230	6.9923323	10.3096379
H	-13.0159824	9.2231656	8.7985436
H	-9.2231656	13.0159824	8.7985436
H	-6.9923323	13.4915230	10.3096379
Zn	-8.8209240	8.8209240	-12.0379516
C	-5.0294960	9.9900310	-13.7522277
C	-5.1296990	8.7314560	-14.3016380
C	-6.3879114	8.1771550	-13.8413723
C	-6.2142380	10.1876090	-12.9411387
N	-7.0304366	9.0729215	-13.0241868
C	-6.4630918	11.3128240	-12.1423057
C	-7.5294922	11.5023823	-11.2510124
C	-7.6724780	12.6326697	-10.3554438
C	-8.7936881	12.3950035	-9.5920555
C	-9.3423039	11.1313992	-10.0433777
N	-8.5672622	10.6096752	-11.0487527
C	-10.5221400	10.5221400	-9.5129551
C	-11.1313992	9.3423039	-10.0433777
C	-12.3950035	8.7936881	-9.5920555
C	-12.6326697	7.6724780	-10.3554438
C	-11.5023823	7.5294922	-11.2510124
N	-10.6096752	8.5672622	-11.0487527
C	-11.3128240	6.4630918	-12.1423057
C	-10.1876090	6.2142380	-12.9411387
C	-9.9900310	5.0294960	-13.7522277
C	-8.7314560	5.1296990	-14.3016380
C	-8.1771550	6.3879114	-13.8413723
N	-9.0729215	7.0304366	-13.0241868
C	-6.8825617	6.8825617	-14.1931750
H	-4.2190454	10.7185879	-13.8734746
H	-4.4253748	8.2187196	-14.9655471
H	-6.9923323	13.4915230	-10.3096379
H	-9.2231656	13.0159824	-8.7985436
H	-13.0159824	9.2231656	-8.7985436
H	-13.4915230	6.9923323	-10.3096379
H	-10.7185879	4.2190454	-13.8734746
H	-8.2187196	4.4253748	-14.9655471
Zn	8.8209240	-8.8209240	-12.0379516
C	8.7936881	-12.3950035	-9.5920555
C	7.6724780	-12.6326697	-10.3554438
C	7.5294922	-11.5023823	-11.2510124
C	9.3423039	-11.1313992	-10.0433777
N	8.5672622	-10.6096752	-11.0487527

C	10.5221400	-10.5221400	-9.5129551
C	11.1313992	-9.3423039	-10.0433777
C	12.3950035	-8.7936881	-9.5920555
C	12.6326697	-7.6724780	-10.3554438
C	11.5023823	-7.5294922	-11.2510124
N	10.6096752	-8.5672622	-11.0487527
C	11.3128240	-6.4630918	-12.1423057
C	10.1876090	-6.2142380	-12.9411387
C	9.9900310	-5.0294960	-13.7522277
C	8.7314560	-5.1296990	-14.3016380
C	8.1771550	-6.3879114	-13.8413723
N	9.0729215	-7.0304366	-13.0241868
C	6.8825617	-6.8825617	-14.1931750
C	6.3879114	-8.1771550	-13.8413723
C	5.1296990	-8.7314560	-14.3016380
C	5.0294960	-9.9900310	-13.7522277
C	6.2142380	-10.1876090	-12.9411387
N	7.0304366	-9.0729215	-13.0241868
C	6.4630918	-11.3128240	-12.1423057
H	9.2231656	-13.0159824	-8.7985436
H	6.9923323	-13.4915230	-10.3096379
H	13.0159824	-9.2231656	-8.7985436
H	13.4915230	-6.9923323	-10.3096379
H	10.7185879	-4.2190454	-13.8734746
H	8.2187196	-4.4253748	-14.9655471
H	4.4253748	-8.2187196	-14.9655471
H	4.2190454	-10.7185879	-13.8734746
Zn	12.5940908	12.5940908	0.0000000
C	14.3067144	10.7053193	3.5007313
C	15.0894580	10.1292642	2.5251668
C	14.6367599	10.6637600	1.2564844
C	13.3963002	11.6071948	2.8229648
N	13.6138105	11.5714295	1.4683947
C	12.4153559	12.4153559	3.4781166
C	11.6071948	13.3963002	2.8229648
C	10.7053193	14.3067144	3.5007313
C	10.1292642	15.0894580	2.5251668
C	10.6637600	14.6367599	1.2564844
N	11.5714295	13.6138105	1.4683947
C	10.2700158	15.1196020	0.0000000
C	10.6637600	14.6367599	-1.2564844
C	10.1292642	15.0894580	-2.5251668
C	10.7053193	14.3067144	-3.5007313
C	11.6071948	13.3963002	-2.8229648
N	11.5714295	13.6138105	-1.4683947
C	12.4153559	12.4153559	-3.4781166
C	13.3963002	11.6071948	-2.8229648
C	14.3067144	10.7053193	-3.5007313
C	15.0894580	10.1292642	-2.5251668

C	14.6367599	10.6637600	-1.2564844
N	13.6138105	11.5714295	-1.4683947
C	15.1196020	10.2700158	0.0000000
H	14.3398593	10.5469382	4.5840464
H	15.8953760	9.3962181	2.6495927
H	10.5469382	14.3398593	4.5840464
H	9.3962181	15.8953760	2.6495927
H	9.3962181	15.8953760	-2.6495927
H	10.5469382	14.3398593	-4.5840464
H	14.3398593	10.5469382	-4.5840464
H	15.8953760	9.3962181	-2.6495927
Zn	-12.5940908	-12.5940908	0.0000000
C	-14.3067144	-10.7053193	-3.5007313
C	-15.0894580	-10.1292642	-2.5251668
C	-14.6367599	-10.6637600	-1.2564844
C	-13.3963002	-11.6071948	-2.8229648
N	-13.6138105	-11.5714295	-1.4683947
C	-12.4153559	-12.4153559	-3.4781166
C	-11.6071948	-13.3963002	-2.8229648
C	-10.7053193	-14.3067144	-3.5007313
C	-10.1292642	-15.0894580	-2.5251668
C	-10.6637600	-14.6367599	-1.2564844
N	-11.5714295	-13.6138105	-1.4683947
C	-10.2700158	-15.1196020	0.0000000
C	-10.6637600	-14.6367599	1.2564844
C	-10.1292642	-15.0894580	2.5251668
C	-10.7053193	-14.3067144	3.5007313
C	-11.6071948	-13.3963002	2.8229648
N	-11.5714295	-13.6138105	1.4683947
C	-12.4153559	-12.4153559	3.4781166
C	-13.3963002	-11.6071948	2.8229648
C	-14.3067144	-10.7053193	3.5007313
C	-15.0894580	-10.1292642	2.5251668
C	-14.6367599	-10.6637600	1.2564844
N	-13.6138105	-11.5714295	1.4683947
C	-15.1196020	-10.2700158	0.0000000
H	-14.3398593	-10.5469382	-4.5840464
H	-15.8953760	-9.3962181	-2.6495927
H	-10.5469382	-14.3398593	-4.5840464
H	-9.3962181	-15.8953760	-2.6495927
H	-9.3962181	-15.8953760	2.6495927
H	-10.5469382	-14.3398593	4.5840464
H	-14.3398593	-10.5469382	4.5840464
H	-15.8953760	-9.3962181	2.6495927
Zn	8.8209240	8.8209240	12.0379516
C	12.6326697	7.6724780	10.3554438
C	12.3950035	8.7936881	9.5920555
C	11.1313992	9.3423039	10.0433777
C	11.5023823	7.5294922	11.2510124

N	10.6096752	8.5672622	11.0487527
C	11.3128240	6.4630918	12.1423057
C	10.1876090	6.2142380	12.9411387
C	9.9900310	5.0294960	13.7522277
C	8.7314560	5.1296990	14.3016380
C	8.1771550	6.3879114	13.8413723
N	9.0729215	7.0304366	13.0241868
C	6.8825617	6.8825617	14.1931750
C	6.3879114	8.1771550	13.8413723
C	5.1296990	8.7314560	14.3016380
C	5.0294960	9.9900310	13.7522277
C	6.2142380	10.1876090	12.9411387
N	7.0304366	9.0729215	13.0241868
C	6.4630918	11.3128240	12.1423057
C	7.5294922	11.5023823	11.2510124
C	7.6724780	12.6326697	10.3554438
C	8.7936881	12.3950035	9.5920555
C	9.3423039	11.1313992	10.0433777
N	8.5672622	10.6096752	11.0487527
C	10.5221400	10.5221400	9.5129551
H	13.4915230	6.9923323	10.3096379
H	13.0159824	9.2231656	8.7985436
H	10.7185879	4.2190454	13.8734746
H	8.2187196	4.4253748	14.9655471
H	4.4253748	8.2187196	14.9655471
H	4.2190454	10.7185879	13.8734746
H	6.9923323	13.4915230	10.3096379
H	9.2231656	13.0159824	8.7985436
Zn	-8.8209240	-8.8209240	12.0379516
C	-8.7936881	-12.3950035	9.5920555
C	-7.6724780	-12.6326697	10.3554438
C	-7.5294922	-11.5023823	11.2510124
C	-9.3423039	-11.1313992	10.0433777
N	-8.5672622	-10.6096752	11.0487527
C	-10.5221400	-10.5221400	9.5129551
C	-11.1313992	-9.3423039	10.0433777
C	-12.3950035	-8.7936881	9.5920555
C	-12.6326697	-7.6724780	10.3554438
C	-11.5023823	-7.5294922	11.2510124
N	-10.6096752	-8.5672622	11.0487527
C	-11.3128240	-6.4630918	12.1423057
C	-10.1876090	-6.2142380	12.9411387
C	-9.9900310	-5.0294960	13.7522277
C	-8.7314560	-5.1296990	14.3016380
C	-8.1771550	-6.3879114	13.8413723
N	-9.0729215	-7.0304366	13.0241868
C	-6.8825617	-6.8825617	14.1931750
C	-6.3879114	-8.1771550	13.8413723
C	-5.1296990	-8.7314560	14.3016380

C	-5.0294960	-9.9900310	13.7522277
C	-6.2142380	-10.1876090	12.9411387
N	-7.0304366	-9.0729215	13.0241868
C	-6.4630918	-11.3128240	12.1423057
H	-9.2231656	-13.0159824	8.7985436
H	-6.9923323	-13.4915230	10.3096379
H	-13.0159824	-9.2231656	8.7985436
H	-13.4915230	-6.9923323	10.3096379
H	-10.7185879	-4.2190454	13.8734746
H	-8.2187196	-4.4253748	14.9655471
H	-4.4253748	-8.2187196	14.9655471
H	-4.2190454	-10.7185879	13.8734746
Zn	-8.8209240	-8.8209240	-12.0379516
C	-7.6724780	-12.6326697	-10.3554438
C	-8.7936881	-12.3950035	-9.5920555
C	-9.3423039	-11.1313992	-10.0433777
C	-7.5294922	-11.5023823	-11.2510124
N	-8.5672622	-10.6096752	-11.0487527
C	-6.4630918	-11.3128240	-12.1423057
C	-6.2142380	-10.1876090	-12.9411387
C	-5.0294960	-9.9900310	-13.7522277
C	-5.1296990	-8.7314560	-14.3016380
C	-6.3879114	-8.1771550	-13.8413723
N	-7.0304366	-9.0729215	-13.0241868
C	-6.8825617	-6.8825617	-14.1931750
C	-8.1771550	-6.3879114	-13.8413723
C	-8.7314560	-5.1296990	-14.3016380
C	-9.9900310	-5.0294960	-13.7522277
C	-10.1876090	-6.2142380	-12.9411387
N	-9.0729215	-7.0304366	-13.0241868
C	-11.3128240	-6.4630918	-12.1423057
C	-11.5023823	-7.5294922	-11.2510124
C	-12.6326697	-7.6724780	-10.3554438
C	-12.3950035	-8.7936881	-9.5920555
C	-11.1313992	-9.3423039	-10.0433777
N	-10.6096752	-8.5672622	-11.0487527
C	-10.5221400	-10.5221400	-9.5129551
H	-6.9923323	-13.4915230	-10.3096379
H	-9.2231656	-13.0159824	-8.7985436
H	-4.2190454	-10.7185879	-13.8734746
H	-4.4253748	-8.2187196	-14.9655471
H	-8.2187196	-4.4253748	-14.9655471
H	-10.7185879	-4.2190454	-13.8734746
H	-13.4915230	-6.9923323	-10.3096379
H	-13.0159824	-9.2231656	-8.7985436
Zn	8.8209240	8.8209240	-12.0379516
C	9.9900310	5.0294960	-13.7522277
C	8.7314560	5.1296990	-14.3016380
C	8.1771550	6.3879114	-13.8413723

C	10.1876090	6.2142380	-12.9411387
N	9.0729215	7.0304366	-13.0241868
C	11.3128240	6.4630918	-12.1423057
C	11.5023823	7.5294922	-11.2510124
C	12.6326697	7.6724780	-10.3554438
C	12.3950035	8.7936881	-9.5920555
C	11.1313992	9.3423039	-10.0433777
N	10.6096752	8.5672622	-11.0487527
C	10.5221400	10.5221400	-9.5129551
C	9.3423039	11.1313992	-10.0433777
C	8.7936881	12.3950035	-9.5920555
C	7.6724780	12.6326697	-10.3554438
C	7.5294922	11.5023823	-11.2510124
N	8.5672622	10.6096752	-11.0487527
C	6.4630918	11.3128240	-12.1423057
C	6.2142380	10.1876090	-12.9411387
C	5.0294960	9.9900310	-13.7522277
C	5.1296990	8.7314560	-14.3016380
C	6.3879114	8.1771550	-13.8413723
N	7.0304366	9.0729215	-13.0241868
C	6.8825617	6.8825617	-14.1931750
H	10.7185879	4.2190454	-13.8734746
H	8.2187196	4.4253748	-14.9655471
H	13.4915230	6.9923323	-10.3096379
H	13.0159824	9.2231656	-8.7985436
H	9.2231656	13.0159824	-8.7985436
H	6.9923323	13.4915230	-10.3096379
H	4.2190454	10.7185879	-13.8734746
H	4.4253748	8.2187196	-14.9655471
C	-3.4494145	-3.4494145	16.1225869
C	-4.3024150	-4.3024150	15.8183904
C	-5.2074537	-5.2074537	15.3845026
C	-6.0139239	-6.0139239	14.8864861
C	-3.4494145	3.4494145	16.1225869
C	-4.3024150	4.3024150	15.8183904
C	-5.2074537	5.2074537	15.3845026
C	-6.0139239	6.0139239	14.8864861
C	3.4494145	3.4494145	16.1225869
C	4.3024150	4.3024150	15.8183904
C	5.2074537	5.2074537	15.3845026
C	6.0139239	6.0139239	14.8864861
C	3.4494145	-3.4494145	16.1225869
C	4.3024150	-4.3024150	15.8183904
C	5.2074537	-5.2074537	15.3845026
C	6.0139239	-6.0139239	14.8864861
C	12.1904626	-12.1904626	4.8523336
C	11.9024419	-11.9024419	6.0284617
C	11.5147429	-11.5147429	7.2635469
C	11.0915552	-11.0915552	8.3548603

C	12.1904626	-12.1904626	-4.8523336
C	11.9024419	-11.9024419	-6.0284617
C	11.5147429	-11.5147429	-7.2635469
C	11.0915552	-11.0915552	-8.3548603
C	-12.1904626	12.1904626	4.8523336
C	-11.9024419	11.9024419	6.0284617
C	-11.5147429	11.5147429	7.2635469
C	-11.0915552	11.0915552	8.3548603
C	-12.1904626	12.1904626	-4.8523336
C	-11.9024419	11.9024419	-6.0284617
C	-11.5147429	11.5147429	-7.2635469
C	-11.0915552	11.0915552	-8.3548603
C	3.4494145	3.4494145	-16.1225869
C	4.3024150	4.3024150	-15.8183904
C	5.2074537	5.2074537	-15.3845026
C	6.0139239	6.0139239	-14.8864861
C	-3.4494145	3.4494145	-16.1225869
C	-4.3024150	4.3024150	-15.8183904
C	-5.2074537	5.2074537	-15.3845026
C	-6.0139239	6.0139239	-14.8864861
C	-3.4494145	-3.4494145	-16.1225869
C	-4.3024150	-4.3024150	-15.8183904
C	-5.2074537	-5.2074537	-15.3845026
C	-6.0139239	-6.0139239	-14.8864861
C	3.4494145	-3.4494145	-16.1225869
C	4.3024150	-4.3024150	-15.8183904
C	5.2074537	-5.2074537	-15.3845026
C	6.0139239	-6.0139239	-14.8864861
C	12.1904626	12.1904626	4.8523336
C	11.9024419	11.9024419	6.0284617
C	11.5147429	11.5147429	7.2635469
C	11.0915552	11.0915552	8.3548603
C	12.1904626	12.1904626	-4.8523336
C	11.9024419	11.9024419	-6.0284617
C	11.5147429	11.5147429	-7.2635469
C	11.0915552	11.0915552	-8.3548603
C	-12.1904626	-12.1904626	-4.8523336
C	-11.9024419	-11.9024419	-6.0284617
C	-11.5147429	-11.5147429	-7.2635469
C	-11.0915552	-11.0915552	-8.3548603
C	-12.1904626	-12.1904626	4.8523336
C	-11.9024419	-11.9024419	6.0284617
C	-11.5147429	-11.5147429	7.2635469
C	-11.0915552	-11.0915552	8.3548603
H	15.9351200	-9.5317372	0.0000000
H	9.5317372	-15.9351200	0.0000000
H	-15.9351200	9.5317372	0.0000000
H	-9.5317372	15.9351200	0.0000000
H	5.7119398	-12.1154930	12.1821415

H	12.1154930	-5.7119398	12.1821415
H	-12.1154930	5.7119398	12.1821415
H	-5.7119398	12.1154930	12.1821415
H	-5.7119398	12.1154930	-12.1821415
H	-12.1154930	5.7119398	-12.1821415
H	12.1154930	-5.7119398	-12.1821415
H	5.7119398	-12.1154930	-12.1821415
H	9.5317372	15.9351200	0.0000000
H	15.9351200	9.5317372	0.0000000
H	-9.5317372	-15.9351200	0.0000000
H	-15.9351200	-9.5317372	0.0000000
H	12.1154930	5.7119398	12.1821415
H	5.7119398	12.1154930	12.1821415
H	-12.1154930	-5.7119398	12.1821415
H	-5.7119398	-12.1154930	12.1821415
H	-5.7119398	-12.1154930	-12.1821415
H	-12.1154930	-5.7119398	-12.1821415
H	12.1154930	5.7119398	-12.1821415
H	5.7119398	12.1154930	-12.1821415

Molecule 5

628

Energy =

Zn	17.9607212	0.0000000	0.0000000
C	17.8170663	-3.5053242	-2.5379167
C	17.9401633	-2.5378871	-3.5104916
C	18.0059773	-1.2667961	-2.8241528
C	17.8285078	-2.8171902	-1.2666355
N	17.9530981	-1.4584165	-1.4601336
C	17.7096559	-3.4647662	0.0000000
C	17.8285078	-2.8171902	1.2666355
C	17.8170663	-3.5053242	2.5379167
C	17.9401633	-2.5378871	3.5104916
C	18.0059773	-1.2667961	2.8241528
N	17.9530981	-1.4584165	1.4601336
C	18.0455670	0.0000000	3.4812319
C	18.0059773	1.2667961	2.8241528
C	17.9401633	2.5378871	3.5104916
C	17.8170663	3.5053242	2.5379167
C	17.8285078	2.8171902	1.2666355
N	17.9530981	1.4584165	1.4601336
C	17.7096559	3.4647662	0.0000000
C	17.8285078	2.8171902	-1.2666355
C	17.8170663	3.5053242	-2.5379167
C	17.9401633	2.5378871	-3.5104916
C	18.0059773	1.2667961	-2.8241528
N	17.9530981	1.4584165	-1.4601336
C	18.0455670	0.0000000	-3.4812319
H	17.7309584	-4.5897964	-2.6635919

H	17.9710465	-2.6662632	-4.5975044
H	17.7309584	-4.5897964	2.6635919
H	17.9710465	-2.6662632	4.5975044
H	17.9710465	2.6662632	4.5975044
H	17.7309584	4.5897964	2.6635919
H	17.7309584	4.5897964	-2.6635919
H	17.9710465	2.6662632	-4.5975044
Zn	-17.9607212	0.0000000	0.0000000
C	-17.8170663	-3.5053242	2.5379167
C	-17.9401633	-2.5378871	3.5104916
C	-18.0059773	-1.2667961	2.8241528
C	-17.8285078	-2.8171902	1.2666355
N	-17.9530981	-1.4584165	1.4601336
C	-17.7096559	-3.4647662	0.0000000
C	-17.8285078	-2.8171902	-1.2666355
C	-17.8170663	-3.5053242	-2.5379167
C	-17.9401633	-2.5378871	-3.5104916
C	-18.0059773	-1.2667961	-2.8241528
N	-17.9530981	-1.4584165	-1.4601336
C	-18.0455670	0.0000000	-3.4812319
C	-18.0059773	1.2667961	-2.8241528
C	-17.9401633	2.5378871	-3.5104916
C	-17.8170663	3.5053242	-2.5379167
C	-17.8285078	2.8171902	-1.2666355
N	-17.9530981	1.4584165	-1.4601336
C	-17.7096559	3.4647662	0.0000000
C	-17.8285078	2.8171902	1.2666355
C	-17.8170663	3.5053242	2.5379167
C	-17.9401633	2.5378871	3.5104916
C	-18.0059773	1.2667961	2.8241528
N	-17.9530981	1.4584165	1.4601336
C	-18.0455670	0.0000000	3.4812319
H	-17.7309584	-4.5897964	2.6635919
H	-17.9710465	-2.6662632	4.5975044
H	-17.7309584	-4.5897964	-2.6635919
H	-17.9710465	-2.6662632	-4.5975044
H	-17.9710465	2.6662632	-4.5975044
H	-17.7309584	4.5897964	-2.6635919
H	-17.7309584	4.5897964	2.6635919
H	-17.9710465	2.6662632	4.5975044
Zn	0.0000000	16.5418794	0.0000000
C	2.5254933	16.5616449	3.5084333
C	3.5032224	16.4379561	2.5468859
C	2.8259681	16.4323192	1.2650565
C	1.2565830	16.6100627	2.8101754
N	1.4695140	16.5407267	1.4444589
C	0.0000000	16.6635044	3.4303741
C	-1.2565830	16.6100627	2.8101754
C	-2.5254933	16.5616449	3.5084333

C	-3.5032224	16.4379561	2.5468859
C	-2.8259681	16.4323192	1.2650565
N	-1.4695140	16.5407267	1.4444589
C	-3.4841356	16.3292699	0.0000000
C	-2.8259681	16.4323192	-1.2650565
C	-3.5032224	16.4379561	-2.5468859
C	-2.5254933	16.5616449	-3.5084333
C	-1.2565830	16.6100627	-2.8101754
N	-1.4695140	16.5407267	-1.4444589
C	0.0000000	16.6635044	-3.4303741
C	1.2565830	16.6100627	-2.8101754
C	2.5254933	16.5616449	-3.5084333
C	3.5032224	16.4379561	-2.5468859
C	2.8259681	16.4323192	-1.2650565
N	1.4695140	16.5407267	-1.4444589
C	3.4841356	16.3292699	0.0000000
H	2.6486538	16.6055688	4.5971229
H	4.5875881	16.3632376	2.6819671
H	-2.6486538	16.6055688	4.5971229
H	-4.5875881	16.3632376	2.6819671
H	-4.5875881	16.3632376	-2.6819671
H	-2.6486538	16.6055688	-4.5971229
H	2.6486538	16.6055688	-4.5971229
H	4.5875881	16.3632376	-2.6819671
Zn	0.0000000	-16.5418794	0.0000000
C	2.5254933	-16.5616449	-3.5084333
C	3.5032224	-16.4379561	-2.5468859
C	2.8259681	-16.4323192	-1.2650565
C	1.2565830	-16.6100627	-2.8101754
N	1.4695140	-16.5407267	-1.4444589
C	0.0000000	-16.6635044	-3.4303741
C	-1.2565830	-16.6100627	-2.8101754
C	-2.5254933	-16.5616449	-3.5084333
C	-3.5032224	-16.4379561	-2.5468859
C	-2.8259681	-16.4323192	-1.2650565
N	-1.4695140	-16.5407267	-1.4444589
C	-3.4841356	-16.3292699	0.0000000
C	-2.8259681	-16.4323192	1.2650565
C	-3.5032224	-16.4379561	2.5468859
C	-2.5254933	-16.5616449	3.5084333
C	-1.2565830	-16.6100627	2.8101754
N	-1.4695140	-16.5407267	1.4444589
C	0.0000000	-16.6635044	3.4303741
C	1.2565830	-16.6100627	2.8101754
C	2.5254933	-16.5616449	3.5084333
C	3.5032224	-16.4379561	2.5468859
C	2.8259681	-16.4323192	1.2650565
N	1.4695140	-16.5407267	1.4444589
C	3.4841356	-16.3292699	0.0000000

H	2.6486538	-16.6055688	-4.5971229
H	4.5875881	-16.3632376	-2.6819671
H	-2.6486538	-16.6055688	-4.5971229
H	-4.5875881	-16.3632376	-2.6819671
H	-4.5875881	-16.3632376	2.6819671
H	-2.6486538	-16.6055688	4.5971229
H	2.6486538	-16.6055688	4.5971229
H	4.5875881	-16.3632376	2.6819671
Zn	12.4636134	11.9220136	0.0000000
C	15.0132942	9.5189177	-2.5469242
C	14.3714229	10.2672360	-3.5080509
C	13.4557166	11.1473707	-2.8099758
C	14.5017342	9.9635208	-1.2651974
N	13.5650456	10.9502156	-1.4443573
C	14.9178905	9.4434985	0.0000000
C	14.5017342	9.9635208	1.2651974
C	15.0132942	9.5189177	2.5469242
C	14.3714229	10.2672360	3.5080509
C	13.4557166	11.1473707	2.8099758
N	13.5650456	10.9502156	1.4443573
C	12.5517491	12.0218102	3.4297684
C	11.5697030	12.8082335	2.8095897
C	10.5823243	13.6066541	3.5078284
C	9.7596598	14.1503272	2.5466670
C	10.2640236	13.6980268	1.2650671
N	11.3600446	12.8914562	1.4443377
C	9.6967282	14.0475890	0.0000000
C	10.2640236	13.6980268	-1.2650671
C	9.7596598	14.1503272	-2.5466670
C	10.5823243	13.6066541	-3.5078284
C	11.5697030	12.8082335	-2.8095897
N	11.3600446	12.8914562	-1.4443377
C	12.5517491	12.0218102	-3.4297684
H	15.7708169	8.7394466	-2.6822559
H	14.4957621	10.2208695	-4.5964917
H	15.7708169	8.7394466	2.6822559
H	14.4957621	10.2208695	4.5964917
H	10.5212037	13.7244801	4.5962683
H	8.8920951	14.8050926	2.6819928
H	8.8920951	14.8050926	-2.6819928
H	10.5212037	13.7244801	-4.5962683
Zn	12.4636134	-11.9220136	0.0000000
C	14.3714229	-10.2672360	-3.5080509
C	15.0132942	-9.5189177	-2.5469242
C	14.5017342	-9.9635208	-1.2651974
C	13.4557166	-11.1473707	-2.8099758
N	13.5650456	-10.9502156	-1.4443573
C	12.5517491	-12.0218102	-3.4297684
C	11.5697030	-12.8082335	-2.8095897

C	10.5823243	-13.6066541	-3.5078284
C	9.7596598	-14.1503272	-2.5466670
C	10.2640236	-13.6980268	-1.2650671
N	11.3600446	-12.8914562	-1.4443377
C	9.6967282	-14.0475890	0.0000000
C	10.2640236	-13.6980268	1.2650671
C	9.7596598	-14.1503272	2.5466670
C	10.5823243	-13.6066541	3.5078284
C	11.5697030	-12.8082335	2.8095897
N	11.3600446	-12.8914562	1.4443377
C	12.5517491	-12.0218102	3.4297684
C	13.4557166	-11.1473707	2.8099758
C	14.3714229	-10.2672360	3.5080509
C	15.0132942	-9.5189177	2.5469242
C	14.5017342	-9.9635208	1.2651974
N	13.5650456	-10.9502156	1.4443573
C	14.9178905	-9.4434985	0.0000000
H	14.4957621	-10.2208695	-4.5964917
H	15.7708169	-8.7394466	-2.6822559
H	10.5212037	-13.7244801	-4.5962683
H	8.8920951	-14.8050926	-2.6819928
H	8.8920951	-14.8050926	2.6819928
H	10.5212037	-13.7244801	4.5962683
H	14.4957621	-10.2208695	4.5964917
H	15.7708169	-8.7394466	2.6822559
Zn	-12.4636134	-11.9220136	0.0000000
C	-9.7596598	-14.1503272	2.5466670
C	-10.5823243	-13.6066541	3.5078284
C	-11.5697030	-12.8082335	2.8095897
C	-10.2640236	-13.6980268	1.2650671
N	-11.3600446	-12.8914562	1.4443377
C	-9.6967282	-14.0475890	0.0000000
C	-10.2640236	-13.6980268	-1.2650671
C	-9.7596598	-14.1503272	-2.5466670
C	-10.5823243	-13.6066541	-3.5078284
C	-11.5697030	-12.8082335	-2.8095897
N	-11.3600446	-12.8914562	-1.4443377
C	-12.5517491	-12.0218102	-3.4297684
C	-13.4557166	-11.1473707	-2.8099758
C	-14.3714229	-10.2672360	-3.5080509
C	-15.0132942	-9.5189177	-2.5469242
C	-14.5017342	-9.9635208	-1.2651974
N	-13.5650456	-10.9502156	-1.4443573
C	-14.9178905	-9.4434985	0.0000000
C	-14.5017342	-9.9635208	1.2651974
C	-15.0132942	-9.5189177	2.5469242
C	-14.3714229	-10.2672360	3.5080509
C	-13.4557166	-11.1473707	2.8099758
N	-13.5650456	-10.9502156	1.4443573

C	-12.5517491	-12.0218102	3.4297684
H	-8.8920951	-14.8050926	2.6819928
H	-10.5212037	-13.7244801	4.5962683
H	-8.8920951	-14.8050926	-2.6819928
H	-10.5212037	-13.7244801	-4.5962683
H	-14.4957621	-10.2208695	-4.5964917
H	-15.7708169	-8.7394466	-2.6822559
H	-15.7708169	-8.7394466	2.6822559
H	-14.4957621	-10.2208695	4.5964917
Zn	-12.4636134	11.9220136	0.0000000
C	-10.5823243	13.6066541	3.5078284
C	-9.7596598	14.1503272	2.5466670
C	-10.2640236	13.6980268	1.2650671
C	-11.5697030	12.8082335	2.8095897
N	-11.3600446	12.8914562	1.4443377
C	-12.5517491	12.0218102	3.4297684
C	-13.4557166	11.1473707	2.8099758
C	-14.3714229	10.2672360	3.5080509
C	-15.0132942	9.5189177	2.5469242
C	-14.5017342	9.9635208	1.2651974
N	-13.5650456	10.9502156	1.4443573
C	-14.9178905	9.4434985	0.0000000
C	-14.5017342	9.9635208	-1.2651974
C	-15.0132942	9.5189177	-2.5469242
C	-14.3714229	10.2672360	-3.5080509
C	-13.4557166	11.1473707	-2.8099758
N	-13.5650456	10.9502156	-1.4443573
C	-12.5517491	12.0218102	-3.4297684
C	-11.5697030	12.8082335	-2.8095897
C	-10.5823243	13.6066541	-3.5078284
C	-9.7596598	14.1503272	-2.5466670
C	-10.2640236	13.6980268	-1.2650671
N	-11.3600446	12.8914562	-1.4443377
C	-9.6967282	14.0475890	0.0000000
H	-10.5212037	13.7244801	4.5962683
H	-8.8920951	14.8050926	2.6819928
H	-14.4957621	10.2208695	4.5964917
H	-15.7708169	8.7394466	2.6822559
H	-15.7708169	8.7394466	-2.6822559
H	-14.4957621	10.2208695	-4.5964917
H	-10.5212037	13.7244801	-4.5962683
H	-8.8920951	14.8050926	-2.6819928
Zn	15.9578477	0.0000000	13.3259844
C	17.0120134	-3.5088676	11.0309823
C	17.3065630	-2.5470937	10.0909658
C	17.0220826	-1.2652122	10.7060156
C	16.5317883	-2.8108349	12.2067307
N	16.5577811	-1.4448390	11.9849278
C	16.0640011	-3.4310862	13.3737832

C	15.5029072	-2.8105186	14.4993883
C	14.9407128	-3.5086247	15.6379578
C	14.4316197	-2.5467334	16.4814544
C	14.7043013	-1.2650971	15.8613334
N	15.3553096	-1.4447637	14.6663563
C	14.3432590	0.0000000	16.4208659
C	14.7043013	1.2650971	15.8613334
C	14.4316197	2.5467334	16.4814544
C	14.9407128	3.5086247	15.6379578
C	15.5029072	2.8105186	14.4993883
N	15.3553096	1.4447637	14.6663563
C	16.0640011	3.4310862	13.3737832
C	16.5317883	2.8108349	12.2067307
C	17.0120134	3.5088676	11.0309823
C	17.3065630	2.5470937	10.0909658
C	17.0220826	1.2652122	10.7060156
N	16.5577811	1.4448390	11.9849278
C	17.2051454	0.0000000	10.0660780
H	17.1018788	-4.5975768	10.9363647
H	17.6886809	-2.6819689	9.0733904
H	14.9288877	-4.5973781	15.7675599
H	13.9219207	-2.6814740	17.4415149
H	13.9219207	2.6814740	17.4415149
H	14.9288877	4.5973781	15.7675599
H	17.1018788	4.5975768	10.9363647
H	17.6886809	2.6819689	9.0733904
Zn	15.9578477	0.0000000	-13.3259844
C	14.9407128	-3.5086247	-15.6379578
C	14.4316197	-2.5467334	-16.4814544
C	14.7043013	-1.2650971	-15.8613334
C	15.5029072	-2.8105186	-14.4993883
N	15.3553096	-1.4447637	-14.6663563
C	16.0640011	-3.4310862	-13.3737832
C	16.5317883	-2.8108349	-12.2067307
C	17.0120134	-3.5088676	-11.0309823
C	17.3065630	-2.5470937	-10.0909658
C	17.0220826	-1.2652122	-10.7060156
N	16.5577811	-1.4448390	-11.9849278
C	17.2051454	0.0000000	-10.0660780
C	17.0220826	1.2652122	-10.7060156
C	17.3065630	2.5470937	-10.0909658
C	17.0120134	3.5088676	-11.0309823
C	16.5317883	2.8108349	-12.2067307
N	16.5577811	1.4448390	-11.9849278
C	16.0640011	3.4310862	-13.3737832
C	15.5029072	2.8105186	-14.4993883
C	14.9407128	3.5086247	-15.6379578
C	14.4316197	2.5467334	-16.4814544
C	14.7043013	1.2650971	-15.8613334

N	15.3553096	1.4447637	-14.6663563
C	14.3432590	0.0000000	-16.4208659
H	14.9288877	-4.5973781	-15.7675599
H	13.9219207	-2.6814740	-17.4415149
H	17.1018788	-4.5975768	-10.9363647
H	17.6886809	-2.6819689	-9.0733904
H	17.6886809	2.6819689	-9.0733904
H	17.1018788	4.5975768	-10.9363647
H	14.9288877	4.5973781	-15.7675599
H	13.9219207	2.6814740	-17.4415149
Zn	-15.9578477	0.0000000	-13.3259844
C	-17.0120134	-3.5088676	-11.0309823
C	-17.3065630	-2.5470937	-10.0909658
C	-17.0220826	-1.2652122	-10.7060156
C	-16.5317883	-2.8108349	-12.2067307
N	-16.5577811	-1.4448390	-11.9849278
C	-16.0640011	-3.4310862	-13.3737832
C	-15.5029072	-2.8105186	-14.4993883
C	-14.9407128	-3.5086247	-15.6379578
C	-14.4316197	-2.5467334	-16.4814544
C	-14.7043013	-1.2650971	-15.8613334
N	-15.3553096	-1.4447637	-14.6663563
C	-14.3432590	0.0000000	-16.4208659
C	-14.7043013	1.2650971	-15.8613334
C	-14.4316197	2.5467334	-16.4814544
C	-14.9407128	3.5086247	-15.6379578
C	-15.5029072	2.8105186	-14.4993883
N	-15.3553096	1.4447637	-14.6663563
C	-16.0640011	3.4310862	-13.3737832
C	-16.5317883	2.8108349	-12.2067307
C	-17.0120134	3.5088676	-11.0309823
C	-17.3065630	2.5470937	-10.0909658
C	-17.0220826	1.2652122	-10.7060156
N	-16.5577811	1.4448390	-11.9849278
C	-17.2051454	0.0000000	-10.0660780
H	-17.1018788	-4.5975768	-10.9363647
H	-17.6886809	-2.6819689	-9.0733904
H	-14.9288877	-4.5973781	-15.7675599
H	-13.9219207	-2.6814740	-17.4415149
H	-13.9219207	2.6814740	-17.4415149
H	-14.9288877	4.5973781	-15.7675599
H	-17.1018788	4.5975768	-10.9363647
H	-17.6886809	2.6819689	-9.0733904
Zn	-15.9578477	0.0000000	13.3259844
C	-14.9407128	-3.5086247	15.6379578
C	-14.4316197	-2.5467334	16.4814544
C	-14.7043013	-1.2650971	15.8613334
C	-15.5029072	-2.8105186	14.4993883
N	-15.3553096	-1.4447637	14.6663563

C	-16.0640011	-3.4310862	13.3737832
C	-16.5317883	-2.8108349	12.2067307
C	-17.0120134	-3.5088676	11.0309823
C	-17.3065630	-2.5470937	10.0909658
C	-17.0220826	-1.2652122	10.7060156
N	-16.5577811	-1.4448390	11.9849278
C	-17.2051454	0.0000000	10.0660780
C	-17.0220826	1.2652122	10.7060156
C	-17.3065630	2.5470937	10.0909658
C	-17.0120134	3.5088676	11.0309823
C	-16.5317883	2.8108349	12.2067307
N	-16.5577811	1.4448390	11.9849278
C	-16.0640011	3.4310862	13.3737832
C	-15.5029072	2.8105186	14.4993883
C	-14.9407128	3.5086247	15.6379578
C	-14.4316197	2.5467334	16.4814544
C	-14.7043013	1.2650971	15.8613334
N	-15.3553096	1.4447637	14.6663563
C	-14.3432590	0.0000000	16.4208659
H	-14.9288877	-4.5973781	15.7675599
H	-13.9219207	-2.6814740	17.4415149
H	-17.1018788	-4.5975768	10.9363647
H	-17.6886809	-2.6819689	9.0733904
H	-17.6886809	2.6819689	9.0733904
H	-17.1018788	4.5975768	10.9363647
H	-14.9288877	4.5973781	15.7675599
H	-13.9219207	2.6814740	17.4415149
Zn	6.5976401	0.0000000	22.7236268
C	8.9279492	-3.5076859	21.7506354
C	9.7697266	-2.5466345	21.2368571
C	9.1438586	-1.2651378	21.4972358
C	7.7830320	-2.8097426	22.3003338
N	7.9466692	-1.4444116	22.1438460
C	6.6518989	-3.4296864	22.8506900
C	5.4726642	-2.8096522	23.2886608
C	4.2841548	-3.5075815	23.7363141
C	3.3311857	-2.5464759	23.9898802
C	3.9520335	-1.2650817	23.7177725
N	5.2466718	-1.4443752	23.2992660
C	3.3027107	0.0000000	23.8668193
C	3.9520335	1.2650817	23.7177725
C	3.3311857	2.5464759	23.9898802
C	4.2841548	3.5075815	23.7363141
C	5.4726642	2.8096522	23.2886608
N	5.2466718	1.4443752	23.2992660
C	6.6518989	3.4296864	22.8506900
C	7.7830320	2.8097426	22.3003338
C	8.9279492	3.5076859	21.7506354
C	9.7697266	2.5466345	21.2368571

C	9.1438586	1.2651378	21.4972358
N	7.9466692	1.4444116	22.1438460
C	9.7006257	0.0000000	21.1312641
H	9.0620525	-4.5959774	21.7482230
H	10.7326784	-2.6818540	20.7326261
H	4.1896212	-4.5958647	23.8313033
H	2.3012722	-2.6816072	24.3372588
H	2.3012722	2.6816072	24.3372588
H	4.1896212	4.5958647	23.8313033
H	9.0620525	4.5959774	21.7482230
H	10.7326784	2.6818540	20.7326261
Zn	6.5976401	0.0000000	-22.7236268
C	4.2841548	-3.5075815	-23.7363141
C	3.3311857	-2.5464759	-23.9898802
C	3.9520335	-1.2650817	-23.7177725
C	5.4726642	-2.8096522	-23.2886608
N	5.2466718	-1.4443752	-23.2992660
C	6.6518989	-3.4296864	-22.8506900
C	7.7830320	-2.8097426	-22.3003338
C	8.9279492	-3.5076859	-21.7506354
C	9.7697266	-2.5466345	-21.2368571
C	9.1438586	-1.2651378	-21.4972358
N	7.9466692	-1.4444116	-22.1438460
C	9.7006257	0.0000000	-21.1312641
C	9.1438586	1.2651378	-21.4972358
C	9.7697266	2.5466345	-21.2368571
C	8.9279492	3.5076859	-21.7506354
C	7.7830320	2.8097426	-22.3003338
N	7.9466692	1.4444116	-22.1438460
C	6.6518989	3.4296864	-22.8506900
C	5.4726642	2.8096522	-23.2886608
C	4.2841548	3.5075815	-23.7363141
C	3.3311857	2.5464759	-23.9898802
C	3.9520335	1.2650817	-23.7177725
N	5.2466718	1.4443752	-23.2992660
C	3.3027107	0.0000000	-23.8668193
H	4.1896212	-4.5958647	-23.8313033
H	2.3012722	-2.6816072	-24.3372588
H	9.0620525	-4.5959774	-21.7482230
H	10.7326784	-2.6818540	-20.7326261
H	10.7326784	2.6818540	-20.7326261
H	9.0620525	4.5959774	-21.7482230
H	4.1896212	4.5958647	-23.8313033
H	2.3012722	2.6816072	-24.3372588
Zn	-6.5976401	0.0000000	-22.7236268
C	-8.9279492	-3.5076859	-21.7506354
C	-9.7697266	-2.5466345	-21.2368571
C	-9.1438586	-1.2651378	-21.4972358
C	-7.7830320	-2.8097426	-22.3003338

N	-7.9466692	-1.4444116	-22.1438460
C	-6.6518989	-3.4296864	-22.8506900
C	-5.4726642	-2.8096522	-23.2886608
C	-4.2841548	-3.5075815	-23.7363141
C	-3.3311857	-2.5464759	-23.9898802
C	-3.9520335	-1.2650817	-23.7177725
N	-5.2466718	-1.4443752	-23.2992660
C	-3.3027107	0.0000000	-23.8668193
C	-3.9520335	1.2650817	-23.7177725
C	-3.3311857	2.5464759	-23.9898802
C	-4.2841548	3.5075815	-23.7363141
C	-5.4726642	2.8096522	-23.2886608
N	-5.2466718	1.4443752	-23.2992660
C	-6.6518989	3.4296864	-22.8506900
C	-7.7830320	2.8097426	-22.3003338
C	-8.9279492	3.5076859	-21.7506354
C	-9.7697266	2.5466345	-21.2368571
C	-9.1438586	1.2651378	-21.4972358
N	-7.9466692	1.4444116	-22.1438460
C	-9.7006257	0.0000000	-21.1312641
H	-9.0620525	-4.5959774	-21.7482230
H	-10.7326784	-2.6818540	-20.7326261
H	-4.1896212	-4.5958647	-23.8313033
H	-2.3012722	-2.6816072	-24.3372588
H	-2.3012722	2.6816072	-24.3372588
H	-4.1896212	4.5958647	-23.8313033
H	-9.0620525	4.5959774	-21.7482230
H	-10.7326784	2.6818540	-20.7326261
Zn	-6.5976401	0.0000000	22.7236268
C	-4.2841548	-3.5075815	23.7363141
C	-3.3311857	-2.5464759	23.9898802
C	-3.9520335	-1.2650817	23.7177725
C	-5.4726642	-2.8096522	23.2886608
N	-5.2466718	-1.4443752	23.2992660
C	-6.6518989	-3.4296864	22.8506900
C	-7.7830320	-2.8097426	22.3003338
C	-8.9279492	-3.5076859	21.7506354
C	-9.7697266	-2.5466345	21.2368571
C	-9.1438586	-1.2651378	21.4972358
N	-7.9466692	-1.4444116	22.1438460
C	-9.7006257	0.0000000	21.1312641
C	-9.1438586	1.2651378	21.4972358
C	-9.7697266	2.5466345	21.2368571
C	-8.9279492	3.5076859	21.7506354
C	-7.7830320	2.8097426	22.3003338
N	-7.9466692	1.4444116	22.1438460
C	-6.6518989	3.4296864	22.8506900
C	-5.4726642	2.8096522	23.2886608
C	-4.2841548	3.5075815	23.7363141

C	-3.3311857	2.5464759	23.9898802
C	-3.9520335	1.2650817	23.7177725
N	-5.2466718	1.4443752	23.2992660
C	-3.3027107	0.0000000	23.8668193
H	-4.1896212	-4.5958647	23.8313033
H	-2.3012722	-2.6816072	24.3372588
H	-9.0620525	-4.5959774	21.7482230
H	-10.7326784	-2.6818540	20.7326261
H	-10.7326784	2.6818540	20.7326261
H	-9.0620525	4.5959774	21.7482230
H	-4.1896212	4.5958647	23.8313033
H	-2.3012722	2.6816072	24.3372588
C	17.3802426	-4.8376786	0.0000000
C	16.9495189	-6.0051254	0.0000000
C	16.3761836	-7.2288507	0.0000000
C	15.7497134	-8.3043081	0.0000000
C	18.0272655	0.0000000	4.8929925
C	17.9405001	0.0000000	6.1337596
C	17.7759963	0.0000000	7.4751141
C	17.5552626	0.0000000	8.6996279
C	17.3802426	4.8376786	0.0000000
C	16.9495189	6.0051254	0.0000000
C	16.3761836	7.2288507	0.0000000
C	15.7497134	8.3043081	0.0000000
C	18.0272655	0.0000000	-4.8929925
C	17.9405001	0.0000000	-6.1337596
C	17.7759963	0.0000000	-7.4751141
C	17.5552626	0.0000000	-8.6996279
C	-17.3802426	-4.8376786	0.0000000
C	-16.9495189	-6.0051254	0.0000000
C	-16.3761836	-7.2288507	0.0000000
C	-15.7497134	-8.3043081	0.0000000
C	-18.0272655	0.0000000	-4.8929925
C	-17.9405001	0.0000000	-6.1337596
C	-17.7759963	0.0000000	-7.4751141
C	-17.5552626	0.0000000	-8.6996279
C	-17.3802426	4.8376786	0.0000000
C	-16.9495189	6.0051254	0.0000000
C	-16.3761836	7.2288507	0.0000000
C	-15.7497134	8.3043081	0.0000000
C	-18.0272655	0.0000000	4.8929925
C	-17.9405001	0.0000000	6.1337596
C	-17.7759963	0.0000000	7.4751141
C	-17.5552626	0.0000000	8.6996279
C	-4.8691765	16.0615946	0.0000000
C	-6.0654426	15.7183155	0.0000000
C	-7.3344466	15.2536256	0.0000000
C	-8.4695636	14.7432813	0.0000000
C	4.8691765	16.0615946	0.0000000

C	6.0654426	15.7183155	0.0000000
C	7.3344466	15.2536256	0.0000000
C	8.4695636	14.7432813	0.0000000
C	-4.8691765	-16.0615946	0.0000000
C	-6.0654426	-15.7183155	0.0000000
C	-7.3344466	-15.2536256	0.0000000
C	-8.4695636	-14.7432813	0.0000000
C	4.8691765	-16.0615946	0.0000000
C	6.0654426	-15.7183155	0.0000000
C	7.3344466	-15.2536256	0.0000000
C	8.4695636	-14.7432813	0.0000000
C	13.5347708	0.0000000	17.5767211
C	12.7315046	0.0000000	18.5272517
C	11.7848985	0.0000000	19.4915798
C	10.8481272	0.0000000	20.3110246
C	13.5347708	0.0000000	-17.5767211
C	12.7315046	0.0000000	-18.5272517
C	11.7848985	0.0000000	-19.4915798
C	10.8481272	0.0000000	-20.3110246
C	-13.5347708	0.0000000	-17.5767211
C	-12.7315046	0.0000000	-18.5272517
C	-11.7848985	0.0000000	-19.4915798
C	-10.8481272	0.0000000	-20.3110246
C	-13.5347708	0.0000000	17.5767211
C	-12.7315046	0.0000000	18.5272517
C	-11.7848985	0.0000000	19.4915798
C	-10.8481272	0.0000000	20.3110246
C	1.9161031	0.0000000	24.1248644
C	0.6756228	0.0000000	24.2258685
C	-0.6756228	0.0000000	24.2258685
C	-1.9161031	0.0000000	24.1248644
C	1.9161031	0.0000000	-24.1248644
C	0.6756228	0.0000000	-24.2258685
C	-0.6756228	0.0000000	-24.2258685
C	-1.9161031	0.0000000	-24.1248644
H	0.0000000	16.7100262	4.5294492
H	0.0000000	16.7100262	-4.5294492
H	0.0000000	-16.7100262	-4.5294492
H	0.0000000	-16.7100262	4.5294492
H	12.5851394	12.0599810	4.5286581
H	12.5851394	12.0599810	-4.5286581
H	12.5851394	-12.0599810	-4.5286581
H	12.5851394	-12.0599810	4.5286581
H	-12.5851394	-12.0599810	-4.5286581
H	-12.5851394	-12.0599810	4.5286581
H	-12.5851394	12.0599810	4.5286581
H	-12.5851394	12.0599810	-4.5286581
H	16.1051327	-4.5302065	13.3923376
H	16.1051327	4.5302065	13.3923376

```
H    16.1051327   -4.5302065  -13.3923376
H    16.1051327    4.5302065  -13.3923376
H   -16.1051327   -4.5302065  -13.3923376
H   -16.1051327    4.5302065  -13.3923376
H   -16.1051327   -4.5302065   13.3923376
H   -16.1051327    4.5302065   13.3923376
H     6.6728399   -4.5284502   22.8996957
H     6.6728399    4.5284502   22.8996957
H     6.6728399   -4.5284502  -22.8996957
H     6.6728399    4.5284502  -22.8996957
H    -6.6728399   -4.5284502  -22.8996957
H    -6.6728399    4.5284502  -22.8996957
H    -6.6728399   -4.5284502   22.8996957
H    -6.6728399    4.5284502   22.8996957
```

Molecule 6

```
706
Energy =
Zn   -0.0000000    0.0000000   20.8247938
C     2.5386225   -3.5122541   20.7511749
C     3.5122541   -2.5386225   20.7511749
C     2.8254408   -1.2668010   20.7852320
C     1.2668010   -2.8254408   20.7852320
N     1.4610322   -1.4610322   20.8176035
C     0.0000000   -3.4815184   20.7477999
C    -1.2668010   -2.8254408   20.7852320
C    -2.5386225   -3.5122541   20.7511749
C    -3.5122541   -2.5386225   20.7511749
C    -2.8254408   -1.2668010   20.7852320
N    -1.4610322   -1.4610322   20.8176035
C    -3.4815184    0.0000000   20.7477999
C    -2.8254408    1.2668010   20.7852320
C    -3.5122541    2.5386225   20.7511749
C    -2.5386225    3.5122541   20.7511749
C    -1.2668010    2.8254408   20.7852320
N    -1.4610322    1.4610322   20.8176035
C    -0.0000000    3.4815184   20.7477999
C     1.2668010    2.8254408   20.7852320
C     2.5386225    3.5122541   20.7511749
C     3.5122541    2.5386225   20.7511749
C     2.8254408    1.2668010   20.7852320
N     1.4610322    1.4610322   20.8176035
C     3.4815184    0.0000000   20.7477999
H     2.6636871   -4.5998289   20.7268269
H     4.5998289   -2.6636871   20.7268269
H    -2.6636871   -4.5998289   20.7268269
H    -4.5998289   -2.6636871   20.7268269
H    -4.5998289    2.6636871   20.7268269
H    -2.6636871    4.5998289   20.7268269
```

H	2.6636871	4.5998289	20.7268269
H	4.5998289	2.6636871	20.7268269
Zn	-0.0000000	0.0000000	-20.8247938
C	2.5386225	3.5122541	-20.7511749
C	3.5122541	2.5386225	-20.7511749
C	2.8254408	1.2668010	-20.7852320
C	1.2668010	2.8254408	-20.7852320
N	1.4610322	1.4610322	-20.8176035
C	-0.0000000	3.4815184	-20.7477999
C	-1.2668010	2.8254408	-20.7852320
C	-2.5386225	3.5122541	-20.7511749
C	-3.5122541	2.5386225	-20.7511749
C	-2.8254408	1.2668010	-20.7852320
N	-1.4610322	1.4610322	-20.8176035
C	-3.4815184	0.0000000	-20.7477999
C	-2.8254408	-1.2668010	-20.7852320
C	-3.5122541	-2.5386225	-20.7511749
C	-2.5386225	-3.5122541	-20.7511749
C	-1.2668010	-2.8254408	-20.7852320
N	-1.4610322	-1.4610322	-20.8176035
C	0.0000000	-3.4815184	-20.7477999
C	1.2668010	-2.8254408	-20.7852320
C	2.5386225	-3.5122541	-20.7511749
C	3.5122541	-2.5386225	-20.7511749
C	2.8254408	-1.2668010	-20.7852320
N	1.4610322	-1.4610322	-20.8176035
C	3.4815184	0.0000000	-20.7477999
H	2.6636871	4.5998289	-20.7268269
H	4.5998289	2.6636871	-20.7268269
H	-2.6636871	4.5998289	-20.7268269
H	-4.5998289	2.6636871	-20.7268269
H	-4.5998289	-2.6636871	-20.7268269
H	-2.6636871	-4.5998289	-20.7268269
H	2.6636871	-4.5998289	-20.7268269
H	4.5998289	-2.6636871	-20.7268269
Zn	12.8923069	0.0000000	17.2642734
C	15.7531656	2.5468342	15.2389175
C	15.0044677	3.5086429	15.8794197
C	13.9738945	2.8104038	16.6211488
C	15.1863650	1.2650749	15.6096336
N	14.1142421	1.4446999	16.4478036
C	15.6802400	0.0000000	15.1628393
C	15.1863650	-1.2650749	15.6096336
C	15.7531656	-2.5468342	15.2389175
C	15.0044677	-3.5086429	15.8794197
C	13.9738945	-2.8104038	16.6211488
N	14.1142421	-1.4446999	16.4478036
C	12.9566337	-3.4309662	17.3607824
C	11.8832458	-2.8107660	18.0155354

C	10.8021992	-3.5088683	18.6820491
C	9.9234098	-2.5471307	19.1272204
C	10.4838007	-1.2651896	18.7464272
N	11.6692784	-1.4447436	18.0786863
C	9.8820838	-0.0000000	19.0310907
C	10.4838007	1.2651896	18.7464272
C	9.9234098	2.5471307	19.1272204
C	10.8021992	3.5088683	18.6820491
C	11.8832458	2.8107660	18.0155354
N	11.6692784	1.4447436	18.0786863
C	12.9566337	3.4309662	17.3607824
H	16.6165348	2.6817353	14.5785768
H	15.1301759	4.5974141	15.8463471
H	16.6165348	-2.6817353	14.5785768
H	15.1301759	-4.5974141	15.8463471
H	10.7231620	-4.5976301	18.7853229
H	8.9820314	-2.6820879	19.6705886
H	8.9820314	2.6820879	19.6705886
H	10.7231620	4.5976301	18.7853229
Zn	-12.8923069	0.0000000	17.2642734
C	-10.8021992	-3.5088683	18.6820491
C	-9.9234098	-2.5471307	19.1272204
C	-10.4838007	-1.2651896	18.7464272
C	-11.8832458	-2.8107660	18.0155354
N	-11.6692784	-1.4447436	18.0786863
C	-12.9566337	-3.4309662	17.3607824
C	-13.9738945	-2.8104038	16.6211488
C	-15.0044677	-3.5086429	15.8794197
C	-15.7531656	-2.5468342	15.2389175
C	-15.1863650	-1.2650749	15.6096336
N	-14.1142421	-1.4446999	16.4478036
C	-15.6802400	0.0000000	15.1628393
C	-15.1863650	1.2650749	15.6096336
C	-15.7531656	2.5468342	15.2389175
C	-15.0044677	3.5086429	15.8794197
C	-13.9738945	2.8104038	16.6211488
N	-14.1142421	1.4446999	16.4478036
C	-12.9566337	3.4309662	17.3607824
C	-11.8832458	2.8107660	18.0155354
C	-10.8021992	3.5088683	18.6820491
C	-9.9234098	2.5471307	19.1272204
C	-10.4838007	1.2651896	18.7464272
N	-11.6692784	1.4447436	18.0786863
C	-9.8820838	0.0000000	19.0310907
H	-10.7231620	-4.5976301	18.7853229
H	-8.9820314	-2.6820879	19.6705886
H	-15.1301759	-4.5974141	15.8463471
H	-16.6165348	-2.6817353	14.5785768
H	-16.6165348	2.6817353	14.5785768

H	-15.1301759	4.5974141	15.8463471
H	-10.7231620	4.5976301	18.7853229
H	-8.9820314	2.6820879	19.6705886
Zn	-12.8923069	0.0000000	-17.2642734
C	-10.8021992	3.5088683	-18.6820491
C	-9.9234098	2.5471307	-19.1272204
C	-10.4838007	1.2651896	-18.7464272
C	-11.8832458	2.8107660	-18.0155354
N	-11.6692784	1.4447436	-18.0786863
C	-12.9566337	3.4309662	-17.3607824
C	-13.9738945	2.8104038	-16.6211488
C	-15.0044677	3.5086429	-15.8794197
C	-15.7531656	2.5468342	-15.2389175
C	-15.1863650	1.2650749	-15.6096336
N	-14.1142421	1.4446999	-16.4478036
C	-15.6802400	0.0000000	-15.1628393
C	-15.1863650	-1.2650749	-15.6096336
C	-15.7531656	-2.5468342	-15.2389175
C	-15.0044677	-3.5086429	-15.8794197
C	-13.9738945	-2.8104038	-16.6211488
N	-14.1142421	-1.4446999	-16.4478036
C	-12.9566337	-3.4309662	-17.3607824
C	-11.8832458	-2.8107660	-18.0155354
C	-10.8021992	-3.5088683	-18.6820491
C	-9.9234098	-2.5471307	-19.1272204
C	-10.4838007	-1.2651896	-18.7464272
N	-11.6692784	-1.4447436	-18.0786863
C	-9.8820838	0.0000000	-19.0310907
H	-10.7231620	4.5976301	-18.7853229
H	-8.9820314	2.6820879	-19.6705886
H	-15.1301759	4.5974141	-15.8463471
H	-16.6165348	2.6817353	-14.5785768
H	-16.6165348	-2.6817353	-14.5785768
H	-15.1301759	-4.5974141	-15.8463471
H	-10.7231620	-4.5976301	-18.7853229
H	-8.9820314	-2.6820879	-19.6705886
Zn	12.8923069	0.0000000	-17.2642734
C	15.7531656	-2.5468342	-15.2389175
C	15.0044677	-3.5086429	-15.8794197
C	13.9738945	-2.8104038	-16.6211488
C	15.1863650	-1.2650749	-15.6096336
N	14.1142421	-1.4446999	-16.4478036
C	15.6802400	0.0000000	-15.1628393
C	15.1863650	1.2650749	-15.6096336
C	15.7531656	2.5468342	-15.2389175
C	15.0044677	3.5086429	-15.8794197
C	13.9738945	2.8104038	-16.6211488
N	14.1142421	1.4446999	-16.4478036
C	12.9566337	3.4309662	-17.3607824

C	11.8832458	2.8107660	-18.0155354
C	10.8021992	3.5088683	-18.6820491
C	9.9234098	2.5471307	-19.1272204
C	10.4838007	1.2651896	-18.7464272
N	11.6692784	1.4447436	-18.0786863
C	9.8820838	-0.0000000	-19.0310907
C	10.4838007	-1.2651896	-18.7464272
C	9.9234098	-2.5471307	-19.1272204
C	10.8021992	-3.5088683	-18.6820491
C	11.8832458	-2.8107660	-18.0155354
N	11.6692784	-1.4447436	-18.0786863
C	12.9566337	-3.4309662	-17.3607824
H	16.6165348	-2.6817353	-14.5785768
H	15.1301759	-4.5974141	-15.8463471
H	16.6165348	2.6817353	-14.5785768
H	15.1301759	4.5974141	-15.8463471
H	10.7231620	4.5976301	-18.7853229
H	8.9820314	2.6820879	-19.6705886
H	8.9820314	-2.6820879	-19.6705886
H	10.7231620	-4.5976301	-18.7853229
Zn	21.0335620	-0.0000000	6.6757291
C	21.8685961	-3.5086718	4.2921375
C	22.0739051	-2.5468820	3.3285095
C	21.8494017	-1.2650417	3.9674574
C	21.5012378	-2.8104394	5.5076308
N	21.5075976	-1.4445935	5.2846572
C	21.1428780	-3.4308511	6.7130646
C	20.6885014	-2.8105056	7.8856194
C	20.2355325	-3.5087095	9.0719043
C	19.8086329	-2.5469383	9.9598405
C	20.0218633	-1.2650680	9.3170121
N	20.5570217	-1.4446155	8.0658981
C	19.7170696	0.0000000	9.9091393
C	20.0218633	1.2650680	9.3170121
C	19.8086329	2.5469383	9.9598405
C	20.2355325	3.5087095	9.0719043
C	20.6885014	2.8105056	7.8856194
N	20.5570217	1.4446155	8.0658981
C	21.1428780	3.4308511	6.7130646
C	21.5012378	2.8104394	5.5076308
C	21.8685961	3.5086718	4.2921375
C	22.0739051	2.5468820	3.3285095
C	21.8494017	1.2650417	3.9674574
N	21.5075976	1.4445935	5.2846572
C	21.9699843	0.0000000	3.3124250
H	21.9481188	-4.5974614	4.1894338
H	22.3580501	-2.6817671	2.2793476
H	20.2355975	-4.5974981	9.2017873
H	19.3917033	-2.6818312	10.9636422

H	19.3917033	2.6818312	10.9636422
H	20.2355975	4.5974981	9.2017873
H	21.9481188	4.5974614	4.1894338
H	22.3580501	2.6817671	2.2793476
Zn	-21.0335620	0.0000000	6.6757291
C	-21.8685961	3.5086718	4.2921375
C	-22.0739051	2.5468820	3.3285095
C	-21.8494017	1.2650417	3.9674574
C	-21.5012378	2.8104394	5.5076308
N	-21.5075976	1.4445935	5.2846572
C	-21.1428780	3.4308511	6.7130646
C	-20.6885014	2.8105056	7.8856194
C	-20.2355325	3.5087095	9.0719043
C	-19.8086329	2.5469383	9.9598405
C	-20.0218633	1.2650680	9.3170121
N	-20.5570217	1.4446155	8.0658981
C	-19.7170696	0.0000000	9.9091393
C	-20.0218633	-1.2650680	9.3170121
C	-19.8086329	-2.5469383	9.9598405
C	-20.2355325	-3.5087095	9.0719043
C	-20.6885014	-2.8105056	7.8856194
N	-20.5570217	-1.4446155	8.0658981
C	-21.1428780	-3.4308511	6.7130646
C	-21.5012378	-2.8104394	5.5076308
C	-21.8685961	-3.5086718	4.2921375
C	-22.0739051	-2.5468820	3.3285095
C	-21.8494017	-1.2650417	3.9674574
N	-21.5075976	-1.4445935	5.2846572
C	-21.9699843	-0.0000000	3.3124250
H	-21.9481188	4.5974614	4.1894338
H	-22.3580501	2.6817671	2.2793476
H	-20.2355975	4.5974981	9.2017873
H	-19.3917033	2.6818312	10.9636422
H	-19.3917033	-2.6818312	10.9636422
H	-20.2355975	-4.5974981	9.2017873
H	-21.9481188	-4.5974614	4.1894338
H	-22.3580501	-2.6817671	2.2793476
Zn	-21.0335620	0.0000000	-6.6757291
C	-22.0739051	2.5468820	-3.3285095
C	-21.8685961	3.5086718	-4.2921375
C	-21.5012378	2.8104394	-5.5076308
C	-21.8494017	1.2650417	-3.9674574
N	-21.5075976	1.4445935	-5.2846572
C	-21.9699843	-0.0000000	-3.3124250
C	-21.8494017	-1.2650417	-3.9674574
C	-22.0739051	-2.5468820	-3.3285095
C	-21.8685961	-3.5086718	-4.2921375
C	-21.5012378	-2.8104394	-5.5076308
N	-21.5075976	-1.4445935	-5.2846572

C	-21.1428780	-3.4308511	-6.7130646
C	-20.6885014	-2.8105056	-7.8856194
C	-20.2355325	-3.5087095	-9.0719043
C	-19.8086329	-2.5469383	-9.9598405
C	-20.0218633	-1.2650680	-9.3170121
N	-20.5570217	-1.4446155	-8.0658981
C	-19.7170696	0.0000000	-9.9091393
C	-20.0218633	1.2650680	-9.3170121
C	-19.8086329	2.5469383	-9.9598405
C	-20.2355325	3.5087095	-9.0719043
C	-20.6885014	2.8105056	-7.8856194
N	-20.5570217	1.4446155	-8.0658981
C	-21.1428780	3.4308511	-6.7130646
H	-22.3580501	2.6817671	-2.2793476
H	-21.9481188	4.5974614	-4.1894338
H	-22.3580501	-2.6817671	-2.2793476
H	-21.9481188	-4.5974614	-4.1894338
H	-20.2355975	-4.5974981	-9.2017873
H	-19.3917033	-2.6818312	-10.9636422
H	-19.3917033	2.6818312	-10.9636422
H	-20.2355975	4.5974981	-9.2017873
Zn	21.0335620	-0.0000000	-6.6757291
C	22.0739051	-2.5468820	-3.3285095
C	21.8685961	-3.5086718	-4.2921375
C	21.5012378	-2.8104394	-5.5076308
C	21.8494017	-1.2650417	-3.9674574
N	21.5075976	-1.4445935	-5.2846572
C	21.9699843	-0.0000000	-3.3124250
C	21.8494017	1.2650417	-3.9674574
C	22.0739051	2.5468820	-3.3285095
C	21.8685961	3.5086718	-4.2921375
C	21.5012378	2.8104394	-5.5076308
N	21.5075976	1.4445935	-5.2846572
C	21.1428780	3.4308511	-6.7130646
C	20.6885014	2.8105056	-7.8856194
C	20.2355325	3.5087095	-9.0719043
C	19.8086329	2.5469383	-9.9598405
C	20.0218633	1.2650680	-9.3170121
N	20.5570217	1.4446155	-8.0658981
C	19.7170696	0.0000000	-9.9091393
C	20.0218633	-1.2650680	-9.3170121
C	19.8086329	-2.5469383	-9.9598405
C	20.2355325	-3.5087095	-9.0719043
C	20.6885014	-2.8105056	-7.8856194
N	20.5570217	-1.4446155	-8.0658981
C	21.1428780	-3.4308511	-6.7130646
H	22.3580501	-2.6817671	-2.2793476
H	21.9481188	-4.5974614	-4.1894338
H	22.3580501	2.6817671	-2.2793476

H	21.9481188	4.5974614	-4.1894338
H	20.2355975	4.5974981	-9.2017873
H	19.3917033	2.6818312	-10.9636422
H	19.3917033	-2.6818312	-10.9636422
H	20.2355975	-4.5974981	-9.2017873
Zn	0.0000000	12.8923069	17.2642734
C	2.5471307	9.9234098	19.1272204
C	3.5088683	10.8021992	18.6820491
C	2.8107660	11.8832458	18.0155354
C	1.2651896	10.4838007	18.7464272
N	1.4447436	11.6692784	18.0786863
C	0.0000000	9.8820838	19.0310907
C	-1.2651896	10.4838007	18.7464272
C	-2.5471307	9.9234098	19.1272204
C	-3.5088683	10.8021992	18.6820491
C	-2.8107660	11.8832458	18.0155354
N	-1.4447436	11.6692784	18.0786863
C	-3.4309662	12.9566337	17.3607824
C	-2.8104038	13.9738945	16.6211488
C	-3.5086429	15.0044677	15.8794197
C	-2.5468342	15.7531656	15.2389175
C	-1.2650749	15.1863650	15.6096336
N	-1.4446999	14.1142421	16.4478036
C	0.0000000	15.6802400	15.1628393
C	1.2650749	15.1863650	15.6096336
C	2.5468342	15.7531656	15.2389175
C	3.5086429	15.0044677	15.8794197
C	2.8104038	13.9738945	16.6211488
N	1.4446999	14.1142421	16.4478036
C	3.4309662	12.9566337	17.3607824
H	2.6820879	8.9820314	19.6705886
H	4.5976301	10.7231620	18.7853229
H	-2.6820879	8.9820314	19.6705886
H	-4.5976301	10.7231620	18.7853229
H	-4.5974141	15.1301759	15.8463471
H	-2.6817353	16.6165348	14.5785768
H	2.6817353	16.6165348	14.5785768
H	4.5974141	15.1301759	15.8463471
Zn	-0.0000000	-12.8923069	17.2642734
C	3.5088683	-10.8021992	18.6820491
C	2.5471307	-9.9234098	19.1272204
C	1.2651896	-10.4838007	18.7464272
C	2.8107660	-11.8832458	18.0155354
N	1.4447436	-11.6692784	18.0786863
C	3.4309662	-12.9566337	17.3607824
C	2.8104038	-13.9738945	16.6211488
C	3.5086429	-15.0044677	15.8794197
C	2.5468342	-15.7531656	15.2389175
C	1.2650749	-15.1863650	15.6096336

N	1.4446999	-14.1142421	16.4478036
C	0.0000000	-15.6802400	15.1628393
C	-1.2650749	-15.1863650	15.6096336
C	-2.5468342	-15.7531656	15.2389175
C	-3.5086429	-15.0044677	15.8794197
C	-2.8104038	-13.9738945	16.6211488
N	-1.4446999	-14.1142421	16.4478036
C	-3.4309662	-12.9566337	17.3607824
C	-2.8107660	-11.8832458	18.0155354
C	-3.5088683	-10.8021992	18.6820491
C	-2.5471307	-9.9234098	19.1272204
C	-1.2651896	-10.4838007	18.7464272
N	-1.4447436	-11.6692784	18.0786863
C	-0.0000000	-9.8820838	19.0310907
H	4.5976301	-10.7231620	18.7853229
H	2.6820879	-8.9820314	19.6705886
H	4.5974141	-15.1301759	15.8463471
H	2.6817353	-16.6165348	14.5785768
H	-2.6817353	-16.6165348	14.5785768
H	-4.5974141	-15.1301759	15.8463471
H	-4.5976301	-10.7231620	18.7853229
H	-2.6820879	-8.9820314	19.6705886
Zn	-0.0000000	-12.8923069	-17.2642734
C	2.5471307	-9.9234098	-19.1272204
C	3.5088683	-10.8021992	-18.6820491
C	2.8107660	-11.8832458	-18.0155354
C	1.2651896	-10.4838007	-18.7464272
N	1.4447436	-11.6692784	-18.0786863
C	-0.0000000	-9.8820838	-19.0310907
C	-1.2651896	-10.4838007	-18.7464272
C	-2.5471307	-9.9234098	-19.1272204
C	-3.5088683	-10.8021992	-18.6820491
C	-2.8107660	-11.8832458	-18.0155354
N	-1.4447436	-11.6692784	-18.0786863
C	-3.4309662	-12.9566337	-17.3607824
C	-2.8104038	-13.9738945	-16.6211488
C	-3.5086429	-15.0044677	-15.8794197
C	-2.5468342	-15.7531656	-15.2389175
C	-1.2650749	-15.1863650	-15.6096336
N	-1.4446999	-14.1142421	-16.4478036
C	0.0000000	-15.6802400	-15.1628393
C	1.2650749	-15.1863650	-15.6096336
C	2.5468342	-15.7531656	-15.2389175
C	3.5086429	-15.0044677	-15.8794197
C	2.8104038	-13.9738945	-16.6211488
N	1.4446999	-14.1142421	-16.4478036
C	3.4309662	-12.9566337	-17.3607824
H	2.6820879	-8.9820314	-19.6705886
H	4.5976301	-10.7231620	-18.7853229

H	-2.6820879	-8.9820314	-19.6705886
H	-4.5976301	-10.7231620	-18.7853229
H	-4.5974141	-15.1301759	-15.8463471
H	-2.6817353	-16.6165348	-14.5785768
H	2.6817353	-16.6165348	-14.5785768
H	4.5974141	-15.1301759	-15.8463471
Zn	0.0000000	12.8923069	-17.2642734
C	3.5088683	10.8021992	-18.6820491
C	2.5471307	9.9234098	-19.1272204
C	1.2651896	10.4838007	-18.7464272
C	2.8107660	11.8832458	-18.0155354
N	1.4447436	11.6692784	-18.0786863
C	3.4309662	12.9566337	-17.3607824
C	2.8104038	13.9738945	-16.6211488
C	3.5086429	15.0044677	-15.8794197
C	2.5468342	15.7531656	-15.2389175
C	1.2650749	15.1863650	-15.6096336
N	1.4446999	14.1142421	-16.4478036
C	0.0000000	15.6802400	-15.1628393
C	-1.2650749	15.1863650	-15.6096336
C	-2.5468342	15.7531656	-15.2389175
C	-3.5086429	15.0044677	-15.8794197
C	-2.8104038	13.9738945	-16.6211488
N	-1.4446999	14.1142421	-16.4478036
C	-3.4309662	12.9566337	-17.3607824
C	-2.8107660	11.8832458	-18.0155354
C	-3.5088683	10.8021992	-18.6820491
C	-2.5471307	9.9234098	-19.1272204
C	-1.2651896	10.4838007	-18.7464272
N	-1.4447436	11.6692784	-18.0786863
C	0.0000000	9.8820838	-19.0310907
H	4.5976301	10.7231620	-18.7853229
H	2.6820879	8.9820314	-19.6705886
H	4.5974141	15.1301759	-15.8463471
H	2.6817353	16.6165348	-14.5785768
H	-2.6817353	16.6165348	-14.5785768
H	-4.5974141	15.1301759	-15.8463471
H	-4.5976301	10.7231620	-18.7853229
H	-2.6820879	8.9820314	-19.6705886
Zn	0.0000000	21.0335620	6.6757291
C	3.5086718	21.8685961	4.2921375
C	2.5468820	22.0739051	3.3285095
C	1.2650417	21.8494017	3.9674574
C	2.8104394	21.5012378	5.5076308
N	1.4445935	21.5075976	5.2846572
C	3.4308511	21.1428780	6.7130646
C	2.8105056	20.6885014	7.8856194
C	3.5087095	20.2355325	9.0719043
C	2.5469383	19.8086329	9.9598405

C	1.2650680	20.0218633	9.3170121
N	1.4446155	20.5570217	8.0658981
C	0.0000000	19.7170696	9.9091393
C	-1.2650680	20.0218633	9.3170121
C	-2.5469383	19.8086329	9.9598405
C	-3.5087095	20.2355325	9.0719043
C	-2.8105056	20.6885014	7.8856194
N	-1.4446155	20.5570217	8.0658981
C	-3.4308511	21.1428780	6.7130646
C	-2.8104394	21.5012378	5.5076308
C	-3.5086718	21.8685961	4.2921375
C	-2.5468820	22.0739051	3.3285095
C	-1.2650417	21.8494017	3.9674574
N	-1.4445935	21.5075976	5.2846572
C	-0.0000000	21.9699843	3.3124250
H	4.5974614	21.9481188	4.1894338
H	2.6817671	22.3580501	2.2793476
H	4.5974981	20.2355975	9.2017873
H	2.6818312	19.3917033	10.9636422
H	-2.6818312	19.3917033	10.9636422
H	-4.5974981	20.2355975	9.2017873
H	-4.5974614	21.9481188	4.1894338
H	-2.6817671	22.3580501	2.2793476
Zn	-0.0000000	-21.0335620	6.6757291
C	2.5468820	-22.0739051	3.3285095
C	3.5086718	-21.8685961	4.2921375
C	2.8104394	-21.5012378	5.5076308
C	1.2650417	-21.8494017	3.9674574
N	1.4445935	-21.5075976	5.2846572
C	0.0000000	-21.9699843	3.3124250
C	-1.2650417	-21.8494017	3.9674574
C	-2.5468820	-22.0739051	3.3285095
C	-3.5086718	-21.8685961	4.2921375
C	-2.8104394	-21.5012378	5.5076308
N	-1.4445935	-21.5075976	5.2846572
C	-3.4308511	-21.1428780	6.7130646
C	-2.8105056	-20.6885014	7.8856194
C	-3.5087095	-20.2355325	9.0719043
C	-2.5469383	-19.8086329	9.9598405
C	-1.2650680	-20.0218633	9.3170121
N	-1.4446155	-20.5570217	8.0658981
C	0.0000000	-19.7170696	9.9091393
C	1.2650680	-20.0218633	9.3170121
C	2.5469383	-19.8086329	9.9598405
C	3.5087095	-20.2355325	9.0719043
C	2.8105056	-20.6885014	7.8856194
N	1.4446155	-20.5570217	8.0658981
C	3.4308511	-21.1428780	6.7130646
H	2.6817671	-22.3580501	2.2793476

H	4.5974614	-21.9481188	4.1894338
H	-2.6817671	-22.3580501	2.2793476
H	-4.5974614	-21.9481188	4.1894338
H	-4.5974981	-20.2355975	9.2017873
H	-2.6818312	-19.3917033	10.9636422
H	2.6818312	-19.3917033	10.9636422
H	4.5974981	-20.2355975	9.2017873
Zn	-0.0000000	-21.0335620	-6.6757291
C	3.5086718	-21.8685961	-4.2921375
C	2.5468820	-22.0739051	-3.3285095
C	1.2650417	-21.8494017	-3.9674574
C	2.8104394	-21.5012378	-5.5076308
N	1.4445935	-21.5075976	-5.2846572
C	3.4308511	-21.1428780	-6.7130646
C	2.8105056	-20.6885014	-7.8856194
C	3.5087095	-20.2355325	-9.0719043
C	2.5469383	-19.8086329	-9.9598405
C	1.2650680	-20.0218633	-9.3170121
N	1.4446155	-20.5570217	-8.0658981
C	0.0000000	-19.7170696	-9.9091393
C	-1.2650680	-20.0218633	-9.3170121
C	-2.5469383	-19.8086329	-9.9598405
C	-3.5087095	-20.2355325	-9.0719043
C	-2.8105056	-20.6885014	-7.8856194
N	-1.4446155	-20.5570217	-8.0658981
C	-3.4308511	-21.1428780	-6.7130646
C	-2.8104394	-21.5012378	-5.5076308
C	-3.5086718	-21.8685961	-4.2921375
C	-2.5468820	-22.0739051	-3.3285095
C	-1.2650417	-21.8494017	-3.9674574
N	-1.4445935	-21.5075976	-5.2846572
C	-0.0000000	-21.9699843	-3.3124250
H	4.5974614	-21.9481188	-4.1894338
H	2.6817671	-22.3580501	-2.2793476
H	4.5974981	-20.2355975	-9.2017873
H	2.6818312	-19.3917033	-10.9636422
H	-2.6818312	-19.3917033	-10.9636422
H	-4.5974981	-20.2355975	-9.2017873
H	-4.5974614	-21.9481188	-4.1894338
H	-2.6817671	-22.3580501	-2.2793476
Zn	0.0000000	21.0335620	-6.6757291
C	2.5468820	22.0739051	-3.3285095
C	3.5086718	21.8685961	-4.2921375
C	2.8104394	21.5012378	-5.5076308
C	1.2650417	21.8494017	-3.9674574
N	1.4445935	21.5075976	-5.2846572
C	-0.0000000	21.9699843	-3.3124250
C	-1.2650417	21.8494017	-3.9674574
C	-2.5468820	22.0739051	-3.3285095

C	-3.5086718	21.8685961	-4.2921375
C	-2.8104394	21.5012378	-5.5076308
N	-1.4445935	21.5075976	-5.2846572
C	-3.4308511	21.1428780	-6.7130646
C	-2.8105056	20.6885014	-7.8856194
C	-3.5087095	20.2355325	-9.0719043
C	-2.5469383	19.8086329	-9.9598405
C	-1.2650680	20.0218633	-9.3170121
N	-1.4446155	20.5570217	-8.0658981
C	0.0000000	19.7170696	-9.9091393
C	1.2650680	20.0218633	-9.3170121
C	2.5469383	19.8086329	-9.9598405
C	3.5087095	20.2355325	-9.0719043
C	2.8105056	20.6885014	-7.8856194
N	1.4446155	20.5570217	-8.0658981
C	3.4308511	21.1428780	-6.7130646
H	2.6817671	22.3580501	-2.2793476
H	4.5974614	21.9481188	-4.1894338
H	-2.6817671	22.3580501	-2.2793476
H	-4.5974614	21.9481188	-4.1894338
H	-4.5974981	20.2355975	-9.2017873
H	-2.6818312	19.3917033	-10.9636422
H	2.6818312	19.3917033	-10.9636422
H	4.5974981	20.2355975	-9.2017873
C	0.0000000	-4.8841025	20.5845070
C	0.0000000	-6.1039328	20.3408399
C	-0.0000000	-7.4097370	19.9926398
C	-0.0000000	-8.5877307	19.5918937
C	-4.8841025	0.0000000	20.5845070
C	-6.1039328	0.0000000	20.3408399
C	-7.4097370	0.0000000	19.9926398
C	-8.5877307	0.0000000	19.5918937
C	0.0000000	4.8841025	20.5845070
C	0.0000000	6.1039328	20.3408399
C	0.0000000	7.4097370	19.9926398
C	0.0000000	8.5877307	19.5918937
C	4.8841025	0.0000000	20.5845070
C	6.1039328	0.0000000	20.3408399
C	7.4097370	-0.0000000	19.9926398
C	8.5877307	-0.0000000	19.5918937
C	0.0000000	4.8841025	-20.5845070
C	0.0000000	6.1039328	-20.3408399
C	0.0000000	7.4097370	-19.9926398
C	0.0000000	8.5877307	-19.5918937
C	-4.8841025	0.0000000	-20.5845070
C	-6.1039328	0.0000000	-20.3408399
C	-7.4097370	0.0000000	-19.9926398
C	-8.5877307	0.0000000	-19.5918937
C	0.0000000	-4.8841025	-20.5845070

C	0.0000000	-6.1039328	-20.3408399
C	-0.0000000	-7.4097370	-19.9926398
C	-0.0000000	-8.5877307	-19.5918937
C	4.8841025	0.0000000	-20.5845070
C	6.1039328	0.0000000	-20.3408399
C	7.4097370	-0.0000000	-19.9926398
C	8.5877307	-0.0000000	-19.5918937
C	16.6950279	0.0000000	14.1829854
C	17.5156460	-0.0000000	13.2475004
C	18.3390332	0.0000000	12.1759691
C	19.0311446	0.0000000	11.1417629
C	-16.6950279	0.0000000	14.1829854
C	-17.5156460	0.0000000	13.2475004
C	-18.3390332	0.0000000	12.1759691
C	-19.0311446	-0.0000000	11.1417629
C	-16.6950279	0.0000000	-14.1829854
C	-17.5156460	0.0000000	-13.2475004
C	-18.3390332	0.0000000	-12.1759691
C	-19.0311446	-0.0000000	-11.1417629
C	16.6950279	0.0000000	-14.1829854
C	17.5156460	-0.0000000	-13.2475004
C	18.3390332	0.0000000	-12.1759691
C	19.0311446	0.0000000	-11.1417629
C	22.1794545	0.0000000	1.9174108
C	22.2613377	0.0000000	0.6756806
C	22.2613377	0.0000000	-0.6756806
C	22.1794545	0.0000000	-1.9174108
C	-22.1794545	0.0000000	1.9174108
C	-22.2613377	-0.0000000	0.6756806
C	-22.2613377	-0.0000000	-0.6756806
C	-22.1794545	0.0000000	-1.9174108
C	-0.0000000	16.6950279	14.1829854
C	0.0000000	17.5156460	13.2475004
C	0.0000000	18.3390332	12.1759691
C	-0.0000000	19.0311446	11.1417629
C	0.0000000	-16.6950279	14.1829854
C	-0.0000000	-17.5156460	13.2475004
C	0.0000000	-18.3390332	12.1759691
C	0.0000000	-19.0311446	11.1417629
C	0.0000000	-16.6950279	-14.1829854
C	-0.0000000	-17.5156460	-13.2475004
C	0.0000000	-18.3390332	-12.1759691
C	0.0000000	-19.0311446	-11.1417629
C	-0.0000000	16.6950279	-14.1829854
C	0.0000000	17.5156460	-13.2475004
C	0.0000000	18.3390332	-12.1759691
C	-0.0000000	19.0311446	-11.1417629
C	-0.0000000	22.1794545	1.9174108
C	-0.0000000	22.2613377	0.6756806

C	-0.0000000	22.2613377	-0.6756806
C	0.0000000	22.1794545	-1.9174108
C	0.0000000	-22.1794545	1.9174108
C	0.0000000	-22.2613377	0.6756806
C	0.0000000	-22.2613377	-0.6756806
C	0.0000000	-22.1794545	-1.9174108
H	12.9815815	-4.5301061	17.3979071
H	12.9815815	4.5301061	17.3979071
H	-12.9815815	-4.5301061	17.3979071
H	-12.9815815	4.5301061	17.3979071
H	-12.9815815	4.5301061	-17.3979071
H	-12.9815815	-4.5301061	-17.3979071
H	12.9815815	4.5301061	-17.3979071
H	12.9815815	-4.5301061	-17.3979071
H	21.1850558	-4.5299994	6.7274202
H	21.1850558	4.5299994	6.7274202
H	-21.1850558	4.5299994	6.7274202
H	-21.1850558	-4.5299994	6.7274202
H	-21.1850558	-4.5299994	-6.7274202
H	-21.1850558	4.5299994	-6.7274202
H	21.1850558	4.5299994	-6.7274202
H	21.1850558	-4.5299994	-6.7274202
H	-4.5301061	12.9815815	17.3979071
H	4.5301061	12.9815815	17.3979071
H	4.5301061	-12.9815815	17.3979071
H	-4.5301061	-12.9815815	17.3979071
H	-4.5301061	-12.9815815	-17.3979071
H	4.5301061	-12.9815815	-17.3979071
H	4.5301061	12.9815815	-17.3979071
H	-4.5301061	12.9815815	-17.3979071
H	4.5299994	21.1850558	6.7274202
H	-4.5299994	21.1850558	6.7274202
H	-4.5299994	-21.1850558	6.7274202
H	4.5299994	-21.1850558	6.7274202
H	4.5299994	-21.1850558	-6.7274202
H	-4.5299994	-21.1850558	-6.7274202
H	-4.5299994	21.1850558	-6.7274202
H	4.5299994	21.1850558	-6.7274202

Molecule 7

1230
Energy =

Zn	5.9631137	11.8333606	16.1813880
C	5.2446466	15.5838870	14.1276982
C	3.9521045	15.2802935	14.4887669
C	4.0083706	14.0268368	15.2104472
C	6.0767400	14.5223030	14.6469481
N	5.3109232	13.5922668	15.3143232
C	7.4847441	14.4411416	14.4502826

C	8.3368703	13.4534725	15.0274399
C	9.7743488	13.4357002	14.8837617
C	10.2181116	12.2949533	15.5134236
C	9.0444679	11.6206842	16.0241020
N	7.9124049	12.3540567	15.7427688
C	9.0629950	10.3318485	16.6405911
C	7.9189955	9.6249179	17.1134645
C	7.9651027	8.3030723	17.6981701
C	6.6606795	7.9333394	17.9385531
C	5.8302480	9.0350283	17.5023446
N	6.6145497	10.0597413	17.0164354
C	4.4028547	9.0330658	17.5446267
C	3.5703169	10.1284097	17.1604800
C	2.1269605	10.1282823	17.2568363
C	1.6973112	11.3341550	16.7490113
C	2.8815779	12.0560670	16.3398682
N	4.0114675	11.3145088	16.6131795
C	2.8620363	13.3192453	15.6787945
H	5.6096604	16.4496883	13.5649897
H	3.0387681	15.8463155	14.2806022
H	10.3635345	14.2003975	14.3670063
H	11.2461115	11.9335775	15.6154191
H	8.8812730	7.7342824	17.8910088
H	6.2873868	6.9996870	18.3722124
H	1.5251714	9.3068371	17.6597353
H	0.6710425	11.7031548	16.6467979
Zn	16.1813880	5.9631137	11.8333606
C	15.5134236	10.2181116	12.2949533
C	14.8837617	9.7743488	13.4357002
C	15.0274399	8.3368703	13.4534725
C	16.0241020	9.0444679	11.6206842
N	15.7427688	7.9124049	12.3540567
C	16.6405911	9.0629950	10.3318485
C	17.1134645	7.9189955	9.6249179
C	17.6981701	7.9651027	8.3030723
C	17.9385531	6.6606795	7.9333394
C	17.5023446	5.8302480	9.0350283
N	17.0164354	6.6145497	10.0597413
C	17.5446267	4.4028547	9.0330658
C	17.1604800	3.5703169	10.1284097
C	17.2568363	2.1269605	10.1282823
C	16.7490113	1.6973112	11.3341550
C	16.3398682	2.8815779	12.0560670
N	16.6131795	4.0114675	11.3145088
C	15.6787945	2.8620363	13.3192453
C	15.2104472	4.0083706	14.0268368
C	14.4887669	3.9521045	15.2802935
C	14.1276982	5.2446466	15.5838870
C	14.6469481	6.0767400	14.5223030

N	15.3143232	5.3109232	13.5922668
C	14.4502826	7.4847441	14.4411416
H	15.6154191	11.2461115	11.9335775
H	14.3670063	10.3635345	14.2003975
H	17.8910088	8.8812730	7.7342824
H	18.3722124	6.2873868	6.9996870
H	17.6597353	1.5251714	9.3068371
H	16.6467979	0.6710425	11.7031548
H	14.2806022	3.0387681	15.8463155
H	13.5649897	5.6096604	16.4496883
Zn	11.8333606	16.1813880	5.9631137
C	7.9333394	17.9385531	6.6606795
C	8.3030723	17.6981701	7.9651027
C	9.6249179	17.1134645	7.9189955
C	9.0350283	17.5023446	5.8302480
N	10.0597413	17.0164354	6.6145497
C	9.0330658	17.5446267	4.4028547
C	10.1284097	17.1604800	3.5703169
C	10.1282823	17.2568363	2.1269605
C	11.3341550	16.7490113	1.6973112
C	12.0560670	16.3398682	2.8815779
N	11.3145088	16.6131795	4.0114675
C	13.3192453	15.6787945	2.8620363
C	14.0268368	15.2104472	4.0083706
C	15.2802935	14.4887669	3.9521045
C	15.5838870	14.1276982	5.2446466
C	14.5223030	14.6469481	6.0767400
N	13.5922668	15.3143232	5.3109232
C	14.4411416	14.4502826	7.4847441
C	13.4534725	15.0274399	8.3368703
C	13.4357002	14.8837617	9.7743488
C	12.2949533	15.5134236	10.2181116
C	11.6206842	16.0241020	9.0444679
N	12.3540567	15.7427688	7.9124049
C	10.3318485	16.6405911	9.0629950
H	6.9996870	18.3722124	6.2873868
H	7.7342824	17.8910088	8.8812730
H	9.3068371	17.6597353	1.5251714
H	11.7031548	16.6467979	0.6710425
H	15.8463155	14.2806022	3.0387681
H	16.4496883	13.5649897	5.6096604
H	14.2003975	14.3670063	10.3635345
H	11.9335775	15.6154191	11.2461115
Zn	-5.9631137	16.1813880	11.8333606
C	-6.6606795	17.9385531	7.9333394
C	-7.9651027	17.6981701	8.3030723
C	-7.9189955	17.1134645	9.6249179
C	-5.8302480	17.5023446	9.0350283
N	-6.6145497	17.0164354	10.0597413

C	-4.4028547	17.5446267	9.0330658
C	-3.5703169	17.1604800	10.1284097
C	-2.1269605	17.2568363	10.1282823
C	-1.6973112	16.7490113	11.3341550
C	-2.8815779	16.3398682	12.0560670
N	-4.0114675	16.6131795	11.3145088
C	-2.8620363	15.6787945	13.3192453
C	-4.0083706	15.2104472	14.0268368
C	-3.9521045	14.4887669	15.2802935
C	-5.2446466	14.1276982	15.5838870
C	-6.0767400	14.6469481	14.5223030
N	-5.3109232	15.3143232	13.5922668
C	-7.4847441	14.4502826	14.4411416
C	-8.3368703	15.0274399	13.4534725
C	-9.7743488	14.8837617	13.4357002
C	-10.2181116	15.5134236	12.2949533
C	-9.0444679	16.0241020	11.6206842
N	-7.9124049	15.7427688	12.3540567
C	-9.0629950	16.6405911	10.3318485
H	-6.2873868	18.3722124	6.9996870
H	-8.8812730	17.8910088	7.7342824
H	-1.5251714	17.6597353	9.3068371
H	-0.6710425	16.6467979	11.7031548
H	-3.0387681	14.2806022	15.8463155
H	-5.6096604	13.5649897	16.4496883
H	-10.3635345	14.3670063	14.2003975
H	-11.2461115	15.6154191	11.9335775
Zn	-0.0000000	-0.0000000	18.6480069
C	-0.8025557	4.2602430	18.6072143
C	-2.0977995	3.7939222	18.6078430
C	-2.0184406	2.3497719	18.6275052
C	0.0567658	3.0969989	18.6260440
N	-0.7004169	1.9445586	18.6446332
C	1.4832801	3.1534223	18.6072508
C	2.3497719	2.0184406	18.6275052
C	3.7939222	2.0977995	18.6078430
C	4.2602430	0.8025557	18.6072143
C	3.0969989	-0.0567658	18.6260440
N	1.9445586	0.7004169	18.6446332
C	3.1534223	-1.4832801	18.6072508
C	2.0184406	-2.3497719	18.6275052
C	2.0977995	-3.7939222	18.6078430
C	0.8025557	-4.2602430	18.6072143
C	-0.0567658	-3.0969989	18.6260440
N	0.7004169	-1.9445586	18.6446332
C	-1.4832801	-3.1534223	18.6072508
C	-2.3497719	-2.0184406	18.6275052
C	-3.7939222	-2.0977995	18.6078430
C	-4.2602430	-0.8025557	18.6072143

C	-3.0969989	0.0567658	18.6260440
N	-1.9445586	-0.7004169	18.6446332
C	-3.1534223	1.4832801	18.6072508
H	-0.4535966	5.2980831	18.5932337
H	-3.0280515	4.3713057	18.5936140
H	4.3713057	3.0280515	18.5936140
H	5.2980831	0.4535966	18.5932337
H	3.0280515	-4.3713057	18.5936140
H	0.4535966	-5.2980831	18.5932337
H	-4.3713057	-3.0280515	18.5936140
H	-5.2980831	-0.4535966	18.5932337
Zn	11.8333606	-5.9631137	16.1813880
C	11.3341550	-1.6973112	16.7490113
C	10.1282823	-2.1269605	17.2568363
C	10.1284097	-3.5703169	17.1604800
C	12.0560670	-2.8815779	16.3398682
N	11.3145088	-4.0114675	16.6131795
C	13.3192453	-2.8620363	15.6787945
C	14.0268368	-4.0083706	15.2104472
C	15.2802935	-3.9521045	14.4887669
C	15.5838870	-5.2446466	14.1276982
C	14.5223030	-6.0767400	14.6469481
N	13.5922668	-5.3109232	15.3143232
C	14.4411416	-7.4847441	14.4502826
C	13.4534725	-8.3368703	15.0274399
C	13.4357002	-9.7743488	14.8837617
C	12.2949533	-10.2181116	15.5134236
C	11.6206842	-9.0444679	16.0241020
N	12.3540567	-7.9124049	15.7427688
C	10.3318485	-9.0629950	16.6405911
C	9.6249179	-7.9189955	17.1134645
C	8.3030723	-7.9651027	17.6981701
C	7.9333394	-6.6606795	17.9385531
C	9.0350283	-5.8302480	17.5023446
N	10.0597413	-6.6145497	17.0164354
C	9.0330658	-4.4028547	17.5446267
H	11.7031548	-0.6710425	16.6467979
H	9.3068371	-1.5251714	17.6597353
H	15.8463155	-3.0387681	14.2806022
H	16.4496883	-5.6096604	13.5649897
H	14.2003975	-10.3635345	14.3670063
H	11.9335775	-11.2461115	15.6154191
H	7.7342824	-8.8812730	17.8910088
H	6.9996870	-6.2873868	18.3722124
Zn	18.6480069	0.0000000	0.0000000
C	18.6072143	4.2602430	0.8025557
C	18.6078430	3.7939222	2.0977995
C	18.6275052	2.3497719	2.0184406
C	18.6260440	3.0969989	-0.0567658

N	18.6446332	1.9445586	0.7004169
C	18.6072508	3.1534223	-1.4832801
C	18.6275052	2.0184406	-2.3497719
C	18.6078430	2.0977995	-3.7939222
C	18.6072143	0.8025557	-4.2602430
C	18.6260440	-0.0567658	-3.0969989
N	18.6446332	0.7004169	-1.9445586
C	18.6072508	-1.4832801	-3.1534223
C	18.6275052	-2.3497719	-2.0184406
C	18.6078430	-3.7939222	-2.0977995
C	18.6072143	-4.2602430	-0.8025557
C	18.6260440	-3.0969989	0.0567658
N	18.6446332	-1.9445586	-0.7004169
C	18.6072508	-3.1534223	1.4832801
C	18.6275052	-2.0184406	2.3497719
C	18.6078430	-2.0977995	3.7939222
C	18.6072143	-0.8025557	4.2602430
C	18.6260440	0.0567658	3.0969989
N	18.6446332	-0.7004169	1.9445586
C	18.6072508	1.4832801	3.1534223
H	18.5932337	5.2980831	0.4535966
H	18.5936140	4.3713057	3.0280515
H	18.5936140	3.0280515	-4.3713057
H	18.5932337	0.4535966	-5.2980831
H	18.5936140	-4.3713057	-3.0280515
H	18.5932337	-5.2980831	-0.4535966
H	18.5936140	-3.0280515	4.3713057
H	18.5932337	-0.4535966	5.2980831
Zn	16.1813880	11.8333606	-5.9631137
C	14.1276982	15.5838870	-5.2446466
C	14.4887669	15.2802935	-3.9521045
C	15.2104472	14.0268368	-4.0083706
C	14.6469481	14.5223030	-6.0767400
N	15.3143232	13.5922668	-5.3109232
C	14.4502826	14.4411416	-7.4847441
C	15.0274399	13.4534725	-8.3368703
C	14.8837617	13.4357002	-9.7743488
C	15.5134236	12.2949533	-10.2181116
C	16.0241020	11.6206842	-9.0444679
N	15.7427688	12.3540567	-7.9124049
C	16.6405911	10.3318485	-9.0629950
C	17.1134645	9.6249179	-7.9189955
C	17.6981701	8.3030723	-7.9651027
C	17.9385531	7.9333394	-6.6606795
C	17.5023446	9.0350283	-5.8302480
N	17.0164354	10.0597413	-6.6145497
C	17.5446267	9.0330658	-4.4028547
C	17.1604800	10.1284097	-3.5703169
C	17.2568363	10.1282823	-2.1269605

C	16.7490113	11.3341550	-1.6973112
C	16.3398682	12.0560670	-2.8815779
N	16.6131795	11.3145088	-4.0114675
C	15.6787945	13.3192453	-2.8620363
H	13.5649897	16.4496883	-5.6096604
H	14.2806022	15.8463155	-3.0387681
H	14.3670063	14.2003975	-10.3635345
H	15.6154191	11.9335775	-11.2461115
H	17.8910088	7.7342824	-8.8812730
H	18.3722124	6.9996870	-6.2873868
H	17.6597353	9.3068371	-1.5251714
H	16.6467979	11.7031548	-0.6710425
Zn	-0.0000000	18.6480069	0.0000000
C	-4.2602430	18.6072143	0.8025557
C	-3.7939222	18.6078430	2.0977995
C	-2.3497719	18.6275052	2.0184406
C	-3.0969989	18.6260440	-0.0567658
N	-1.9445586	18.6446332	0.7004169
C	-3.1534223	18.6072508	-1.4832801
C	-2.0184406	18.6275052	-2.3497719
C	-2.0977995	18.6078430	-3.7939222
C	-0.8025557	18.6072143	-4.2602430
C	0.0567658	18.6260440	-3.0969989
N	-0.7004169	18.6446332	-1.9445586
C	1.4832801	18.6072508	-3.1534223
C	2.3497719	18.6275052	-2.0184406
C	3.7939222	18.6078430	-2.0977995
C	4.2602430	18.6072143	-0.8025557
C	3.0969989	18.6260440	0.0567658
N	1.9445586	18.6446332	-0.7004169
C	3.1534223	18.6072508	1.4832801
C	2.0184406	18.6275052	2.3497719
C	2.0977995	18.6078430	3.7939222
C	0.8025557	18.6072143	4.2602430
C	-0.0567658	18.6260440	3.0969989
N	0.7004169	18.6446332	1.9445586
C	-1.4832801	18.6072508	3.1534223
H	-5.2980831	18.5932337	0.4535966
H	-4.3713057	18.5936140	3.0280515
H	-3.0280515	18.5936140	-4.3713057
H	-0.4535966	18.5932337	-5.2980831
H	4.3713057	18.5936140	-3.0280515
H	5.2980831	18.5932337	-0.4535966
H	3.0280515	18.5936140	4.3713057
H	0.4535966	18.5932337	5.2980831
Zn	-16.1813880	11.8333606	5.9631137
C	-16.7490113	11.3341550	1.6973112
C	-17.2568363	10.1282823	2.1269605
C	-17.1604800	10.1284097	3.5703169

C	-16.3398682	12.0560670	2.8815779
N	-16.6131795	11.3145088	4.0114675
C	-15.6787945	13.3192453	2.8620363
C	-15.2104472	14.0268368	4.0083706
C	-14.4887669	15.2802935	3.9521045
C	-14.1276982	15.5838870	5.2446466
C	-14.6469481	14.5223030	6.0767400
N	-15.3143232	13.5922668	5.3109232
C	-14.4502826	14.4411416	7.4847441
C	-15.0274399	13.4534725	8.3368703
C	-14.8837617	13.4357002	9.7743488
C	-15.5134236	12.2949533	10.2181116
C	-16.0241020	11.6206842	9.0444679
N	-15.7427688	12.3540567	7.9124049
C	-16.6405911	10.3318485	9.0629950
C	-17.1134645	9.6249179	7.9189955
C	-17.6981701	8.3030723	7.9651027
C	-17.9385531	7.9333394	6.6606795
C	-17.5023446	9.0350283	5.8302480
N	-17.0164354	10.0597413	6.6145497
C	-17.5446267	9.0330658	4.4028547
H	-16.6467979	11.7031548	0.6710425
H	-17.6597353	9.3068371	1.5251714
H	-14.2806022	15.8463155	3.0387681
H	-13.5649897	16.4496883	5.6096604
H	-14.3670063	14.2003975	10.3635345
H	-15.6154191	11.9335775	11.2461115
H	-17.8910088	7.7342824	8.8812730
H	-18.3722124	6.9996870	6.2873868
Zn	-11.8333606	5.9631137	16.1813880
C	-15.5838870	5.2446466	14.1276982
C	-15.2802935	3.9521045	14.4887669
C	-14.0268368	4.0083706	15.2104472
C	-14.5223030	6.0767400	14.6469481
N	-13.5922668	5.3109232	15.3143232
C	-14.4411416	7.4847441	14.4502826
C	-13.4534725	8.3368703	15.0274399
C	-13.4357002	9.7743488	14.8837617
C	-12.2949533	10.2181116	15.5134236
C	-11.6206842	9.0444679	16.0241020
N	-12.3540567	7.9124049	15.7427688
C	-10.3318485	9.0629950	16.6405911
C	-9.6249179	7.9189955	17.1134645
C	-8.3030723	7.9651027	17.6981701
C	-7.9333394	6.6606795	17.9385531
C	-9.0350283	5.8302480	17.5023446
N	-10.0597413	6.6145497	17.0164354
C	-9.0330658	4.4028547	17.5446267
C	-10.1284097	3.5703169	17.1604800

C	-10.1282823	2.1269605	17.2568363
C	-11.3341550	1.6973112	16.7490113
C	-12.0560670	2.8815779	16.3398682
N	-11.3145088	4.0114675	16.6131795
C	-13.3192453	2.8620363	15.6787945
H	-16.4496883	5.6096604	13.5649897
H	-15.8463155	3.0387681	14.2806022
H	-14.2003975	10.3635345	14.3670063
H	-11.9335775	11.2461115	15.6154191
H	-7.7342824	8.8812730	17.8910088
H	-6.9996870	6.2873868	18.3722124
H	-9.3068371	1.5251714	17.6597353
H	-11.7031548	0.6710425	16.6467979
Zn	-5.9631137	-11.8333606	16.1813880
C	-6.6606795	-7.9333394	17.9385531
C	-7.9651027	-8.3030723	17.6981701
C	-7.9189955	-9.6249179	17.1134645
C	-5.8302480	-9.0350283	17.5023446
N	-6.6145497	-10.0597413	17.0164354
C	-4.4028547	-9.0330658	17.5446267
C	-3.5703169	-10.1284097	17.1604800
C	-2.1269605	-10.1282823	17.2568363
C	-1.6973112	-11.3341550	16.7490113
C	-2.8815779	-12.0560670	16.3398682
N	-4.0114675	-11.3145088	16.6131795
C	-2.8620363	-13.3192453	15.6787945
C	-4.0083706	-14.0268368	15.2104472
C	-3.9521045	-15.2802935	14.4887669
C	-5.2446466	-15.5838870	14.1276982
C	-6.0767400	-14.5223030	14.6469481
N	-5.3109232	-13.5922668	15.3143232
C	-7.4847441	-14.4411416	14.4502826
C	-8.3368703	-13.4534725	15.0274399
C	-9.7743488	-13.4357002	14.8837617
C	-10.2181116	-12.2949533	15.5134236
C	-9.0444679	-11.6206842	16.0241020
N	-7.9124049	-12.3540567	15.7427688
C	-9.0629950	-10.3318485	16.6405911
H	-6.2873868	-6.9996870	18.3722124
H	-8.8812730	-7.7342824	17.8910088
H	-1.5251714	-9.3068371	17.6597353
H	-0.6710425	-11.7031548	16.6467979
H	-3.0387681	-15.8463155	14.2806022
H	-5.6096604	-16.4496883	13.5649897
H	-10.3635345	-14.2003975	14.3670063
H	-11.2461115	-11.9335775	15.6154191
Zn	5.9631137	-16.1813880	11.8333606
C	5.2446466	-14.1276982	15.5838870
C	3.9521045	-14.4887669	15.2802935

C	4.0083706	-15.2104472	14.0268368
C	6.0767400	-14.6469481	14.5223030
N	5.3109232	-15.3143232	13.5922668
C	7.4847441	-14.4502826	14.4411416
C	8.3368703	-15.0274399	13.4534725
C	9.7743488	-14.8837617	13.4357002
C	10.2181116	-15.5134236	12.2949533
C	9.0444679	-16.0241020	11.6206842
N	7.9124049	-15.7427688	12.3540567
C	9.0629950	-16.6405911	10.3318485
C	7.9189955	-17.1134645	9.6249179
C	7.9651027	-17.6981701	8.3030723
C	6.6606795	-17.9385531	7.9333394
C	5.8302480	-17.5023446	9.0350283
N	6.6145497	-17.0164354	10.0597413
C	4.4028547	-17.5446267	9.0330658
C	3.5703169	-17.1604800	10.1284097
C	2.1269605	-17.2568363	10.1282823
C	1.6973112	-16.7490113	11.3341550
C	2.8815779	-16.3398682	12.0560670
N	4.0114675	-16.6131795	11.3145088
C	2.8620363	-15.6787945	13.3192453
H	5.6096604	-13.5649897	16.4496883
H	3.0387681	-14.2806022	15.8463155
H	10.3635345	-14.3670063	14.2003975
H	11.2461115	-15.6154191	11.9335775
H	8.8812730	-17.8910088	7.7342824
H	6.2873868	-18.3722124	6.9996870
H	1.5251714	-17.6597353	9.3068371
H	0.6710425	-16.6467979	11.7031548
Zn	16.1813880	-11.8333606	5.9631137
C	17.9385531	-7.9333394	6.6606795
C	17.6981701	-8.3030723	7.9651027
C	17.1134645	-9.6249179	7.9189955
C	17.5023446	-9.0350283	5.8302480
N	17.0164354	-10.0597413	6.6145497
C	17.5446267	-9.0330658	4.4028547
C	17.1604800	-10.1284097	3.5703169
C	17.2568363	-10.1282823	2.1269605
C	16.7490113	-11.3341550	1.6973112
C	16.3398682	-12.0560670	2.8815779
N	16.6131795	-11.3145088	4.0114675
C	15.6787945	-13.3192453	2.8620363
C	15.2104472	-14.0268368	4.0083706
C	14.4887669	-15.2802935	3.9521045
C	14.1276982	-15.5838870	5.2446466
C	14.6469481	-14.5223030	6.0767400
N	15.3143232	-13.5922668	5.3109232
C	14.4502826	-14.4411416	7.4847441

C	15.0274399	-13.4534725	8.3368703
C	14.8837617	-13.4357002	9.7743488
C	15.5134236	-12.2949533	10.2181116
C	16.0241020	-11.6206842	9.0444679
N	15.7427688	-12.3540567	7.9124049
C	16.6405911	-10.3318485	9.0629950
H	18.3722124	-6.9996870	6.2873868
H	17.8910088	-7.7342824	8.8812730
H	17.6597353	-9.3068371	1.5251714
H	16.6467979	-11.7031548	0.6710425
H	14.2806022	-15.8463155	3.0387681
H	13.5649897	-16.4496883	5.6096604
H	14.3670063	-14.2003975	10.3635345
H	15.6154191	-11.9335775	11.2461115
Zn	16.1813880	-5.9631137	-11.8333606
C	16.7490113	-1.6973112	-11.3341550
C	17.2568363	-2.1269605	-10.1282823
C	17.1604800	-3.5703169	-10.1284097
C	16.3398682	-2.8815779	-12.0560670
N	16.6131795	-4.0114675	-11.3145088
C	15.6787945	-2.8620363	-13.3192453
C	15.2104472	-4.0083706	-14.0268368
C	14.4887669	-3.9521045	-15.2802935
C	14.1276982	-5.2446466	-15.5838870
C	14.6469481	-6.0767400	-14.5223030
N	15.3143232	-5.3109232	-13.5922668
C	14.4502826	-7.4847441	-14.4411416
C	15.0274399	-8.3368703	-13.4534725
C	14.8837617	-9.7743488	-13.4357002
C	15.5134236	-10.2181116	-12.2949533
C	16.0241020	-9.0444679	-11.6206842
N	15.7427688	-7.9124049	-12.3540567
C	16.6405911	-9.0629950	-10.3318485
C	17.1134645	-7.9189955	-9.6249179
C	17.6981701	-7.9651027	-8.3030723
C	17.9385531	-6.6606795	-7.9333394
C	17.5023446	-5.8302480	-9.0350283
N	17.0164354	-6.6145497	-10.0597413
C	17.5446267	-4.4028547	-9.0330658
H	16.6467979	-0.6710425	-11.7031548
H	17.6597353	-1.5251714	-9.3068371
H	14.2806022	-3.0387681	-15.8463155
H	13.5649897	-5.6096604	-16.4496883
H	14.3670063	-10.3635345	-14.2003975
H	15.6154191	-11.2461115	-11.9335775
H	17.8910088	-8.8812730	-7.7342824
H	18.3722124	-6.2873868	-6.9996870
Zn	11.8333606	5.9631137	-16.1813880
C	7.9333394	6.6606795	-17.9385531

C	8.3030723	7.9651027	-17.6981701
C	9.6249179	7.9189955	-17.1134645
C	9.0350283	5.8302480	-17.5023446
N	10.0597413	6.6145497	-17.0164354
C	9.0330658	4.4028547	-17.5446267
C	10.1284097	3.5703169	-17.1604800
C	10.1282823	2.1269605	-17.2568363
C	11.3341550	1.6973112	-16.7490113
C	12.0560670	2.8815779	-16.3398682
N	11.3145088	4.0114675	-16.6131795
C	13.3192453	2.8620363	-15.6787945
C	14.0268368	4.0083706	-15.2104472
C	15.2802935	3.9521045	-14.4887669
C	15.5838870	5.2446466	-14.1276982
C	14.5223030	6.0767400	-14.6469481
N	13.5922668	5.3109232	-15.3143232
C	14.4411416	7.4847441	-14.4502826
C	13.4534725	8.3368703	-15.0274399
C	13.4357002	9.7743488	-14.8837617
C	12.2949533	10.2181116	-15.5134236
C	11.6206842	9.0444679	-16.0241020
N	12.3540567	7.9124049	-15.7427688
C	10.3318485	9.0629950	-16.6405911
H	6.9996870	6.2873868	-18.3722124
H	7.7342824	8.8812730	-17.8910088
H	9.3068371	1.5251714	-17.6597353
H	11.7031548	0.6710425	-16.6467979
H	15.8463155	3.0387681	-14.2806022
H	16.4496883	5.6096604	-13.5649897
H	14.2003975	10.3635345	-14.3670063
H	11.9335775	11.2461115	-15.6154191
Zn	5.9631137	16.1813880	-11.8333606
C	1.6973112	16.7490113	-11.3341550
C	2.1269605	17.2568363	-10.1282823
C	3.5703169	17.1604800	-10.1284097
C	2.8815779	16.3398682	-12.0560670
N	4.0114675	16.6131795	-11.3145088
C	2.8620363	15.6787945	-13.3192453
C	4.0083706	15.2104472	-14.0268368
C	3.9521045	14.4887669	-15.2802935
C	5.2446466	14.1276982	-15.5838870
C	6.0767400	14.6469481	-14.5223030
N	5.3109232	15.3143232	-13.5922668
C	7.4847441	14.4502826	-14.4411416
C	8.3368703	15.0274399	-13.4534725
C	9.7743488	14.8837617	-13.4357002
C	10.2181116	15.5134236	-12.2949533
C	9.0444679	16.0241020	-11.6206842
N	7.9124049	15.7427688	-12.3540567

C	9.0629950	16.6405911	-10.3318485
C	7.9189955	17.1134645	-9.6249179
C	7.9651027	17.6981701	-8.3030723
C	6.6606795	17.9385531	-7.9333394
C	5.8302480	17.5023446	-9.0350283
N	6.6145497	17.0164354	-10.0597413
C	4.4028547	17.5446267	-9.0330658
H	0.6710425	16.6467979	-11.7031548
H	1.5251714	17.6597353	-9.3068371
H	3.0387681	14.2806022	-15.8463155
H	5.6096604	13.5649897	-16.4496883
H	10.3635345	14.3670063	-14.2003975
H	11.2461115	15.6154191	-11.9335775
H	8.8812730	17.8910088	-7.7342824
H	6.2873868	18.3722124	-6.9996870
Zn	-11.8333606	16.1813880	-5.9631137
C	-15.5838870	14.1276982	-5.2446466
C	-15.2802935	14.4887669	-3.9521045
C	-14.0268368	15.2104472	-4.0083706
C	-14.5223030	14.6469481	-6.0767400
N	-13.5922668	15.3143232	-5.3109232
C	-14.4411416	14.4502826	-7.4847441
C	-13.4534725	15.0274399	-8.3368703
C	-13.4357002	14.8837617	-9.7743488
C	-12.2949533	15.5134236	-10.2181116
C	-11.6206842	16.0241020	-9.0444679
N	-12.3540567	15.7427688	-7.9124049
C	-10.3318485	16.6405911	-9.0629950
C	-9.6249179	17.1134645	-7.9189955
C	-8.3030723	17.6981701	-7.9651027
C	-7.9333394	17.9385531	-6.6606795
C	-9.0350283	17.5023446	-5.8302480
N	-10.0597413	17.0164354	-6.6145497
C	-9.0330658	17.5446267	-4.4028547
C	-10.1284097	17.1604800	-3.5703169
C	-10.1282823	17.2568363	-2.1269605
C	-11.3341550	16.7490113	-1.6973112
C	-12.0560670	16.3398682	-2.8815779
N	-11.3145088	16.6131795	-4.0114675
C	-13.3192453	15.6787945	-2.8620363
H	-16.4496883	13.5649897	-5.6096604
H	-15.8463155	14.2806022	-3.0387681
H	-14.2003975	14.3670063	-10.3635345
H	-11.9335775	15.6154191	-11.2461115
H	-7.7342824	17.8910088	-8.8812730
H	-6.9996870	18.3722124	-6.2873868
H	-9.3068371	17.6597353	-1.5251714
H	-11.7031548	16.6467979	-0.6710425
Zn	-18.6480069	-0.0000000	-0.0000000

C	-18.6072143	-0.8025557	-4.2602430
C	-18.6078430	-2.0977995	-3.7939222
C	-18.6275052	-2.0184406	-2.3497719
C	-18.6260440	0.0567658	-3.0969989
N	-18.6446332	-0.7004169	-1.9445586
C	-18.6072508	1.4832801	-3.1534223
C	-18.6275052	2.3497719	-2.0184406
C	-18.6078430	3.7939222	-2.0977995
C	-18.6072143	4.2602430	-0.8025557
C	-18.6260440	3.0969989	0.0567658
N	-18.6446332	1.9445586	-0.7004169
C	-18.6072508	3.1534223	1.4832801
C	-18.6275052	2.0184406	2.3497719
C	-18.6078430	2.0977995	3.7939222
C	-18.6072143	0.8025557	4.2602430
C	-18.6260440	-0.0567658	3.0969989
N	-18.6446332	0.7004169	1.9445586
C	-18.6072508	-1.4832801	3.1534223
C	-18.6275052	-2.3497719	2.0184406
C	-18.6078430	-3.7939222	2.0977995
C	-18.6072143	-4.2602430	0.8025557
C	-18.6260440	-3.0969989	-0.0567658
N	-18.6446332	-1.9445586	0.7004169
C	-18.6072508	-3.1534223	-1.4832801
H	-18.5932337	-0.4535966	-5.2980831
H	-18.5936140	-3.0280515	-4.3713057
H	-18.5936140	4.3713057	-3.0280515
H	-18.5932337	5.2980831	-0.4535966
H	-18.5936140	3.0280515	4.3713057
H	-18.5932337	0.4535966	5.2980831
H	-18.5936140	-4.3713057	3.0280515
H	-18.5932337	-5.2980831	0.4535966
Zn	-16.1813880	-5.9631137	11.8333606
C	-16.7490113	-1.6973112	11.3341550
C	-17.2568363	-2.1269605	10.1282823
C	-17.1604800	-3.5703169	10.1284097
C	-16.3398682	-2.8815779	12.0560670
N	-16.6131795	-4.0114675	11.3145088
C	-15.6787945	-2.8620363	13.3192453
C	-15.2104472	-4.0083706	14.0268368
C	-14.4887669	-3.9521045	15.2802935
C	-14.1276982	-5.2446466	15.5838870
C	-14.6469481	-6.0767400	14.5223030
N	-15.3143232	-5.3109232	13.5922668
C	-14.4502826	-7.4847441	14.4411416
C	-15.0274399	-8.3368703	13.4534725
C	-14.8837617	-9.7743488	13.4357002
C	-15.5134236	-10.2181116	12.2949533
C	-16.0241020	-9.0444679	11.6206842

N	-15.7427688	-7.9124049	12.3540567
C	-16.6405911	-9.0629950	10.3318485
C	-17.1134645	-7.9189955	9.6249179
C	-17.6981701	-7.9651027	8.3030723
C	-17.9385531	-6.6606795	7.9333394
C	-17.5023446	-5.8302480	9.0350283
N	-17.0164354	-6.6145497	10.0597413
C	-17.5446267	-4.4028547	9.0330658
H	-16.6467979	-0.6710425	11.7031548
H	-17.6597353	-1.5251714	9.3068371
H	-14.2806022	-3.0387681	15.8463155
H	-13.5649897	-5.6096604	16.4496883
H	-14.3670063	-10.3635345	14.2003975
H	-15.6154191	-11.2461115	11.9335775
H	-17.8910088	-8.8812730	7.7342824
H	-18.3722124	-6.2873868	6.9996870
Zn	-11.8333606	-16.1813880	5.9631137
C	-15.5838870	-14.1276982	5.2446466
C	-15.2802935	-14.4887669	3.9521045
C	-14.0268368	-15.2104472	4.0083706
C	-14.5223030	-14.6469481	6.0767400
N	-13.5922668	-15.3143232	5.3109232
C	-14.4411416	-14.4502826	7.4847441
C	-13.4534725	-15.0274399	8.3368703
C	-13.4357002	-14.8837617	9.7743488
C	-12.2949533	-15.5134236	10.2181116
C	-11.6206842	-16.0241020	9.0444679
N	-12.3540567	-15.7427688	7.9124049
C	-10.3318485	-16.6405911	9.0629950
C	-9.6249179	-17.1134645	7.9189955
C	-8.3030723	-17.6981701	7.9651027
C	-7.9333394	-17.9385531	6.6606795
C	-9.0350283	-17.5023446	5.8302480
N	-10.0597413	-17.0164354	6.6145497
C	-9.0330658	-17.5446267	4.4028547
C	-10.1284097	-17.1604800	3.5703169
C	-10.1282823	-17.2568363	2.1269605
C	-11.3341550	-16.7490113	1.6973112
C	-12.0560670	-16.3398682	2.8815779
N	-11.3145088	-16.6131795	4.0114675
C	-13.3192453	-15.6787945	2.8620363
H	-16.4496883	-13.5649897	5.6096604
H	-15.8463155	-14.2806022	3.0387681
H	-14.2003975	-14.3670063	10.3635345
H	-11.9335775	-15.6154191	11.2461115
H	-7.7342824	-17.8910088	8.8812730
H	-6.9996870	-18.3722124	6.2873868
H	-9.3068371	-17.6597353	1.5251714
H	-11.7031548	-16.6467979	0.6710425

Zn	-0.0000000	-18.6480069	-0.0000000
C	-0.8025557	-18.6072143	4.2602430
C	-2.0977995	-18.6078430	3.7939222
C	-2.0184406	-18.6275052	2.3497719
C	0.0567658	-18.6260440	3.0969989
N	-0.7004169	-18.6446332	1.9445586
C	1.4832801	-18.6072508	3.1534223
C	2.3497719	-18.6275052	2.0184406
C	3.7939222	-18.6078430	2.0977995
C	4.2602430	-18.6072143	0.8025557
C	3.0969989	-18.6260440	-0.0567658
N	1.9445586	-18.6446332	0.7004169
C	3.1534223	-18.6072508	-1.4832801
C	2.0184406	-18.6275052	-2.3497719
C	2.0977995	-18.6078430	-3.7939222
C	0.8025557	-18.6072143	-4.2602430
C	-0.0567658	-18.6260440	-3.0969989
N	0.7004169	-18.6446332	-1.9445586
C	-1.4832801	-18.6072508	-3.1534223
C	-2.3497719	-18.6275052	-2.0184406
C	-3.7939222	-18.6078430	-2.0977995
C	-4.2602430	-18.6072143	-0.8025557
C	-3.0969989	-18.6260440	0.0567658
N	-1.9445586	-18.6446332	-0.7004169
C	-3.1534223	-18.6072508	1.4832801
H	-0.4535966	-18.5932337	5.2980831
H	-3.0280515	-18.5936140	4.3713057
H	4.3713057	-18.5936140	3.0280515
H	5.2980831	-18.5932337	0.4535966
H	3.0280515	-18.5936140	-4.3713057
H	0.4535966	-18.5932337	-5.2980831
H	-4.3713057	-18.5936140	-3.0280515
H	-5.2980831	-18.5932337	-0.4535966
Zn	11.8333606	-16.1813880	-5.9631137
C	15.5838870	-14.1276982	-5.2446466
C	15.2802935	-14.4887669	-3.9521045
C	14.0268368	-15.2104472	-4.0083706
C	14.5223030	-14.6469481	-6.0767400
N	13.5922668	-15.3143232	-5.3109232
C	14.4411416	-14.4502826	-7.4847441
C	13.4534725	-15.0274399	-8.3368703
C	13.4357002	-14.8837617	-9.7743488
C	12.2949533	-15.5134236	-10.2181116
C	11.6206842	-16.0241020	-9.0444679
N	12.3540567	-15.7427688	-7.9124049
C	10.3318485	-16.6405911	-9.0629950
C	9.6249179	-17.1134645	-7.9189955
C	8.3030723	-17.6981701	-7.9651027
C	7.9333394	-17.9385531	-6.6606795

C	9.0350283	-17.5023446	-5.8302480
N	10.0597413	-17.0164354	-6.6145497
C	9.0330658	-17.5446267	-4.4028547
C	10.1284097	-17.1604800	-3.5703169
C	10.1282823	-17.2568363	-2.1269605
C	11.3341550	-16.7490113	-1.6973112
C	12.0560670	-16.3398682	-2.8815779
N	11.3145088	-16.6131795	-4.0114675
C	13.3192453	-15.6787945	-2.8620363
H	16.4496883	-13.5649897	-5.6096604
H	15.8463155	-14.2806022	-3.0387681
H	14.2003975	-14.3670063	-10.3635345
H	11.9335775	-15.6154191	-11.2461115
H	7.7342824	-17.8910088	-8.8812730
H	6.9996870	-18.3722124	-6.2873868
H	9.3068371	-17.6597353	-1.5251714
H	11.7031548	-16.6467979	-0.6710425
Zn	5.9631137	-11.8333606	-16.1813880
C	6.6606795	-7.9333394	-17.9385531
C	7.9651027	-8.3030723	-17.6981701
C	7.9189955	-9.6249179	-17.1134645
C	5.8302480	-9.0350283	-17.5023446
N	6.6145497	-10.0597413	-17.0164354
C	4.4028547	-9.0330658	-17.5446267
C	3.5703169	-10.1284097	-17.1604800
C	2.1269605	-10.1282823	-17.2568363
C	1.6973112	-11.3341550	-16.7490113
C	2.8815779	-12.0560670	-16.3398682
N	4.0114675	-11.3145088	-16.6131795
C	2.8620363	-13.3192453	-15.6787945
C	4.0083706	-14.0268368	-15.2104472
C	3.9521045	-15.2802935	-14.4887669
C	5.2446466	-15.5838870	-14.1276982
C	6.0767400	-14.5223030	-14.6469481
N	5.3109232	-13.5922668	-15.3143232
C	7.4847441	-14.4411416	-14.4502826
C	8.3368703	-13.4534725	-15.0274399
C	9.7743488	-13.4357002	-14.8837617
C	10.2181116	-12.2949533	-15.5134236
C	9.0444679	-11.6206842	-16.0241020
N	7.9124049	-12.3540567	-15.7427688
C	9.0629950	-10.3318485	-16.6405911
H	6.2873868	-6.9996870	-18.3722124
H	8.8812730	-7.7342824	-17.8910088
H	1.5251714	-9.3068371	-17.6597353
H	0.6710425	-11.7031548	-16.6467979
H	3.0387681	-15.8463155	-14.2806022
H	5.6096604	-16.4496883	-13.5649897
H	10.3635345	-14.2003975	-14.3670063

H	11.2461115	-11.9335775	-15.6154191
Zn	0.0000000	-0.0000000	-18.6480069
C	-4.2602430	0.8025557	-18.6072143
C	-3.7939222	2.0977995	-18.6078430
C	-2.3497719	2.0184406	-18.6275052
C	-3.0969989	-0.0567658	-18.6260440
N	-1.9445586	0.7004169	-18.6446332
C	-3.1534223	-1.4832801	-18.6072508
C	-2.0184406	-2.3497719	-18.6275052
C	-2.0977995	-3.7939222	-18.6078430
C	-0.8025557	-4.2602430	-18.6072143
C	0.0567658	-3.0969989	-18.6260440
N	-0.7004169	-1.9445586	-18.6446332
C	1.4832801	-3.1534223	-18.6072508
C	2.3497719	-2.0184406	-18.6275052
C	3.7939222	-2.0977995	-18.6078430
C	4.2602430	-0.8025557	-18.6072143
C	3.0969989	0.0567658	-18.6260440
N	1.9445586	-0.7004169	-18.6446332
C	3.1534223	1.4832801	-18.6072508
C	2.0184406	2.3497719	-18.6275052
C	2.0977995	3.7939222	-18.6078430
C	0.8025557	4.2602430	-18.6072143
C	-0.0567658	3.0969989	-18.6260440
N	0.7004169	1.9445586	-18.6446332
C	-1.4832801	3.1534223	-18.6072508
H	-5.2980831	0.4535966	-18.5932337
H	-4.3713057	3.0280515	-18.5936140
H	-3.0280515	-4.3713057	-18.5936140
H	-0.4535966	-5.2980831	-18.5932337
H	4.3713057	-3.0280515	-18.5936140
H	5.2980831	-0.4535966	-18.5932337
H	3.0280515	4.3713057	-18.5936140
H	0.4535966	5.2980831	-18.5932337
Zn	-5.9631137	11.8333606	-16.1813880
C	-10.2181116	12.2949533	-15.5134236
C	-9.7743488	13.4357002	-14.8837617
C	-8.3368703	13.4534725	-15.0274399
C	-9.0444679	11.6206842	-16.0241020
N	-7.9124049	12.3540567	-15.7427688
C	-9.0629950	10.3318485	-16.6405911
C	-7.9189955	9.6249179	-17.1134645
C	-7.9651027	8.3030723	-17.6981701
C	-6.6606795	7.9333394	-17.9385531
C	-5.8302480	9.0350283	-17.5023446
N	-6.6145497	10.0597413	-17.0164354
C	-4.4028547	9.0330658	-17.5446267
C	-3.5703169	10.1284097	-17.1604800
C	-2.1269605	10.1282823	-17.2568363

C	-1.6973112	11.3341550	-16.7490113
C	-2.8815779	12.0560670	-16.3398682
N	-4.0114675	11.3145088	-16.6131795
C	-2.8620363	13.3192453	-15.6787945
C	-4.0083706	14.0268368	-15.2104472
C	-3.9521045	15.2802935	-14.4887669
C	-5.2446466	15.5838870	-14.1276982
C	-6.0767400	14.5223030	-14.6469481
N	-5.3109232	13.5922668	-15.3143232
C	-7.4847441	14.4411416	-14.4502826
H	-11.2461115	11.9335775	-15.6154191
H	-10.3635345	14.2003975	-14.3670063
H	-8.8812730	7.7342824	-17.8910088
H	-6.2873868	6.9996870	-18.3722124
H	-1.5251714	9.3068371	-17.6597353
H	-0.6710425	11.7031548	-16.6467979
H	-3.0387681	15.8463155	-14.2806022
H	-5.6096604	16.4496883	-13.5649897
Zn	-16.1813880	5.9631137	-11.8333606
C	-16.7490113	1.6973112	-11.3341550
C	-17.2568363	2.1269605	-10.1282823
C	-17.1604800	3.5703169	-10.1284097
C	-16.3398682	2.8815779	-12.0560670
N	-16.6131795	4.0114675	-11.3145088
C	-15.6787945	2.8620363	-13.3192453
C	-15.2104472	4.0083706	-14.0268368
C	-14.4887669	3.9521045	-15.2802935
C	-14.1276982	5.2446466	-15.5838870
C	-14.6469481	6.0767400	-14.5223030
N	-15.3143232	5.3109232	-13.5922668
C	-14.4502826	7.4847441	-14.4411416
C	-15.0274399	8.3368703	-13.4534725
C	-14.8837617	9.7743488	-13.4357002
C	-15.5134236	10.2181116	-12.2949533
C	-16.0241020	9.0444679	-11.6206842
N	-15.7427688	7.9124049	-12.3540567
C	-16.6405911	9.0629950	-10.3318485
C	-17.1134645	7.9189955	-9.6249179
C	-17.6981701	7.9651027	-8.3030723
C	-17.9385531	6.6606795	-7.9333394
C	-17.5023446	5.8302480	-9.0350283
N	-17.0164354	6.6145497	-10.0597413
C	-17.5446267	4.4028547	-9.0330658
H	-16.6467979	0.6710425	-11.7031548
H	-17.6597353	1.5251714	-9.3068371
H	-14.2806022	3.0387681	-15.8463155
H	-13.5649897	5.6096604	-16.4496883
H	-14.3670063	10.3635345	-14.2003975
H	-15.6154191	11.2461115	-11.9335775

H	-17.8910088	8.8812730	-7.7342824
H	-18.3722124	6.2873868	-6.9996870
Zn	-16.1813880	-11.8333606	-5.9631137
C	-17.9385531	-7.9333394	-6.6606795
C	-17.6981701	-8.3030723	-7.9651027
C	-17.1134645	-9.6249179	-7.9189955
C	-17.5023446	-9.0350283	-5.8302480
N	-17.0164354	-10.0597413	-6.6145497
C	-17.5446267	-9.0330658	-4.4028547
C	-17.1604800	-10.1284097	-3.5703169
C	-17.2568363	-10.1282823	-2.1269605
C	-16.7490113	-11.3341550	-1.6973112
C	-16.3398682	-12.0560670	-2.8815779
N	-16.6131795	-11.3145088	-4.0114675
C	-15.6787945	-13.3192453	-2.8620363
C	-15.2104472	-14.0268368	-4.0083706
C	-14.4887669	-15.2802935	-3.9521045
C	-14.1276982	-15.5838870	-5.2446466
C	-14.6469481	-14.5223030	-6.0767400
N	-15.3143232	-13.5922668	-5.3109232
C	-14.4502826	-14.4411416	-7.4847441
C	-15.0274399	-13.4534725	-8.3368703
C	-14.8837617	-13.4357002	-9.7743488
C	-15.5134236	-12.2949533	-10.2181116
C	-16.0241020	-11.6206842	-9.0444679
N	-15.7427688	-12.3540567	-7.9124049
C	-16.6405911	-10.3318485	-9.0629950
H	-18.3722124	-6.9996870	-6.2873868
H	-17.8910088	-7.7342824	-8.8812730
H	-17.6597353	-9.3068371	-1.5251714
H	-16.6467979	-11.7031548	-0.6710425
H	-14.2806022	-15.8463155	-3.0387681
H	-13.5649897	-16.4496883	-5.6096604
H	-14.3670063	-14.2003975	-10.3635345
H	-15.6154191	-11.9335775	-11.2461115
Zn	-5.9631137	-16.1813880	-11.8333606
C	-1.6973112	-16.7490113	-11.3341550
C	-2.1269605	-17.2568363	-10.1282823
C	-3.5703169	-17.1604800	-10.1284097
C	-2.8815779	-16.3398682	-12.0560670
N	-4.0114675	-16.6131795	-11.3145088
C	-2.8620363	-15.6787945	-13.3192453
C	-4.0083706	-15.2104472	-14.0268368
C	-3.9521045	-14.4887669	-15.2802935
C	-5.2446466	-14.1276982	-15.5838870
C	-6.0767400	-14.6469481	-14.5223030
N	-5.3109232	-15.3143232	-13.5922668
C	-7.4847441	-14.4502826	-14.4411416
C	-8.3368703	-15.0274399	-13.4534725

C	-9.7743488	-14.8837617	-13.4357002
C	-10.2181116	-15.5134236	-12.2949533
C	-9.0444679	-16.0241020	-11.6206842
N	-7.9124049	-15.7427688	-12.3540567
C	-9.0629950	-16.6405911	-10.3318485
C	-7.9189955	-17.1134645	-9.6249179
C	-7.9651027	-17.6981701	-8.3030723
C	-6.6606795	-17.9385531	-7.9333394
C	-5.8302480	-17.5023446	-9.0350283
N	-6.6145497	-17.0164354	-10.0597413
C	-4.4028547	-17.5446267	-9.0330658
H	-0.6710425	-16.6467979	-11.7031548
H	-1.5251714	-17.6597353	-9.3068371
H	-3.0387681	-14.2806022	-15.8463155
H	-5.6096604	-13.5649897	-16.4496883
H	-10.3635345	-14.3670063	-14.2003975
H	-11.2461115	-15.6154191	-11.9335775
H	-8.8812730	-17.8910088	-7.7342824
H	-6.2873868	-18.3722124	-6.9996870
Zn	-11.8333606	-5.9631137	-16.1813880
C	-15.5838870	-5.2446466	-14.1276982
C	-15.2802935	-3.9521045	-14.4887669
C	-14.0268368	-4.0083706	-15.2104472
C	-14.5223030	-6.0767400	-14.6469481
N	-13.5922668	-5.3109232	-15.3143232
C	-14.4411416	-7.4847441	-14.4502826
C	-13.4534725	-8.3368703	-15.0274399
C	-13.4357002	-9.7743488	-14.8837617
C	-12.2949533	-10.2181116	-15.5134236
C	-11.6206842	-9.0444679	-16.0241020
N	-12.3540567	-7.9124049	-15.7427688
C	-10.3318485	-9.0629950	-16.6405911
C	-9.6249179	-7.9189955	-17.1134645
C	-8.3030723	-7.9651027	-17.6981701
C	-7.9333394	-6.6606795	-17.9385531
C	-9.0350283	-5.8302480	-17.5023446
N	-10.0597413	-6.6145497	-17.0164354
C	-9.0330658	-4.4028547	-17.5446267
C	-10.1284097	-3.5703169	-17.1604800
C	-10.1282823	-2.1269605	-17.2568363
C	-11.3341550	-1.6973112	-16.7490113
C	-12.0560670	-2.8815779	-16.3398682
N	-11.3145088	-4.0114675	-16.6131795
C	-13.3192453	-2.8620363	-15.6787945
H	-16.4496883	-5.6096604	-13.5649897
H	-15.8463155	-3.0387681	-14.2806022
H	-14.2003975	-10.3635345	-14.3670063
H	-11.9335775	-11.2461115	-15.6154191
H	-7.7342824	-8.8812730	-17.8910088

H	-6.9996870	-6.2873868	-18.3722124
H	-9.3068371	-1.5251714	-17.6597353
H	-11.7031548	-0.6710425	-16.6467979
C	8.0629394	15.3201560	13.5088956
C	8.5635330	15.9175255	12.5390576
C	9.1280631	16.3827391	11.4033125
C	9.6632965	16.6269771	10.3070886
C	10.3070886	9.6632965	16.6269771
C	11.4033125	9.1280631	16.3827391
C	12.5390576	8.5635330	15.9175255
C	13.5088956	8.0629394	15.3201560
C	3.7659148	7.8313522	17.9311949
C	3.2146862	6.7434904	18.1791409
C	2.6231473	5.5439090	18.3846427
C	2.0856168	4.4297313	18.5190103
C	1.6109429	13.8863863	15.3521203
C	0.5658519	14.4115938	14.9327210
C	-0.5658519	14.9327210	14.4115938
C	-1.6109429	15.3521203	13.8863863
C	16.6269771	10.3070886	9.6632965
C	16.3827391	11.4033125	9.1280631
C	15.9175255	12.5390576	8.5635330
C	15.3201560	13.5088956	8.0629394
C	17.9311949	3.7659148	7.8313522
C	18.1791409	3.2146862	6.7434904
C	18.3846427	2.6231473	5.5439090
C	18.5190103	2.0856168	4.4297313
C	15.3521203	1.6109429	13.8863863
C	14.9327210	0.5658519	14.4115938
C	14.4115938	-0.5658519	14.9327210
C	13.8863863	-1.6109429	15.3521203
C	7.8313522	17.9311949	3.7659148
C	6.7434904	18.1791409	3.2146862
C	5.5439090	18.3846427	2.6231473
C	4.4297313	18.5190103	2.0856168
C	13.8863863	15.3521203	1.6109429
C	14.4115938	14.9327210	0.5658519
C	14.9327210	14.4115938	-0.5658519
C	15.3521203	13.8863863	-1.6109429
C	-3.7659148	17.9311949	7.8313522
C	-3.2146862	18.1791409	6.7434904
C	-2.6231473	18.3846427	5.5439090
C	-2.0856168	18.5190103	4.4297313
C	-8.0629394	13.5088956	15.3201560
C	-8.5635330	12.5390576	15.9175255
C	-9.1280631	11.4033125	16.3827391
C	-9.6632965	10.3070886	16.6269771
C	-10.3070886	16.6269771	9.6632965
C	-11.4033125	16.3827391	9.1280631

C	-12.5390576	15.9175255	8.5635330
C	-13.5088956	15.3201560	8.0629394
C	4.4297313	-2.0856168	18.5190103
C	5.5439090	-2.6231473	18.3846427
C	6.7434904	-3.2146862	18.1791409
C	7.8313522	-3.7659148	17.9311949
C	-2.0856168	-4.4297313	18.5190103
C	-2.6231473	-5.5439090	18.3846427
C	-3.2146862	-6.7434904	18.1791409
C	-3.7659148	-7.8313522	17.9311949
C	-4.4297313	2.0856168	18.5190103
C	-5.5439090	2.6231473	18.3846427
C	-6.7434904	3.2146862	18.1791409
C	-7.8313522	3.7659148	17.9311949
C	15.3201560	-8.0629394	13.5088956
C	15.9175255	-8.5635330	12.5390576
C	16.3827391	-9.1280631	11.4033125
C	16.6269771	-9.6632965	10.3070886
C	9.6632965	-10.3070886	16.6269771
C	9.1280631	-11.4033125	16.3827391
C	8.5635330	-12.5390576	15.9175255
C	8.0629394	-13.5088956	15.3201560
C	18.5190103	4.4297313	-2.0856168
C	18.3846427	5.5439090	-2.6231473
C	18.1791409	6.7434904	-3.2146862
C	17.9311949	7.8313522	-3.7659148
C	18.5190103	-2.0856168	-4.4297313
C	18.3846427	-2.6231473	-5.5439090
C	18.1791409	-3.2146862	-6.7434904
C	17.9311949	-3.7659148	-7.8313522
C	18.5190103	-4.4297313	2.0856168
C	18.3846427	-5.5439090	2.6231473
C	18.1791409	-6.7434904	3.2146862
C	17.9311949	-7.8313522	3.7659148
C	13.5088956	15.3201560	-8.0629394
C	12.5390576	15.9175255	-8.5635330
C	11.4033125	16.3827391	-9.1280631
C	10.3070886	16.6269771	-9.6632965
C	16.6269771	9.6632965	-10.3070886
C	16.3827391	9.1280631	-11.4033125
C	15.9175255	8.5635330	-12.5390576
C	15.3201560	8.0629394	-13.5088956
C	-4.4297313	18.5190103	-2.0856168
C	-5.5439090	18.3846427	-2.6231473
C	-6.7434904	18.1791409	-3.2146862
C	-7.8313522	17.9311949	-3.7659148
C	2.0856168	18.5190103	-4.4297313
C	2.6231473	18.3846427	-5.5439090
C	3.2146862	18.1791409	-6.7434904

C	3.7659148	17.9311949	-7.8313522
C	-15.3521203	13.8863863	1.6109429
C	-14.9327210	14.4115938	0.5658519
C	-14.4115938	14.9327210	-0.5658519
C	-13.8863863	15.3521203	-1.6109429
C	-16.6269771	9.6632965	10.3070886
C	-16.3827391	9.1280631	11.4033125
C	-15.9175255	8.5635330	12.5390576
C	-15.3201560	8.0629394	13.5088956
C	-17.9311949	7.8313522	3.7659148
C	-18.1791409	6.7434904	3.2146862
C	-18.3846427	5.5439090	2.6231473
C	-18.5190103	4.4297313	2.0856168
C	-13.8863863	1.6109429	15.3521203
C	-14.4115938	0.5658519	14.9327210
C	-14.9327210	-0.5658519	14.4115938
C	-15.3521203	-1.6109429	13.8863863
C	-1.6109429	-13.8863863	15.3521203
C	-0.5658519	-14.4115938	14.9327210
C	0.5658519	-14.9327210	14.4115938
C	1.6109429	-15.3521203	13.8863863
C	-8.0629394	-15.3201560	13.5088956
C	-8.5635330	-15.9175255	12.5390576
C	-9.1280631	-16.3827391	11.4033125
C	-9.6632965	-16.6269771	10.3070886
C	-10.3070886	-9.6632965	16.6269771
C	-11.4033125	-9.1280631	16.3827391
C	-12.5390576	-8.5635330	15.9175255
C	-13.5088956	-8.0629394	15.3201560
C	10.3070886	-16.6269771	9.6632965
C	11.4033125	-16.3827391	9.1280631
C	12.5390576	-15.9175255	8.5635330
C	13.5088956	-15.3201560	8.0629394
C	3.7659148	-17.9311949	7.8313522
C	3.2146862	-18.1791409	6.7434904
C	2.6231473	-18.3846427	5.5439090
C	2.0856168	-18.5190103	4.4297313
C	15.3521203	-13.8863863	1.6109429
C	14.9327210	-14.4115938	0.5658519
C	14.4115938	-14.9327210	-0.5658519
C	13.8863863	-15.3521203	-1.6109429
C	15.3521203	-1.6109429	-13.8863863
C	14.9327210	-0.5658519	-14.4115938
C	14.4115938	0.5658519	-14.9327210
C	13.8863863	1.6109429	-15.3521203
C	13.5088956	-8.0629394	-15.3201560
C	12.5390576	-8.5635330	-15.9175255
C	11.4033125	-9.1280631	-16.3827391
C	10.3070886	-9.6632965	-16.6269771

C	16.6269771	-10.3070886	-9.6632965
C	16.3827391	-11.4033125	-9.1280631
C	15.9175255	-12.5390576	-8.5635330
C	15.3201560	-13.5088956	-8.0629394
C	7.8313522	3.7659148	-17.9311949
C	6.7434904	3.2146862	-18.1791409
C	5.5439090	2.6231473	-18.3846427
C	4.4297313	2.0856168	-18.5190103
C	9.6632965	10.3070886	-16.6269771
C	9.1280631	11.4033125	-16.3827391
C	8.5635330	12.5390576	-15.9175255
C	8.0629394	13.5088956	-15.3201560
C	1.6109429	15.3521203	-13.8863863
C	0.5658519	14.9327210	-14.4115938
C	-0.5658519	14.4115938	-14.9327210
C	-1.6109429	13.8863863	-15.3521203
C	-15.3201560	13.5088956	-8.0629394
C	-15.9175255	12.5390576	-8.5635330
C	-16.3827391	11.4033125	-9.1280631
C	-16.6269771	10.3070886	-9.6632965
C	-9.6632965	16.6269771	-10.3070886
C	-9.1280631	16.3827391	-11.4033125
C	-8.5635330	15.9175255	-12.5390576
C	-8.0629394	15.3201560	-13.5088956
C	-18.5190103	2.0856168	-4.4297313
C	-18.3846427	2.6231473	-5.5439090
C	-18.1791409	3.2146862	-6.7434904
C	-17.9311949	3.7659148	-7.8313522
C	-18.5190103	-2.0856168	4.4297313
C	-18.3846427	-2.6231473	5.5439090
C	-18.1791409	-3.2146862	6.7434904
C	-17.9311949	-3.7659148	7.8313522
C	-18.5190103	-4.4297313	-2.0856168
C	-18.3846427	-5.5439090	-2.6231473
C	-18.1791409	-6.7434904	-3.2146862
C	-17.9311949	-7.8313522	-3.7659148
C	-16.6269771	-10.3070886	9.6632965
C	-16.3827391	-11.4033125	9.1280631
C	-15.9175255	-12.5390576	8.5635330
C	-15.3201560	-13.5088956	8.0629394
C	-7.8313522	-17.9311949	3.7659148
C	-6.7434904	-18.1791409	3.2146862
C	-5.5439090	-18.3846427	2.6231473
C	-4.4297313	-18.5190103	2.0856168
C	-13.8863863	-15.3521203	1.6109429
C	-14.4115938	-14.9327210	0.5658519
C	-14.9327210	-14.4115938	-0.5658519
C	-15.3521203	-13.8863863	-1.6109429
C	4.4297313	-18.5190103	-2.0856168

C	5.5439090	-18.3846427	-2.6231473
C	6.7434904	-18.1791409	-3.2146862
C	7.8313522	-17.9311949	-3.7659148
C	-2.0856168	-18.5190103	-4.4297313
C	-2.6231473	-18.3846427	-5.5439090
C	-3.2146862	-18.1791409	-6.7434904
C	-3.7659148	-17.9311949	-7.8313522
C	9.6632965	-16.6269771	-10.3070886
C	9.1280631	-16.3827391	-11.4033125
C	8.5635330	-15.9175255	-12.5390576
C	8.0629394	-15.3201560	-13.5088956
C	3.7659148	-7.8313522	-17.9311949
C	3.2146862	-6.7434904	-18.1791409
C	2.6231473	-5.5439090	-18.3846427
C	2.0856168	-4.4297313	-18.5190103
C	1.6109429	-13.8863863	-15.3521203
C	0.5658519	-14.4115938	-14.9327210
C	-0.5658519	-14.9327210	-14.4115938
C	-1.6109429	-15.3521203	-13.8863863
C	-4.4297313	-2.0856168	-18.5190103
C	-5.5439090	-2.6231473	-18.3846427
C	-6.7434904	-3.2146862	-18.1791409
C	-7.8313522	-3.7659148	-17.9311949
C	-2.0856168	4.4297313	-18.5190103
C	-2.6231473	5.5439090	-18.3846427
C	-3.2146862	6.7434904	-18.1791409
C	-3.7659148	7.8313522	-17.9311949
C	-10.3070886	9.6632965	-16.6269771
C	-11.4033125	9.1280631	-16.3827391
C	-12.5390576	8.5635330	-15.9175255
C	-13.5088956	8.0629394	-15.3201560
C	-15.3521203	1.6109429	-13.8863863
C	-14.9327210	0.5658519	-14.4115938
C	-14.4115938	-0.5658519	-14.9327210
C	-13.8863863	-1.6109429	-15.3521203
C	-13.5088956	-15.3201560	-8.0629394
C	-12.5390576	-15.9175255	-8.5635330
C	-11.4033125	-16.3827391	-9.1280631
C	-10.3070886	-16.6269771	-9.6632965
C	-16.6269771	-9.6632965	-10.3070886
C	-16.3827391	-9.1280631	-11.4033125
C	-15.9175255	-8.5635330	-12.5390576
C	-15.3201560	-8.0629394	-13.5088956
C	-8.0629394	-13.5088956	-15.3201560
C	-8.5635330	-12.5390576	-15.9175255
C	-9.1280631	-11.4033125	-16.3827391
C	-9.6632965	-10.3070886	-16.6269771
